# Supplementary material for: Use of removable support boot versus cast for early mobilisation after ankle fracture surgery: cost-effectiveness analysis and qualitative findings of the Ankle Recovery Trial (ART)
Source: BMJ Open. 2024 Jan 12;14(1):e073542. doi: 10.1136/bmjopen-2023-073542 (PMC10810042; doi:10.1136/bmjopen-2023-073542)
Supplement: Supplementary data [file bmjopen-2023-073542supp001.pdf]

Appendix

Use of removable support boot vs plaster for early mobilisation after ankle fracture surgery: Cost-effectiveness analysis and qualitative findings of the Ankle Recovery Trial. Baji et al.

Imputation of missing data

We imputed missing cost variables, utility scores, and Olerud and Molander ankle scores (OMAS) at each time point using multiple imputation methods (21). We applied chained equations, with 50 sets and predictive mean matching, assuming that data was not missing completely at random. Our imputation model considered treatment arm, hospital site, age, sex, body mass index, relationship status (married/partnership vs other), if the participant lived alone before the injury, education (higher degree vs no higher degree), employment status (working full-time or part-time vs not working) as well as fracture complexity (complex vs not complex), baseline utility, and baseline OMAS scores.

Stata code for imputation

```
mi set wide //register dataset to be imputed
mi register imputed costphysiogp_4 costphysiohospital_4 costphysioprivate_4 /*
 */ costgp_4 costsocialcare_4 costcommunityprivate_4 /*
 */ costhospitalAEoutpatient_4 costhospitaladmission_4 /*
 */ costequipmentprivate_4 costequipmentnhs_4 costotherprivate_4 /*
 */ costmedication_4 costphysiogp_10 costphysiohospital_10 costgp_10 /*
 */ costsocialcare_10 costhospitalAEoutpatient_10 costhospitaladmission_10  /*
 */ costequipmentnhs_10 costequipmentprivate_10 costotherprivate_10  /*
 */  cost_timeoff_4 cost_informalcare_4 cost_majorimpactprivate_4   /*
 */  costmedication_10 costphysioprivate_10  /*
 */   cost_timeoff_10 cost_informalcare_10 cost_majorimpactprivate_10 cost_fitting
cost_intervention //cost variables to be imputed
mi register imputed utility_4 utility_5 utility_10 //QALY variables to be imputed
mi register imputed OreludMolander_w5 OreludMolander_w10 // outcome variables to be
imputed
mi register imputed female complexity utility_baseline OreludMolander_baseline bmi
married_dummy alone work_dummy degree // stratification variables to be imputed
mi register regular treatmentgroup age  site   //regular variables that do not require imputation

mi impute chained (pmm , knn(10)) costphysiogp_4 costphysiohospital_4
costphysioprivate_4 /*
 */ costgp_4 costsocialcare_4 costcommunityprivate_4 /*
 */ costhospitalAEoutpatient_4 costhospitaladmission_4 /*
 */ costequipmentprivate_4 costequipmentnhs_4 costotherprivate_4 /*
 */ costmedication_4 costphysiogp_10 costphysiohospital_10 costgp_10 /*
 */ costsocialcare_10 costhospitalAEoutpatient_10 costhospitaladmission_10  /*
```

```
 */ costequipmentnhs_10 costequipmentprivate_10 costotherprivate_10  /*
 */  cost_timeoff_4 cost_informalcare_4 cost_majorimpactprivate_4   /*
 */  costmedication_10 costphysioprivate_10  /*
 */   cost_timeoff_10 cost_informalcare_10 cost_majorimpactprivate_10 /*
 */ cost_fitting cost_intervention utility_4 utility_5 utility_10 /*
 */ OreludMolander_w5 OreludMolander_w10  /*
 */  female complexity utility_baseline OreludMolander_baseline bmi married_dummy alone
work_dummy degree= treatmentgroup age  site , add(50) by(treatmentgroup) rseed(10) force
```

Table S1. Unit costs

Item	Cost (£)	Source
**Physiotherapy services**		Unit Costs of Health and Social Care 2020/21 (PSSRU)
Provided by GP surgery/practice – at the surgery/practic	40.5	Community-Based Scientific and Professional Staff, cost per hour, Band 5, physiotherapist, occupational therapist, speech and language therapist, podiatrist, clinical psychology assistant practitioner (higher level) counsellor (entry level), 1 hour
Provided by GP surgery/practice – at home	41.5	Community-Based Scientific and Professional Staff, cost per hour, Band 5, physiotherapist, occupational therapist, speech and language therapist, podiatrist, clinical psychology assistant practitioner (higher level) counsellor (entry level), 1 hour
Provided by a hospital – at home	39.5	Hospital based scientific and professional, cost per hour, Band 5, Physiotherapist, Occupational therapist, Speech and language therapist, Podiatrist, Clinical psychology assistant practitioner (higher level), Counsellor (entry level), 1 hour
Provided by a private clinic - at clinic	51.8	Hospital based scientific and professional, cost per hour, Band 6, Physiotherapist, Occupational therapist, Speech and language therapist, Podiatrist, Clinical psychology assistant practitioner (higher level), Counsellor (entry level), 1 hour
Consultations with an Occupational Therapist	88.4	Community-Based Scientific and Professional Staff, cost per hour Band 8.b., Physiotherapist consultant, Occupational therapist consultant, Clinical psychologist principal, Speech and language therapist principal, Podiatric consultant (surgery), Arts therapis t principal, 1 hour
**Community-based Services**		Unit Costs of Health and Social Care 2020/21 (PSSRU)
GP at the surgery/practice	33.0	Primary care practice professionals, Cost per surgery lasting 9.22 minutes (including direct care)
GP at home	132.0	Primary care practice professionals, GP Cost per hour (including direct care)
GP telephone contact	33.0	Primary care practice professionals, Cost per surgery lasting 9.22 minutes (including direct care)
Practice Nurse / Health Care Assistant at GP surgery	42.1	Primary care practice professionals, GP Practice Nurse (cost/hour)
Practice Nurse telephone contact	42.1	Primary care practice professionals, GP Practice Nurse (cost/hour)
Nurse at home	42.1	Primary care practice professionals, GP Practice Nurse (cost/hour)
Health Visitor	34.9	Community-based scientific and professional staff, cost per hour, Band 4

Home care/Home help	23.1	Social care staff, cost per hour, Home Care Worker
Food at home services (e.g. meals on wheels)	24.6	Social care staff, cost per hour, Support and outreach worker
Voluntary Agency Worker/Contact (e.g. from Age UK)	24.6	Social care staff, cost per hour, Support and outreach worker
**Hospital Services**		
Accident and Emergency	187.8	2019-20_National_schedule_of_NHS_costs_FY19_20_V2, A&E visits weighted average, inflated to 2020/21 prices using NHSCII Pay & Prices
Day case admission or procedure (but did not spend the night)	838.0	2019-20_National_schedule_of_NHS_costs_FY19_20_V2, Day cases weighted average, inflated to 2020/21 prices using NHSCII Pay & Prices, Assuming there is a procedure
Outpatient appointment	136.9	2019-20_National_schedule_of_NHS_costs_FY19_20_V2, OP appointments weighted average, inflated to 2020/21 prices using NHSCII Pay & Prices
**Time off work (productivity loss)**	20.4	Gross earning per hour, 2021, Earnings and hours worked, age group: ASHE Table 6 - Office for National Statistics (ons.gov.uk), Table 6.5a Hourly pay - Gross (£) - For all employee jobsa: United Kingdom, 2021, Age group 40-49
**Support from others (informal care)**	8.91	Minimum wage between April 2021 to March 2022, Minimum wage rates for 2022 - GOV.UK (www.gov.uk)
**Special Equipment (NHS costs)**		
Stick	3.5	Hospital procurement, 2022
One Crutch	3.58	
Two Crutches	7.16	
Frame	18.5	
Raised toilet seat	7.2	
Perching stool	17	
Bath support	11	
Shower seat	26.77	
Comode toilet	19.83	
Toilet rails	4.2	
Commode with 2 Zimmer Frames	56.83	
**Treatment costs**		
Boot	50	Hospital procurement
Plaster	25	Hospital procurement

Plaster technician (cost per hour)		Unit Costs of Health and Social Care 2020/21 (PSSRU)
Physiotherapist Band 4	35	Hospital based scientific and professional, cost per hour
Physiotherapist Band 5	39	Hospital based scientific and professional, cost per hour
Physiotherapist Band 6	52	Hospital based scientific and professional, cost per hour
Private expenses		Gym sessions and other sport sessions, Child care, Home cleaner, Food delivery (e.g. take away, eating out), Other, Equipments bought privately - Expenses provided by participant in the questionnaire, Inflated to 2020/21 prices using CPI
**Medication cost (Prescribed&out-of-pocket)**		Source: British National Formulary for medications April 2022

**Medication**	**Unit cost 2022**	**Drug tariff price (one pill)**	**Pills/day based on dose assumption**	**Dose (assumption)**
Ibuprofen 200mg	0.118	0.039	3	maintenance 200–400 mg 3 times a day
Paracetamol 500 mg	0.095	0.024	4	500mg every 4-6 hours
Codeine phosphate 15 mg	0.176	0.029	6	30 mg 3–4 times a day; usual dose 15–60 mg 3–4 times a day
Co-codamol 500mg / 8 mg	0.152	0.038	4	every 4–6 hours
Naproxen 250 mg	0.121	0.040	3	250 mg every 6–8 hours as required
Tramadol hydrocloride 50 mg	0.475	0.119	4	50–100 mg every 4–6 hours
Omeprazole 10 mg	0.332	0.332	1	10 mg once daily,
Antibiotic (Ampicillin 250 mg)	6.946	0.868	8	0.5–1 g every 6 hours.
Dalteparin (Fragmin)*	5.122	5.122	1	5000 units every 12 hours
Amitryptyline hydrocloride 10mg	0.027	0.027	1	Initially 5–10 mg daily, to be taken at night; increased in steps of 10 mg at least every 2 weeks as required; maximum 30 mg per day
Oramorph 10 mg	0.442	0.074	6	Initially 10 mg every 4 hours (acute)
Oxycodone Hydrochlorid 10mg	0.368	0.184	2	Initially 10 mg every 12 hours
Voltaren gel	4.020			
Gabapentin 600mg	0.071	0.047	1.5	Initially 300 mg once daily on day 1, then 300 mg twice daily on day 2, then 300 mg 3 times a day on day 3, alternatively initially 300 mg 3 times a day on day 1, then increased in steps of 300 mg every 2–3 days in 3 divided

				doses, adjusted according to response; maximum 3.6 g per day.
Rivaroxaban 15mg	1.800	1.800	1	10 mg once daily
Ibuprofen gel 100g	2.400			
Co-amoxiclav 500/125mg Tablets	0.264	0.088	3	500/125 mg 3 times a day
Flucoxacillin Capsules 500mg	0.166	0.041	4	500 mg 4 times a day for 5–7 days.
Metronidazole 400mg	0.246	0.082	3	

Table S2. Resource utilisation

	**Week 3-6 post surgery**						**Week 7-12 post surgery**					
	**Plaster (N=120)**			**Boots (N=123)**			**Plaster (N=120)**			**Boots (N=123)**		
	Non-missing	Users	Average use (occasions)	Non-missing	Users	Average use (occasions)	Non-missing	Users	Average use (occasions)	Non-missing	Users	Average use (occasions)
**Physiotherapy services**												
Provided by GP surgery/practice – at the surgery	101	0	0.00	104	3	0.04	77	5	0.10	77	7	0.25
Provided by GP surgery/practice – at your home	102	0	0.00	104	1	0.03	76	0	0.00	77	0	0.00
Provided by a hospital – at hospital	102	0	0.00	105	0	0.00	76	0	0.00	77	0	0.00
Provided by a hospital – at home	102	4	0.05	105	5	0.05	76	2	0.03	77	1	0.01
Provided by a private clinic - at clinic	102	0	0.00	105	2	0.04	76	6	0.28	77	4	0.18
Provided by a private clinic - at home	102	0	0.00	105	0	0.00	76	1	0.04	77	0	0.00
Consultations with an Occupational Therapist	102	5	0.07	105	2	0.05	76	7	0.20	77	11	0.19
**Community-based Services**												
GP at the surgery/practice	102	3	0.04	105	12	0.27	75	7	0.13	75	10	0.28
GP at your home	102	0	0.00	105	2	0.02	75	0	0.00	75	1	0.01
GP telephone contact	102	14	0.20	105	16	0.29	75	5	0.11	75	10	0.19
Practice Nurse / Health Care Assistant at GP surgery	100	0	0.00	104	9	0.09	75	0	0.00	75	5	0.07
Practice Nurse telephone contact	100	0	0.00	103	4	0.07	75	0	0.00	75	0	0.00
Nurse at your home	101	3	0.58	104	5	0.31	75	0	0.00	75	0	0.00
Health Visitor	100	1	0.01	104	0	0.00	75	0	0.00	75	0	0.00
Social worker (all contacts, including telephone calls)	100	0	0.00	104	0	0.00	75	0	0.00	75	0	0.00
Home care/Home help	100	1	0.01	104	4	0.20	75	0	0.00	75	0	0.00

Food at home services (e.g. meals on wheels)	100	0	0.00	104	3	0.09	75	0	0.00	75	74	3.95
Voluntary Agency Worker/Contact	100	0	0.00	103	1	0.02	75	0	0.00	75	0	0.00
**Hospital Services**												
Accident and Emergency	102	5	0.05	105	4	0.05	75	0	0.00	76	0	0.00
Day case admission or procedure (but did not spend the night)	102	1	0.01	105	1	0.01	75	0	0.00	76	0	0.00
Admitted to hospital overnight	98	0	0.00	102	0	0.00	75	0	0.00	76	0	0.00
Outpatient appointment	99	9	0.10	104	8	0.12	75	11	0.21	76	13	0.00
**Informal care (hours)**	102	81	70.33	105	88	43.92	78	47	33.28	78	49	51.65
**Time off work (hours)**	102	48	67.56	105	60	71.91	77	27	58.71	77	29	39.64
**Use of special equipment**	Non-missing	Users	%	Non-missing	Users	%	Non-missing	Users	%	Non-missing	Users	%
Special equipment private	99	2	2.0%	104	8	7.7%	75	0	0.0%	76	1	1.3%
Special equipment NHS (prescribed)	99	6	6.1%	104	3	2.9%	77	12	15.6%	77	22	28.6%
**Use of medication (Prescribed&out-of-pocket)**	101	45	44.6%	105	64	61.0%	76	34	44.7%	77	36	46.8%
**Private costs**												
Gym sessions and other sport sessions	100	1	1.0%	105	6	5.7%	77	4	5.2%	77	6	7.8%
Child care	100	4	4.0%	105	4	3.8%	76	1	1.3%	77	0	0.0%
Home cleaner	100	9	9.0%	105	16	15.2%	76	3	3.9%	77	4	5.2%
Food delivery (e.g. take away, eating out)	100	23	23.0%	105	21	20.0%	76	5	6.6%	77	9	11.7%
Other	99	3	3.0%	103	16	15.5%	75	1	1.3%	77	4	5.2%
Travel expenses	100	34	34.0%	105	34	32.4%	76	21	27.6%	77	19	24.7%
Other major private expenses	101	4	4.0%	105	3	2.9%	77	4	5.2%	75	5	6.7%

Table S3 Costs and outcomes by trial arm and by perspective on costs (raw data)

**Cost Component**		**Boot (N=123)**		**Plaster (N=120)**	**Difference**
NHS+PSS	n	Mean (95% CI)	n	Mean (95% CI)	Mean (95% CI)
Ankle treatment (boot or plaster)	117	£203 (£200, £206)	112	£179 (£177, £181)	£24 (£21, £28)
Physiotherapy in the community	70	£15 (£7, £23)	68	£4 (£1, £7)	£11 (£3, £20)
Physiotherapy in hospital	70	£24 (£13, £35)	68	£25 (£11, £39)	-£1 (-£19, £16)
GP practice services	68	£69 (£37, £100)	66	£33 (£7, £59)	£36 (-£5, £77)
Social Care services	68	£24 (£11, £36)	66	£7 (-£0, £14)	£17 (£2, £31)
A&E and Outpatient services	68	£60 (£24, £96)	65	£29 (£17, £40)	£31 (-£7, £70)
Hospital Admissions	66	£14 (-£6, £34)	66	£14 (-£6, £34)	£0 (-£28, £28)
NHS Equipment	69	£4 (£2, £6)	68	£2 (£1, £2)	£2 (-£0, £4)
Medications	70	£6 (£4, £8)	68	£6 (£1, £10)	£1 (-£4, £5)
Societal costs					
Private physiotherapy	70	£18 (£5, £30)	68	£19 (£7, £31)	-£2 (-£19, £16)
Additional medical Equipment	68	£8 (£3, £13)	66	£0 (-£0, £1)	£8 (£3, £12)
Private community care	104	£0 (-£0, £1)	100	£2 (-£1, £4)	-£1 (-£4, £2)
Other private expenditures on services/activities	67	£105 (£74, £136)	67	£170 (£30, £311)	-£65 (-£207, £77)
Other major expenditure not reported elsewhere	68	£11 (-£0, £23)	68	£11 (£2, £21)	-£0 (-£15, £15)
Productivity Loss (time-off work and leisure)	70	£1,915 (£1,493, £2,337)	69	£2,164 (£1,565, £2,764)	-£249 (-£976, £478)
Informal Care	71	£1,042 (£652, £1,432)	70	£1,185 (£719, £1,650)	-£143 (-£746, £460)
Outcomes					
QALYs gained (Hernandez Alava and Pudney)	60	0.1168 (0.1114, 0.1223)	61	0.1246 (0.1203, 0.1288)	-0.0077 (-0.0146, -0.0009)
QALY gained (Van Hout)	60	0.1086 (0.1027, 0.1144)	61	0.1184 (0.1140, 0.1229)	-0.0099 (-0.0172, -0.0026)
Orelud and Molander (primary outcome, Week7-Week2)	93	-55.4 (-59.5, -51.3)	91	-59.0 (-62.3, -55.7)	3.6 (-1.6, 8.8)
Orelud and Molander (Week12-Week2)	78	-41.3 (-45.3, -37.4)	74	-36.1 (-39.5, -32.7)	-5.3 (-10.5, 0.0)

Note: Costs are in 2020/21 prices.

Figure S1 – Distribution of costs: total NHS+PSS, total societal costs, productivity loss and cost of informal care.

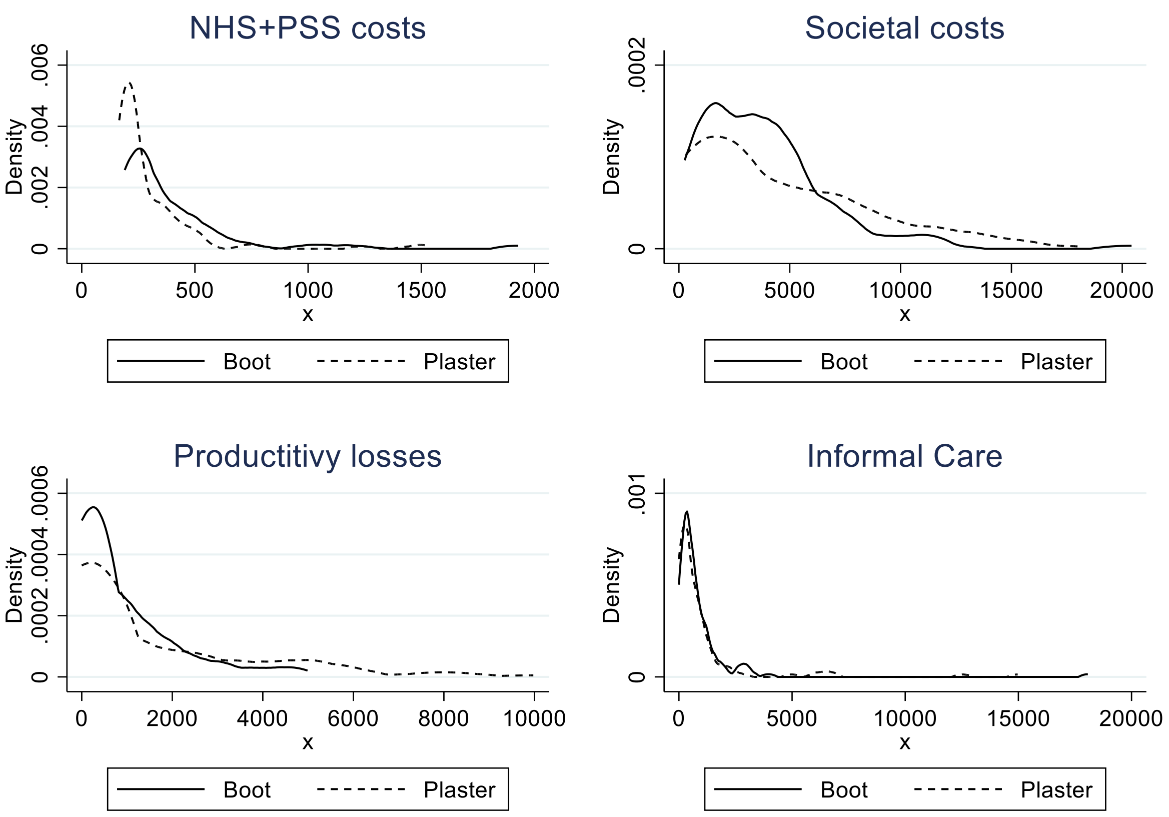

Figure S2 – Distribution of utility scores for QALY gains

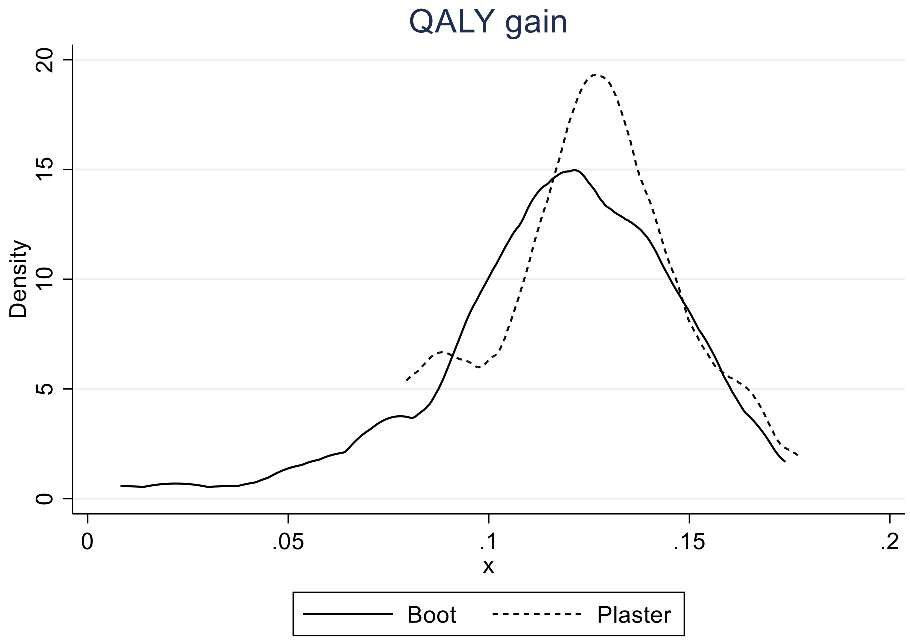

Figure S3 Cost-effectiveness Plane comparing boot (intervention) to cast (control)

a) NHS + PSS perspective

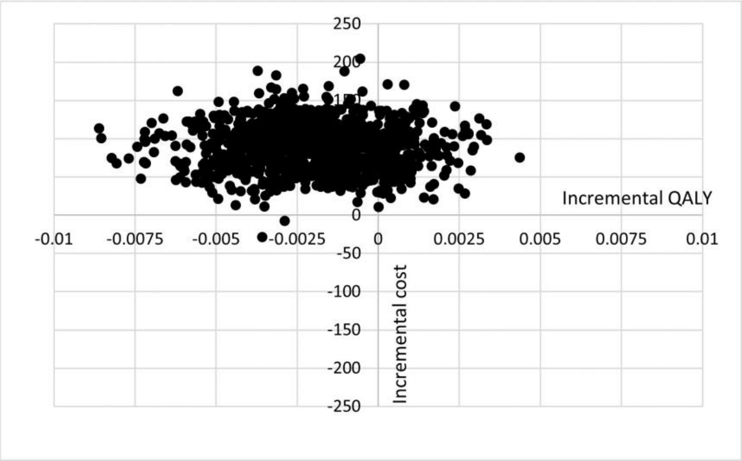

b) Societal perspective

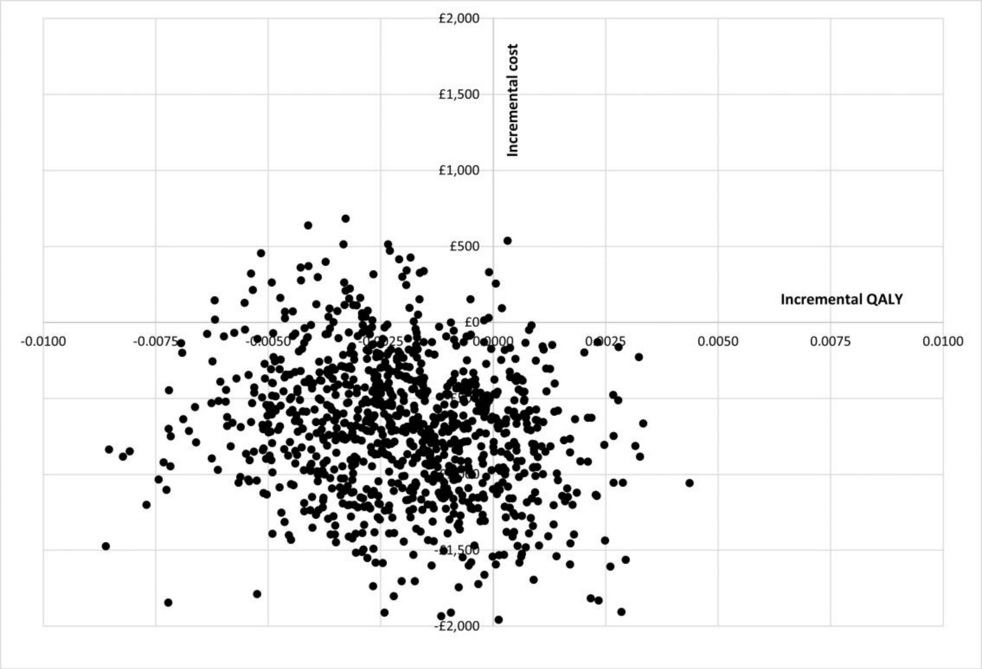

Figure S4 Cost-effectiveness Acceptability Curve comparing boot (intervention) to cast (control)

(a) NHS + PSS perspective

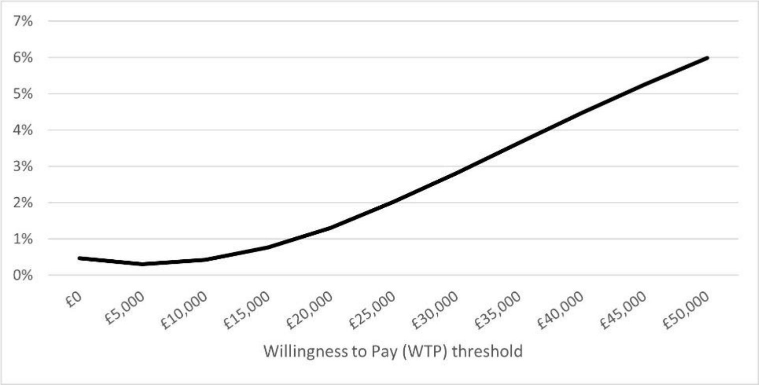

(b) Societal perspective

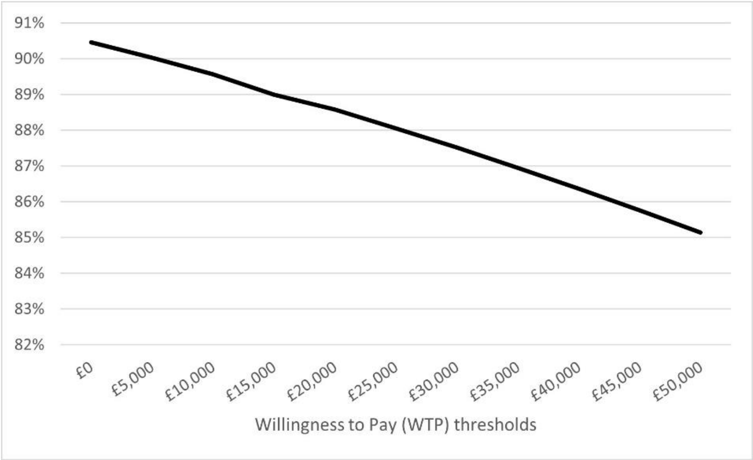

Table S5 One-way sensitivity analysis

**NHS+PSS perspective**<br>**One-way sensitivity analysis**	**Scenario 1**<br>**25% reduction in boots price**<br>Mean (95%CI)	**Scenario 2**<br>**50% reduction in boots price**<br>Mean (95%CI)	**Scenario 3**<br>**75% reduction in boots price**<br>Mean (95%CI)
Incremental Cost	£76 (£9 to £142)	£63 (-£3 to £130)	£51 (-£16 to £117)
Incremental QALY	-0.0020 (-0.0067 to 0.0026)	-0.0020 (-0.0067 to 0.0026)	-0.0020 (-0.0067 to 0.0026)
Net Monetary Benefit	-£117 (-£218 to -£15)	-£104 (-£206 to -£3)	-£92 (-£193 to £10)
Probability of Cost-effectiveness	2.2%	3.6%	5.7%
