# Supplementary material for: Use of removable support boot versus cast for early mobilisation after ankle fracture surgery: cost-effectiveness analysis and qualitative findings of the Ankle Recovery Trial (ART)
Source: BMJ Open. 2024 Jan 12;14(1):e073542. doi: 10.1136/bmjopen-2023-073542 (PMC10810042; doi:10.1136/bmjopen-2023-073542)
Supplement: Author's manuscript [file bmjopen-2023-073542.draft_revisions.pdf]

BMJ Open

BMJ Open is committed to open peer review. As part of this commitment we make the peer review history of every article we publish publicly available.

When an article is published we post the peer reviewers' comments and the authors' responses online. We also post the versions of the paper that were used during peer review. These are the versions that the peer review comments apply to.

The versions of the paper that follow are the versions that were submitted during the peer review process. They are not the versions of record or the final published versions. They should not be cited or distributed as the published version of this manuscript.

BMJ Open is an open access journal and the full, final, typeset and author-corrected version of record of the manuscript is available on our site with no access controls, subscription charges or pay-per-view fees (http://bmjopen.bmj.com).

If you have any questions on BMJ Open's open peer review process please email
info.bmjopen@bmj.com

BMJ Open

Use of removable support boot vs cast for early mobilisation after ankle fracture surgery: Cost-effectiveness analysis and qualitative findings of the Ankle Recovery Trial. (ART)

Journal:	*BMJ Open*
Manuscript ID	bmjopen-2023-073542
Article Type:	Original research
Date Submitted by the Author:	08-Mar-2023

Complete List of Authors:	Baji, Petra; University of Bristol; Corvinus University of Budapest,<br>Barbosa, Estela; City University of London; UKPRP VISION Consortium<br>Heaslip, Vanessa; University of Salford, Directorate of Nursing and Midwifery; University of Stavanger<br>Sangar, Bob; University Hospitals Dorset NHS Foundation Trust<br>Tbaily, Lee; University Hospitals Dorset NHS Foundation Trust<br>Martin, Rachel; University Hospitals Dorset NHS Foundation Trust<br>Docherty, Sharon ; Bournemouth University, Department of Medical Science and Public Health<br>Allen, Helen; Bournemouth University, Bournemouth University Clinical Research Unit<br>Marques, Elsa; University of Bristol, Bristol Medical School; University of Bristol, NIHR Biomedical Research Centre<br>Hayward, Christopher; Plymouth University, Peninsula Clinical Trials Unit

Keywords:	Foot & ankle < ORTHOPAEDIC & TRAUMA SURGERY, Clinical Trial, HEALTH ECONOMICS

SCHOLARONE™
Manuscripts

I, the Submitting Author has the right to grant and does grant on behalf of all authors of the Work (as defined in the below author licence), an exclusive licence and/or a non-exclusive licence for contributions from authors who are: i) UK Crown employees; ii) where BMJ has agreed a CC-BY licence shall apply, and/or iii) in accordance with the terms applicable for US Federal Government officers or employees acting as part of their official duties; on a worldwide, perpetual, irrevocable, royalty-free basis to BMJ Publishing Group Ltd ("BMJ") its licensees and where the relevant Journal is co-owned by BMJ to the co-owners of the Journal, to publish the Work in this journal and any other BMJ products and to exploit all rights, as set out in our licence.

The Submitting Author accepts and understands that any supply made under these terms is made by BMJ to the Submitting Author unless you are acting as an employee on behalf of your employer or a postgraduate student of an affiliated institution which is paying any applicable article publishing charge ("APC") for Open Access articles. Where the Submitting Author wishes to make the Work available on an Open Access basis (and intends to pay the relevant APC), the terms of reuse of such Open Access shall be governed by a Creative Commons licence – details of these licences and which Creative Commons licence will apply to this Work are set out in our licence referred to above.

Other than as permitted in any relevant BMJ Author's Self Archiving Policies, I confirm this Work has not been accepted for publication elsewhere, is not being considered for publication elsewhere and does not duplicate material already published. I confirm all authors consent to publication of this Work and authorise the granting of this licence.

For peer review only

Title **Use of removable support boot vs cast for early mobilisation after ankle fracture surgery: Cost-effectiveness analysis and qualitative findings of the Ankle Recovery Trial. (ART)**

Petra Baji* (Corresponding author)
Musculoskeletal Research Unit, Bristol Medical School, Translational Health Sciences, University of Bristol, BS10 5NB Bristol, United Kingdom
Corvinus University of Budapest, Budapest, Hungary
Email: petra.baji@bristol.ac.uk

Estela C. Barbosa*
City University of London, Northampton Square, London EC1V 0HB, United Kingdom
UKPRP VISION Consortium, London, UK
Email: estela.barbosa@city.ac.uk

Vanessa Heaslip
Directorate of Nursing and Midwifery, University of Salford, United Kingdom
Department of Social Work, University of Stavanger, Norway

Anuraag Sangar
University Hospitals Dorset NHS Foundation Trust, United Kingdom

Lee Tbaily
University Hospitals Dorset NHS Foundation Trust, United Kingdom

Rachel Martin
University Hospitals Dorset NHS Foundation Trust, United Kingdom

Sharon Docherty
Department of Medical Science and Public Health, Bournemouth University, United Kingdom

Helen Allen
Bournemouth University Clinical Research Unit, Bournemouth University, United Kingdom

For peer review only - http://bmjopen.bmj.com/site/about/guidelines.xhtml

Christopher Hayward Peninsula Clinical Trials Unit, University of Plymouth, United Kingdom

Elsa Marques Musculoskeletal Research Unit, Bristol Medical School, Translational Health Sciences
NIHR Biomedical Research Centre, University of Bristol, Bristol
United Kingdom
e.marques@bristol.ac.uk

* shared first authorship

Key words Ankle fracture surgery, early mobilisation, plaster, support boot, RCT, cost-effectiveness.

Word count 3867

(excluding title page, abstract, tables and declarations and references)

For peer review only

Abstract (299/300)

Objectives: To estimate the cost-effectiveness of using a removable boot with a cast following ankle fractures, from the National Health Service and Personal Social Services (NHS+PSS) payer and the societal perspectives; and explore the impact of both treatments on participants' activities of daily living.

Design: Cost-effectiveness analyses and qualitative interviews performed alongside a pragmatic multi-centre randomised controlled trial

Setting: Eight UK NHS secondary care trusts.

Participants: 243 participants (60.5% female, on average 48.2 years of age [standard deviation 16.4]) with ankle fracture. Qualitative interviews with sixteen trial participants.

Interventions: removable air boot with additional exercises (intervention) vs plaster cast and exercise leaflet (control) 2-weeks after surgery.

Primary and secondary outcome measures: quality-adjusted life years (QALYs) estimated from the EQ-5D-5L questionnaire, costs, and incremental net monetary benefit statistics (INMB) measured 12-weeks after surgery, for a society willing-to-pay £20,000 per QALY.

Results: Care in the boot group costed, on average, £88 (95% CI £22 to £155) per patient more than in the plaster group from the NHS+PSS perspective. When including all societal costs, the boot saved, on average, £676 per patient (95% CI -£337 to £1,689). Although there was no evidence of a QALY difference between the groups (-0.0020 [95% CI -0.0067 to 0.0026]), the qualitative findings suggest participants felt the boot enhanced their quality of life. Patients in the boot felt more independent and empowered to take on family responsibilities and social activities.

Conclusions: While the removable boot is slightly more expensive than plaster cast for the NHS+PSS payer at 12-weeks after surgery, it reduces productivity losses and the need for informal care and empowers patients. Given that differences in QALYs and costs to the NHS are small, the decision to use a boot or plaster following ankle surgery could be left to patients' and clinicians' preferences.

Trial Registration: ISRCTN15497399

Key words: Ankle fracture surgery, early mobilisation, plaster, support boot, RCT, cost-effectiveness.

For peer review only - http://bmjopen.bmj.com/site/about/guidelines.xhtml

Article Summary

Strengths and limitations of this study

- *We performed a full economic evaluation alongside a definitive multi-centre randomized controlled trial in the UK comparing a removable boot and exercise with a plaster cast for treatment of ankle fractures two-weeks post-surgery.*
- *Our study collected resource use data from 243 trial participants and a review of medical notes as the trial centres to estimate costs from both the National Health Service and Personal Social Services (NHS+PSS) payer and the societal perspectives.*
- *The study was strengthened by a nested qualitative study to explore the impact of both treatments on participants' activities of daily living.*
- *The RCT was not powered to detect a difference in quality-of-life or costs and there is uncertainty in the economic result, with the confidence interval around the incremental net monetary benefit statistics crossing the null.*

For peer review only

For peer review only - http://bmjopen.bmj.com/site/about/guidelines.xhtml

Background

Every year there are about 20,000 people in England admitted for ankle fractures [1]. Ankle fractures affect people of all ages, most of working age [2], and have been associated with long-term disability and pain, resulting in time-off work and other economic consequences [3] [4]. A previous meta-analysis suggested that the early return to function should be the main goal following surgical management of fractures [5].

After surgery, patients are typically immobilised with a plaster cast. After a couple of weeks, ankle management options are more varied. Another plaster cast can be applied for several more weeks after surgery, which allows weight bearing and maximizes support, but has been associated with joint stiffness, muscle atrophy, and deep vein thrombosis [6-8]. One alternative is to use a removable air boot. The boot can be removed to mobilise the ankle, to exercise, and for comfort such as for taking a bath, but could increase the risk of complications. The Ankle Recovery Trial (ART) compared these two alternative ankle fracture management treatments from two weeks post-surgery until 12-weeks post-surgery and included a full economic evaluation and nested qualitative study. The aim of this paper is to report the results of the cost-effectiveness and qualitative analyses conducted alongside the ART trial to further the evidence base and inform future national guidance on post-surgical ankle management.

For peer review only

Methods

Trial design and ethics approval

The ART trial was a pragmatic multi-centre RCT conducted across eight UK NHS secondary care trusts between July 2015 and November 2018. ART included a qualitative component and health economic evaluation. The trial compared casts with removable support boots as methods of ankle fracture management from two weeks post-surgery until 12-weeks post-surgery, for a period of 10 weeks. Participants completed questionnaires at baseline/randomization (2 weeks after surgery), and then at 6-, 7-, and 12-weeks post-surgery. Most participants at the 6-week follow-up timepoint (6-weeks post-surgery routine appointment with the physiotherapist) were expected to remove their plaster cast or boot and

For peer review only - http://bmjopen.bmj.com/site/about/guidelines.xhtml

start to mobilise freely. The primary clinical endpoint was the Olerud and Molander ankle score (OMAS) [9] measured at 7-weeks post-surgery, when participants should have adapted to mobilise without plaster cast or boot. The ART trial was approved by the South Central – Hampshire A Research Ethics Committee (reference 14/SC/1409). The clinical effectiveness of intervention is reported elsewhere.

Patient and Public Involvement

Nine patient advisors, male and female, most of whom had experienced an ankle fracture, advised on several aspects of the trial, including on patient facing materials and on the interview schedule. Two advisors also alternated attending the trial steering committee.

Participants

The economic and qualitative study collect data on participants in the ART trial, 2 weeks after receiving open reduction and surgical internal fixation for an unstable ankle fracture.

Intervention and usual care

Participants were randomly allocated to receive either a removable air boot and an exercise booklet (intervention) or a plaster cast (control) at the two-weeks post-surgery appointment with the research physiotherapist. The intervention group were fitted with the air boot and given additional exercises to encourage early mobilisation. Removable support boots could be any make or model that allowed at least two air cells to provide compression, had rigid (e.g. plastic) anterior and posterior outer sections, were suitable for weight-bearing, and removable to allow ankle mobilisation. Casts were standard below-knee rigid shells with soft lining which supported the ankle in plantigrade and were provided with a plaster shoe to allow weight-bearing. All participants were provided a group-specific exercise leaflet and exercises were taught by a research physiotherapist. Participants were encouraged to mobilise their ankle by completing exercises as often as pain allowed, advised on gait re-education with crutches and to progress to weight-bearing as able. .

Overview of economic evaluation

We conducted a cost–utility analysis and a cost-effectiveness analysis, comparing the boot with the cast group at 12-weeks after ankle stabilisation surgery. The primary analysis took a

For peer review only - http://bmjopen.bmj.com/site/about/guidelines.xhtml

National Health Service plus Personal Social Services (NHS+PSS) perspective. The secondary analysis took a societal perspective on costs, which included private expenses, informal care, and productivity losses. All analyses followed the pre-specified health economics analysis plan [10] and were performed in STATA version 17.

Resource use data collection for the economic evaluation

At 2-weeks after surgery, the physiotherapist and/or plaster technicians recorded staff grade, time, and resources required to fit the boot or the plaster, including brand of boot. Research nurses/physiotherapists reviewed medical notes at trial centres to collect data on further visits to hospital within the study 10-week period. Resources used within the first 2 weeks of surgery were not collected, as randomisation took place 2 weeks after surgery. Other resource use was collected from participants in postal questionnaires at 6- and 12- weeks follow-up points. Questionnaires included questions about physiotherapy received in hospital or in the community; general practice (GP) contacts with doctors and nurses; additional outpatient appointments or A&E attendances and admissions to hospitals outside the trial centres; NHS equipment (e.g., walking aids); prescribed medications; contacts with social worker, home care help services, and food-at-home services; privately paid physiotherapy, medical equipment, and home care or food services; productivity losses measured in terms of time-off work (absenteeism) and unproductive time at work (presenteeism); and time spent on informal care by a friend or relative.

Outcome data collection for the economic evaluation

Outcome data were collected at baseline, 6-, 7-, and 12-weeks post-surgery. We used responses to the EQ-5D-5L questionnaire to compute a utility score to estimate quality-adjusted life years (QALYs) [11]. The EQ-5D-5L is a patient-reported outcome instrument, standardised and validated to measure generic health-related quality of life [11]. It includes five dimensions: mobility, self-care, usual activities, pain/discomfort, and anxiety/depression. It is widely used in economic analysis in clinical trials as it allows for the direct comparison of health benefits across different clinical interventions.

OMAS was the primary clinical outcome of the trial. The OMAS is patient-reported outcome measure with scores varying between 1 and 100. A clinically meaningful difference in OMAS was defined as 10-points or more[12].

For peer review only - http://bmjopen.bmj.com/site/about/guidelines.xhtml

Valuing resource use to derive costs

We used a micro-costing approach to estimate the cost of delivering treatment in both arms. We used local procurement prices for the removable boots and plaster materials, and valued staff grades and time spent delivering both treatments using Unit Costs of Health and Social Care (PSSRU) [13]. Secondary care resources were valued using the UK NHS National Collection of Costs [14] and the British National Formulary for medications [15]. Productivity losses were valued using a human capital approach and the ONS averaged gross earning per hour (2021) for the relevant age group of the trial participants (age 40-49) [16], and the minimum wage rates between April 2021 to March 2022 for informal care [17]. Unit costs applied and their sources are presented in the Appendix Table S1. Costs were calculated by multiplying the units of resource used by its unit cost.

Valuing EQ-5D-5L to derive QALYs

EQ-5D-5L utility scores at baseline, 6,7 and 12-weeks post-operative were mapped from the 3L version using Hernandez Alava and Pudney`s algorithm [18] Utility scores are bound at a maximum of 1 (corresponding to perfect health), where 0 corresponds to death. Negative values were permitted for health states worse than death. We calculated accumulated QALY gains per patient using the area-under-the-curve approach, assuming a linear change between utility scores at each time point[19]. For comparison, we also produced utility scores using Van Hout's algorithm, which was recommended mapping algorithm by NICE before 2022 [20].

Cost-Utility Analysis

Analyses were intention-to-treat, where participants' data were analysed based on their randomly allocated treatment group.

We imputed missing costs, utility scores, and Olerud and Molander ankle scores (OMAS) at each time point using multiple imputation models [21] with chained equations, with 50 sets and predictive mean matching, assuming that data were not missing completely at random (details of imputation model in Appendix Imputation of missing data). Costs and outcomes were then adjusted for hospital site (trial stratification variable) and pre-specified variables (age, sex, and fracture complexity) [10]. QALYs and OMAS were further adjusted for baseline scores [19].

For peer review only - http://bmjopen.bmj.com/site/about/guidelines.xhtml

We plotted costs and outcomes distributions to explore differences between the groups and cost drivers.

We jointly estimated the difference between groups in terms of costs and outcomes using Seemingly Unrelated Regressions (SUR) and computed the correlation of residuals. .

We estimated cost-effectiveness in relation to QALYs from the NHS+PSS and societal perspectives using the Incremental Net Monetary Benefit (INMB) with a willingness-to-pay (WTP) threshold of £20,000 per QALY [22]. We plotted the results of 1,000 bootstrapped iterations of the incremental costs and incremental effects in cost-effectiveness planes (CEP) and the corresponding cost-effectiveness acceptability curves (CEAC) to illustrate the uncertainty surrounding the decision to adopt the intervention.

Uncertainty and sensitivity analyses

We conducted a complete case analysis to illustrate the impact of missing data and the imputation model in results. To account for model uncertainty we adjusted costs and QALYs also including relationship status, alone living status, employment status, education, and body mass index. We carried out one-way sensitivity analysis for scenarios where we decreased the price of boots by 25%, 50% and 75% to explore the impact of lower prices of boots on the results.

Overview of the qualitative study

Participants were invited to participate in a qualitative interview towards the end of their study period, at approximately 12 weeks post-surgery. Two qualitative researchers chose individuals from the pool of positive responders, ensuring a balance of treatment options, sex, age, hospital site, and baseline OMAS scores. Participants were invited to a 45-60 minute telephone semi-structured interview; a date and time to suit the participants was set for those who agreed to participate. Data were audio recorded and transcribed verbatim. During the interviews, participants were asked to discuss their experiences of living with the treatment (boot or cast) as well as exploring the impact this had on their activities of daily living (personal hygiene, sleep, mobility, work, diet), psychological implications (mood and independence) as well as wider impact on their family and social life (changes to routines, caring responsibilities, finances).

For peer review only - http://bmjopen.bmj.com/site/about/guidelines.xhtml

Analysis of qualitative data

Qualitative interviews were analysed using the six-step process of thematic analysis by Braun and Clarke [23]. We read transcripts to gain a sense of the whole before step-by-step analysis of each transcript to identify codes, categories, and initial themes. Once all interviews were analysed individually, a cross case analysis was undertaken, identifying themes across the whole data set. These themes and the process of analysis were shared with a second qualitative researcher to ensure credibility of the analytical process.

Results

Sample

243 participants were randomised between August 2015 and September 2018 (n=123 boot group and n=120 plaster group). Table 1 describes participant demographics and socio-economic characteristics by arm. No major differences were observed between trial arms for all variables.

Sixteen trial participants recruited across the 8 study sites consented to be interviewed; 6 were males and 10 were females, between the ages of 24 and 77 years; 7 received a plaster cast and 9 received a boot. Most ankle fractures were simple (n=14), whilst two were comminuted (bone is broken into more than two pieces) and there was a range of baseline OMAS scores between 5 and 80.

Costs and outcomes

Table 2 reports costs and outcomes by trial arm including imputed data. Units of resource use per arm is presented in the Appendix, Table S2 and all available cost data (complete cost categories only) are presented in Appendix Table S3. The ankle management treatment cost difference reflects the difference in price between the boot (£50) and plaster (£25), and the difference in staff grade and time fitting each. On average, it took the technicians 25.3 minutes to fit the cast (SD 17.1) and 23.7 minutes to fit the boot (SD 24.9).

For peer review only

NHS+PSS cost differences are small and in favour the plaster group. From a societal perspective, the differences are larger, particularly in relation to productivity loss and informal care and favour the boot group (Figures S1 and S2 of the Appendix).

Cost-effectiveness base case and sensitivity analyses

Table 3 presents the cost-effectiveness analysis results from an NHS+PSS perspective and a societal perspective for the base case and sensitivity analyses. From an NHS+PSS perspective, treatment in the boot group costs, on average, £88 (95% CI £22, £155) per patient more than in the plaster group. From a societal perspective, the boot saved, on average, £676 per patient compared with plaster [95% CI -1,689, £337]). The cost savings for the boot are driven by reduced informal care and time-off productive work. There was no evidence of a QALY difference between the arms (mean QALY gain -0.0020 (95% CI -0.0067, 0.0026).

From an NHS+PSS perspective the boot is dominated, with a negative INMB statistic (mean -£129 [95% CI -£231, -£28)]). When including additional privately incurred costs, productivity losses and the burden of informal care in a societal perspective, the boot is cost-effective when willing-to-pay £20,000 per QALY, with a mean INMB £634, 95% CI [-£266, £1,535]). The societal perspective results, although deriving larger net benefits, are also more uncertain, with wider confidence intervals that cross the null.

The cost-effectiveness plane for the NHS+PSS perspective shows a high concentration of results in the upper left quadrant (Figure 1) reflecting a dominated treatment option. This corresponds to a low probability of the boot being cost-effective even at high willingness-to-pay thresholds in the cost-effectiveness acceptability curve (Figure S3). From a societal perspective (Figure 2) shows a high concentration of results in the lower-left quadrant, but a higher likelihood of being the cost-effective.

These findings are robust in sensitivity analyses to the model specification. Our findings are sensitive to the imputation model. In a complete case analysis, the societal perspective results are closer to those of the NHS+PSS results, with the boot group reporting, on average, higher costs than the cast group. When boots prices decreased by 25%, 50% and 75% in sensitivity analyses, the NHS+PSS cost differences between arms decreased to £76 (95% CI £9, £142),

For peer review only - http://bmjopen.bmj.com/site/about/guidelines.xhtml

£63 (95% CI -£3, £130) and £51 (95% CI -£16, £117), respectively, indicating no evidence of a differences in costs, QALYs, or INMB statistics (Appendix Table S4).

Qualitative findings

The economic results of lower productivity losses and informal care costs in the boot group were reflected in the qualitative interviews. Having a fracture irrespective of the treatment offered (boot or cast) increased reliance on others and this was typically managed by family members and friends which was frustrating for participants, many of whom expressed feeling helpless:

> *"I had depression before and I think that didn't help that I've got that tendency as well…there was some sense of isolation more to do with not being able to see people a lot of the time or not having the same routine. The other thing I found really challenging was having to ask for help*" (Female 44 cast)

> *"It had a huge impact on my independence because it meant I was relying on other people to even leave the house"* (Female 52 cast)

Those with the boot expressed an ability to become either more self-caring or take back family responsibilities earlier, such as walking to the kitchen to prepare dinner and making their own drinks. These activities were harder for those in cast due to immobility.

> *"There's only so much you want to be waited on, everyone says I dream of that but when it comes to it, it actually drives you insane, to be able to get back and cook meals for my children and to be able to go and make myself a cup of tea and then carry it to another room is fantastic* (Female 46 boot)

> *"…for me to be reliant on somebody else, it was degrading…and I felt vulnerable like in a really vulnerable state definitely …I found* [with boot] *I was less dependent on other people because I could move around a bit more, I could have a bath or shower myself, I could get in and out of bed on my own"* (Female 27 boot)

For peer review only

For peer review only - http://bmjopen.bmj.com/site/about/guidelines.xhtml

"It put a huge burden on my daughter who was having to come 4 times a day, but then [with boot] I could go into the kitchen for a large degree to, make a meal for myself you know" (Female 77 boot)

It was also apparent that having a boot enabled participants to continue their wider social connections; they travelled further and continued with wider social engagements (outside the home) than those with a cast. In contrast, those with a cast treatment talked about having to miss family engagements and wider social activities where people with the boot tended to continue to attend these, even if it required hiring additional support, such as a wheelchair.

"Basically, I was stuck around the house, we had to cancel our holiday… to be honest I just sat round the house pretty much for six weeks" (Male 69 cast)

"there were family BBQs where basically my husband took the kids and I just relaxed at home, it was a bit too much" (Female 44 cast)

"I feel the boot is a very good protection around my leg and ankle particularly if I was out and I was using the wheelchair quite a lot as we have a busy social life… I think would have been more twitchy had I had a plaster" (Female 69 boot)

"We hired a wheelchair for me because we went to the MTV Crashes with the kids in Plymouth and that was a whole day come night-time thing" (Female 46 boot)

Patients in the cast group appeared to have increased personal costs purchasing additional clothing and equipment to accommodate the use of the cast.

"I obviously had to buy a waterproof cover for the leg…. I went out and brought a couple of pairs of cropped leggings…I was obviously very limited what I could wear because of the plaster, probably spent about £30 on the cover and leggings" (Female 52 cast)

"I could still wear leggings, I didn't have to cut up any trousers" (Female 27 boot)

For peer review only - http://bmjopen.bmj.com/site/about/guidelines.xhtml

> *"I bought one of those like big hot water bottle kind of thing that went up your leg for showers and things which costs about £14"* (Female 49 cast)

> *"I had to cut up 4 pairs of my leggings up because my leggings wouldn't go over the plaster"* (Female 54 cast)

Patients in the cast group expressed difficulty with stair climbing, while those with boots who described being able to weight bear did not express similar difficulties. Another advantage identified by participants in boots was the ability to remove it to undertake the prescribed exercises, which they felt aided a quicker recovery. Of the five participants who expressed a preference, two in cast would have preferred boots and the other three were glad they had received boots.

> *"…because the foot got really swollen* [from toes to my knee]*, so the advantage of taking the boot off and putting peas on it, in your own mind you were doing something to ease the cause"* (Female 53 boot)

> *"One of the main advantages was I was able to start my physio a lot earlier… I was able to practice flexing and getting my foot up and turning it. The flexibility in my foot its better, it's completely back to normal now where I think if it was in a cast… everything would have started to seize up a lot more and would have been harder to get the flexibility back"* (Female 46 boot).

In the qualitative interviews, participants reported that they felt the boot enhanced their quality of life.

> *"I found the boot easier than the plaster. The plaster was very heavy and I found with the boot I could actually weight bear a bit more and get on with day to day running of life"* (Female 53 boot)

> *"I wanted the boot because I knew the boot would be better for me personally… because I am so active I wanted to get around and do things"* (Male 24 boot)

For peer review only - http://bmjopen.bmj.com/site/about/guidelines.xhtml

Discussion

Statement of principal findings

The found that the boot group had, on average, higher NHS+PSS costs but differences between groups were small. Differences in QALY gains between groups were negligible. When including costs with informal care burden and productivity losses from time-off or unproductive work, the boot saves society on average £676, and could be a cost-effective intervention when willing-to-pay £20,000 per QALY. Results from our qualitative interviews show that participants in the boot group were more satisfied with their treatment option, felt more independent, and were able to return to their usual activities sooner.

Strengths and limitations

Our study reports a full economic evaluation from and NHS+PSS perspective and a nested qualitative study conducted alongside a large pragmatic multicentre RCT in the management of ankle factures after surgery. The nested qualitative study shed further light to the interpretation of economic findings. The ART trial is the largest RCT to date comparing removable support boot with a plaster cast for early mobilisation after ankle fracture surgery and ours is the first cost-effectiveness evaluation comparing the two ankle management treatment options.

The ART trial was not powered to detect a difference in costs, QALYs, or INMB statistics, and the results were uncertain. Missing data for the economic evaluation was relatively high and not completely at random. Our base-case results include imputed data, due to the risk of biases in a complete case analyses, which changed the results for the societal perspective. The larger cost driver in favour of the boot is informal care burden. Measuring the burden for carers in economic evaluation is often neglected and methods to value carer burden are not contemplated in the NICE reference case.[22]

The EQ-5D-5L questionnaire appears to be too insensitive to pick up important aspects of quality of life that the boot can provide to patients. It does not directly measure the broader aspects of wellbeing and ability that were captured in the qualitative interviews. The perception of increased wellbeing in the qualitative interviews, however, may be a result of expectations towards the newer technology, which is difficult to objectively measure.

For peer review only - http://bmjopen.bmj.com/site/about/guidelines.xhtml

Results in context

A recent systematic review of the clinical effectiveness and cost-effectiveness of orthotic walking boots for patients with ankle fractures or ligament injuries, found no previous cost-effectiveness studies comparing boots with casts [24]. The ART trial and economic evaluation found no evidence of a clinically important difference between arms in the OMAS or QALY scores at 7 or 12 weeks post-surgery (clinical results reported elsewhere) and that more participants in the boot arm experienced complications, particularly incidences of wound breakdown. All complications were minor, and the wounds went on to heal without sequelae. Previous evidence from three retrospective non-randomized studies also suggest that boots provided greater clinical effectiveness or comparable to plaster cast, but more skin complications were observed with the use of casts than treatment with boots [24].

Conclusion

While the boot is more expensive than cast for the NHS+PSS payer, it reduces productivity losses and the need for informal care and empowers patients. Given that differences in QALYs and costs to the NHS are small, the decision to use a boot or cast following ankle surgery could be left to patients' and clinicians' preferences.

List of Abbreviations

ART – The Ankle Recovery Trial
CEAC – Cost-effectiveness Acceptability Curve
CEA – Cost-Effectiveness Analysis
CUA – Cost Utility Analysis
INMB – Incremental Net Monetary Benefit
NHS – National Health System
NICE - National Institute for Health Care and Excellence
OMAS – Olerud and Molander Scoring System
PSS – Personal Social Services
QALY – Quality-Adjusted Life Year

For peer review only - http://bmjopen.bmj.com/site/about/guidelines.xhtml
For peer review only

QoL – Quality-of-Life

RCT – Randomised Controlled Trial

SUR - Seemingly Unrelated Regressions

UK – United Kingdom

WTP – Willingness to Pay

For peer review only

For peer review only - http://bmjopen.bmj.com/site/about/guidelines.xhtml

Declarations

Acknowledgements

The views expressed herein are those of the authors and not necessarily those of the NHS, the NIHR, or the Department of Health and Social Care.

The protocol for the study

The protocol for the study is available via:

[Bournemouth University Research Online [BURO] - ART: Ankle Recovery Trial Protocol v 1.6.]

SHEAP is available via:

[Bournemouth University Research Online [BURO] - Statistical and Health Economics Analysis Plan: Ankle Recovery Trial (ART).]

Funding statement

This report is independent research funded by the National Institute for Health and Care Research (NIHR) under its Research for Patient Benefit Programme 'Does early mobilisation after Ankle fracture surgery enhance Recovery? A pragmatic multi-centre randomised controlled Trial with qualitative component and health economic analysis comparing the use of plaster versus support boot (ART)', PB-PG-0213-30021. The views expressed in this publication are those of the author(s) and not necessarily those of the NHS, the National Institute for Health and Care Research or the Department of Health and Social Care.

A competing interests statement

The authors declare that they have no competing interests.

Author contributions

AS, EMRM, CH, HA, conceived the idea for the study and secured funding. EMRM designed the economic evaluation and supervised PB and ECB in performing the economic evaluation. PB, ECB, EMRM had access to data and conducted the economic analysis. VH designed and VH and HA conducted the qualitative study. ECB, PB, EMRM, and VH drafted the manuscript. All authors critically revised the manuscript and approved the final version for submission. PB and ECB contributed equally to this paper as first authors.

For peer review only - http://bmjopen.bmj.com/site/about/guidelines.xhtml

Patient consent form

Informed Consent forms were filled in by each patients participated in the ART trial (ISRCTN15497399). No individual person's data are included in the manuscript.

Data sharing statement

The datasets used and/or analysed during the current study are available from the corresponding author on reasonable request.

Conflict of interest

The authors declare no conflict of interest related to the content of the manuscript.

For peer review only

References

1. Scott LJ, Jones T, Whitehouse MR, et al. Exploring trends in admissions and treatment for ankle fractures: a longitudinal cohort study of routinely collected hospital data in England. *BMC Health Services Research* 2020;20:1-10.
2. Gill JR, Lostis E, Bleibleh SS, et al. Weight-bearing in ankle fractures: An audit of UK practice. *The Foot* 2019;39:28-36.
3. McPhail SM, Dunstan J, Canning J, et al. Life impact of ankle fractures: Qualitative analysis of patient and clinician experiences. *BMC musculoskeletal disorders* 2012;13(1):1-13.
4. Nilsson GM, Jonsson K, Ekdahl CS, et al. Unsatisfactory outcome following surgical intervention of ankle fractures. *Foot and Ankle Surgery* 2005;11(1):11-16.
5. Lin CWC, Donkers NA, Refshauge KM, et al. Rehabilitation for ankle fractures in adults. *Cochrane database of systematic reviews* 2012(11)
6. Shaffer MA, Okereke E, Esterhai Jr JL, et al. Effects of immobilization on plantar-flexion torque, fatigue resistance, and functional ability following an ankle fracture. *Physical Therapy* 2000;80(8):769-80.
7. Stevens JE, Walter GA, Okereke E, et al. Muscle adaptations with immobilization and rehabilitation after ankle fracture. *Medicine and science in sports and exercise* 2004;36(10):1695-701.
8. Vioreanu M, Dudeney S, Hurson B, et al. Early mobilization in a removable cast compared with immobilization in a cast after operative treatment of ankle fractures: a prospective randomized study. *Foot & ankle international* 2007;28(1):13-19.
9. Olerud C, Molander H. A scoring scale for symptom evaluation after ankle fracture. *Archives of orthopaedic and traumatic surgery* 1984;103:190-94.
10. Thomas P, Marques E, Docherty S, et al. Statistical and Health Economics Analysis Plan: ART Ankle Recovery Trial version 1.1. University of Bristol. 28th Nov 2018. Available at: https://research-information.bris.ac.uk/en/publications/statistical-and-health-economics-analysis-plan-art-ankle-recovery Accessed: 26/01/2023 2018.
11. Herdman M, Gudex C, Lloyd A, et al. Development and preliminary testing of the new five-level version of EQ-5D (EQ-5D-5L). *Quality of life research* 2011;20(10):1727-36.
12. Olerud C, Molander H. A scoring scale for symptom evaluation after ankle fracture. *Archives of orthopaedic and traumatic surgery* 1984;103(3):190-94.
13. Jones KC, Burns A. Unit Costs of Health and Social Care 2021. Unit Costs of Health and Social Care . . Kent, UK: Personal Social Services Research Unit 2021:177.
14. England N. NHS England: 2019/20 National Cost Collection Data Publication. National Schedule of NHS Costs 2019/20. First published: 22 June 2021., 2021.
15. British Medical Association and the Royal Pharmaceutical Society. BNF 83 British National Formulary March - September 2022.
16. Statistics OfN. Annual Survey of Hours and Earnings (ASHE) Earnings and hours worked, age group: ASHE Table 6, Table 6.5a Hourly pay - Gross (£) - For all employee jobsa: United Kingdom, 2021. Release date: 26 October 2021., 2021.
17. Gov.uk: Minimum wage rates for 2022. Available: https://www.gov.uk/government/publications/minimum-wage-rates-for-2022. Accessed: 23/06/2022 [
18. Hernández-Alava M, Pudney S. Econometric modelling of multiple self-reports of health states: the switch from EQ-5D-3L to EQ-5D-5L in evaluating drug therapies for rheumatoid arthritis. *Journal of Health Economics* 2017;55:139-52.
19. Manca A, Hawkins N, Sculpher MJ. Estimating mean QALYs in trial-based cost-effectiveness analysis: the importance of controlling for baseline utility. *Health economics* 2005;14(5):487-96.
20. Van Hout B, Janssen M, Feng Y-S, et al. Interim scoring for the EQ-5D-5L: mapping the EQ-5D-5L to EQ-5D-3L value sets. *Value in health* 2012;15(5):708-15.

For peer review only - http://bmjopen.bmj.com/site/about/guidelines.xhtml

21. Faria R, Gomes M, Epstein D, et al. A guide to handling missing data in cost-effectiveness analysis conducted within randomised controlled trials. *Pharmacoeconomics* 2014;32(12):1157-70.
22. National Institute for Health and Care Excellence. NICE health technology evaluations: the manual. Published: 31 January 2022
23. Braun V, Clarke V. Using thematic analysis in psychology. *Qualitative research in psychology* 2006;3(2):77-101.
24. Banerjee S, Ryce A. Orthotic Walking Boots for Patients with Fractures or Ligament Injuries: A Review of Clinical Effectiveness and Cost-Effectiveness. 2019

For peer review only

Tables and Figures

Table 1 - Participants baseline characteristics

		Boot (n = 123)		Cast (n = 120)	
		**Mean**	**SD**	**Mean**	**SD**
Age	n=243	48.75	15.71	47.7	17.1
BMI	n=239	28.29	5.79	27.42	5.54
		**Count**	**%**	**Count**	**%**
Gender	Male	46	37.4%	49	40.8%
	Female	77	62.6%	70	58.3%
	Missing	0	0.0%	1	0.8%
Fracture complexity	Simple	101	82.1%	99	82.5%
	Other	21	17.1%	19	15.8%
	Missing	1	0.8%	2	1.7%
Living alone prior to injury	Yes	96	78.0%	95	79.2%
	No	25	20.3%	18	15.0%
	Missing	2	1.6%	7	5.8%
Marital status	Single	34	27.6%	34	28.3%
	Married	56	45.5%	61	50.8%
	Civil partnership	8	6.5%	9	7.5%
	Divorced/ partnership dissolved	16	13.0%	3	2.5%
	Widowed/surviving civil partner	6	4.9%	6	5.0%
	Missing	3	2.4%	7	5.8%
Education	None	9	7.3%	15	12.5%
	GCSE and A/AS level	49	39.8%	47	39.2%
	First degree	21	17.1%	18	15.0%
	Higher degree	19	15.4%	14	11.7%
	Other	11	8.9%	13	10.8%
	Missing	14	11.4%	13	10.8%
Employment	Full time paid employment	62	50.4%	53	44.2%
	Part time paid employment	15	12.2%	15	12.5%
	Retired	24	19.5%	20	16.7%
	Volunteer	0	0.0%	1	0.8%
	Unemployed	4	3.3%	4	3.3%
	Looking after home	7	5.7%	7	5.8%
	Full time education	2	1.6%	6	5.0%
	Other	7	5.7%	7	5.8%
	Missing	2	1.7%	7	5.8%

For peer review only

Table 2 – Costs and outcomes by trial arm and by perspective on costs (imputed data)

	Boot (N=123)	Cast (N=120)	Difference
NHS+PSS	Mean (95% CI)	Mean (95% CI)	Mean (95% CI)
Ankle treatment (boot or cast)	£203 (£200, £206)	£179 (£177, £181)	£24 (£20, £28)
Physiotherapy in the community	£11 (£4, £18)	£4 (£1, £7)	£7 (-£1, £14)
Physiotherapy in hospital	£26 (£15, £37)	£24 (£12, £36)	£2 (-£14, £18)
GP practice services	£57 (£30, £83)	£37 (£9, £64)	£20 (-£18, £58)
Social Care services	£19 (£8, £30)	£6 (£0, £12)	£13 (£0, £25)
A&E and Outpatient services	£55 (£25, £85)	£31 (£18, £43)	£24 (-£9, £57)
Hospital Admissions	£8 (-£7, £23)	£8 (-£7, £24)	-£0 (-£22, £22)
NHS Equipment	£3 (£1, £5)	£3 (£1, £5)	£0 (-£2, £3)
Medications	£7 (£4, £9)	£5 (£1, £8)	£2 (-£2, £6)
Private costs and productivity losses			
Private physiotherapy	£16 (£5, £27)	£19 (£7, £31)	-£3 (-£19, £13)
Additional medical Equipment	£5 (£2, £9)	£1 (-£0, £2)	£4 (£0, £8)
Private community care	£0 (-£0, £1)	£1 (-£1, £4)	-£1 (-£3, £2)
Other private expenditures on services/activities	£105 (£75, £134)	£139 (£31, £247)	-£34 (-£145, £76)
Other major expenditure not reported elsewhere	£10 (-£1, £20)	£20 (£2, £38)	-£10 (-£31, £10)
Productivity Loss (time-off work and leisure)	£2,359 (£1,912, £2,807)	£2,995 (£2,334, £3,657)	-£636 (-£1,427, £155)
Informal Care	£888 (£567, £1,209)	£1,025 (£630, £1,420)	-£137 (-£642, £367)
Outcomes			
QALYs gained (Hernandez Alava and Pudney)	0.1195 (0.1144, 0.1247)	0.1248 (0.1208, 0.1287)	-0.0052 (-0.0117, 0.0013)
QALYs gained (Van Hout)	0.1117 (0.1062, 0.1172)	0.1189 (0.1146, 0.1231)	-0.0071 (-0.0141, -0.0002)
Olerud and Molander (primary outcome, Week7-Week2 baseline)	-55.0 (-59.1, -51.0)	-59.0 (-62.3, -55.7)	4.0 (-1.2, 9.2)
Olerud and Molander (Week12-Week2 baseline)	-39.7 (-43.7, -35.8)	-35.5 (-39.1, -31.8)	-4.3 (-9.7, 1.1)

For peer review only - http://bmjopen.bmj.com/site/about/guidelines.xhtml

Table 3 – Cost Utility Analysis results

**Cost Utility Analysis**	**NHS+PSS Perspective** Mean (95% CI)	**Societal perspective** Mean (95% CI)
**Base Case**		
Incremental Cost	£88 (£22 to £155)	-£676 (-£1,689 to £337)
Incremental QALY	-0.0020 (-0.0067 to 0.0026)	-0.0021 (-0.0067 to 0.0026)
Net Monetary Benefit	-£129 (-£231 to -£28)	£634 (-£266 to £1,535)
Probability boot is cost-effective at £20,000/QALY	1.3%	88.6%
**Sensitivity Analysis**		
Models type 2 – adjusting for additional covariates		
Incremental Cost	£74 (£8 to £140)	-£1,017 (-£1,967 to -£67)
Incremental QALY	-0.0021 (-0.0067 to 0.0025)	-0.0021 (-0.0067 to 0.0025)
Net Monetary Benefit	-£116 (-£217 to -£15)	£974 (£99 to £1,850)
Probability boot is cost-effective at £20,000/QALY	2.3%	97.5%
Complete Case Analysis		
Incremental Cost	£123 (£19 to £227)	£323 (-£934 to £1,580)
Incremental QALY	-0.0010 (-0.0076 to 0.0056)	-0.0027 (-0.0096 to 0.0041)
Net Monetary Benefit	-£143 (-£327 to £40)	-£378 (-£1,771 to £1,015)
Probability boot is cost-effective at £20,000/QALY	4.1%	28.3%

Models type 1 (base case) adjust for age, gender, fracture complexity and hospital site; Models type 2 also include relationship status (married/partnership vs other), alone living status, employment status (working part or full time vs other), education (higher degree vs other) and BMI;
INMB = incremental net monetary benefit measured at a willingness to pay for a QALY of £20,000. QALY = quality-adjusted life year.

For peer review only

Figure 1 Cost-effectiveness Plane and Cost-effectiveness Acceptability Curve comparing boot (intervention) to cast (control) - NHS + PSS perspective

Figure 2 – Cost-effectiveness Plane and Cost-effectiveness Acceptability Curve comparing boot (intervention) to cast (control) - Societal perspective

For peer review only

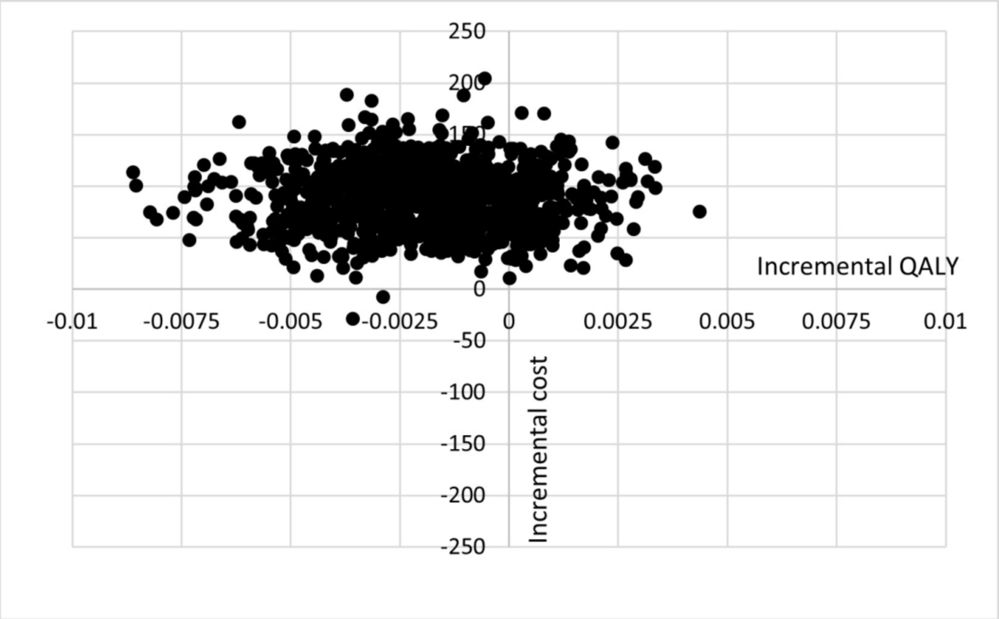

Figure 1 Cost-effectiveness Plane and Cost-effectiveness Acceptability Curve comparing boot (intervention) to cast (control) - NHS + PSS perspective

127x78mm (300 x 300 DPI)

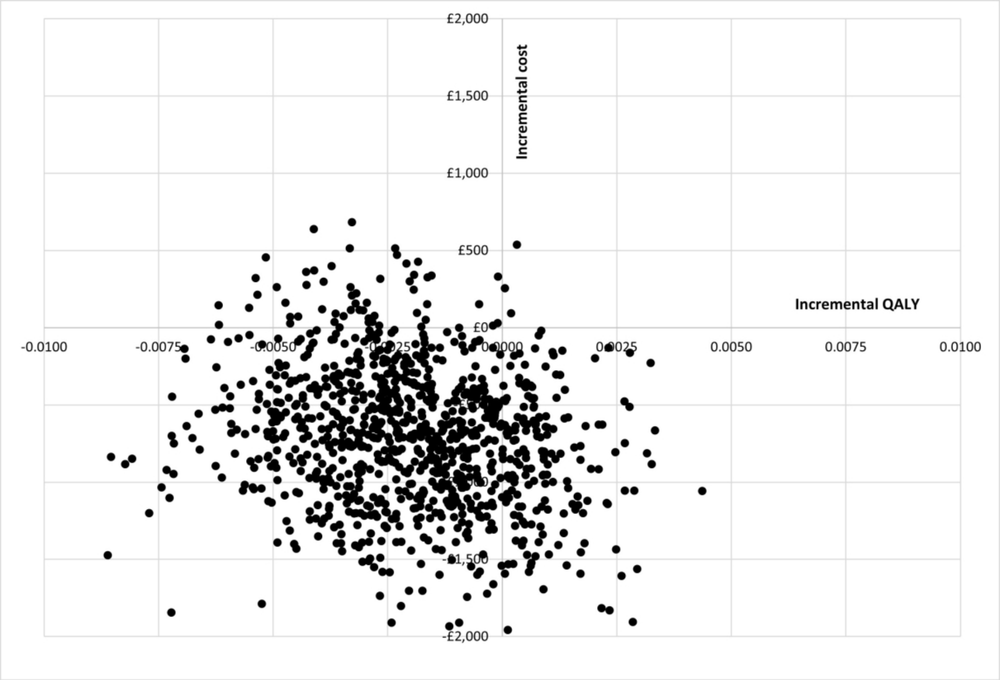

Figure 2 – Cost-effectiveness Plane and Cost-effectiveness Acceptability Curve comparing boot (intervention) to cast (control) - Societal perspective

211x144mm (300 x 300 DPI)

Appendix

Use of removable support boot vs plaster for early mobilisation after ankle fracture surgery: Cost-effectiveness analysis and qualitative findings of the Ankle Recovery Trial. Baji et al.

Imputation of missing data

We imputed missing cost variables, utility scores, and Olerud and Molander ankle scores (OMAS) at each time point using multiple imputation methods (21). We applied chained equations, with 50 sets and predictive mean matching, assuming that data was not missing completely at random. Our imputation model considered treatment arm, hospital site, age, sex, body mass index, relationship status (married/partnership vs other), if the participant lived alone before the injury, education (higher degree vs no higher degree), employment status (working full-time or part-time vs not working) as well as fracture complexity (complex vs not complex), baseline utility, and baseline OMAS scores.

Stata code for imputation

```
mi set wide //register dataset to be imputed
mi register imputed costphysiogp_4 costphysiohospital_4 costphysioprivate_4 /*
 */ costgp_4 costsocialcare_4 costcommunityprivate_4 /*
 */ costhospitalAEoutpatient_4 costhospitaladmission_4 /*
 */ costequipmentprivate_4 costequipmentnhs_4 costotherprivate_4 /*
 */ costmedication_4 costphysiogp_10 costphysiohospital_10 costgp_10 /*
 */ costsocialcare_10 costhospitalAEoutpatient_10 costhospitaladmission_10  /*
 */ costequipmentnhs_10 costequipmentprivate_10 costotherprivate_10  /*
 */  cost_timeoff_4 cost_informalcare_4 cost_majorimpactprivate_4   /*
 */  costmedication_10 costphysioprivate_10  /*
 */   cost_timeoff_10 cost_informalcare_10 cost_majorimpactprivate_10 cost_fitting
cost_intervention //cost variables to be imputed
mi register imputed utility_4 utility_5 utility_10 //QALY variables to be imputed
mi register imputed OreludMolander_w5 OreludMolander_w10 // outcome variables to be
imputed
mi register imputed female complexity utility_baseline OreludMolander_baseline bmi
married_dummy alone work_dummy degree // stratification variables to be imputed
mi register regular treatmentgroup age  site   //regular variables that do not require imputation

mi impute chained (pmm , knn(10)) costphysiogp_4 costphysiohospital_4
costphysioprivate_4 /*
 */ costgp_4 costsocialcare_4 costcommunityprivate_4 /*
 */ costhospitalAEoutpatient_4 costhospitaladmission_4 /*
 */ costequipmentprivate_4 costequipmentnhs_4 costotherprivate_4 /*
 */ costmedication_4 costphysiogp_10 costphysiohospital_10 costgp_10 /*
 */ costsocialcare_10 costhospitalAEoutpatient_10 costhospitaladmission_10  /*
```

For peer review only

```
*/ costequipmentnhs_10 costequipmentprivate_10 costotherprivate_10  /*
*/  cost_timeoff_4 cost_informalcare_4 cost_majorimpactprivate_4   /*
*/  costmedication_10 costphysioprivate_10  /*
*/   cost_timeoff_10 cost_informalcare_10 cost_majorimpactprivate_10 /*
*/ cost_fitting cost_intervention utility_4 utility_5 utility_10 /*
*/ OreludMolander_w5 OreludMolander_w10  /*
*/  female complexity utility_baseline OreludMolander_baseline bmi married_dummy alone
work_dummy degree= treatmentgroup age  site , add(50) by(treatmentgroup) rseed(10) force
```

For peer review only

Table S1. Unit costs

Item	Cost (£)	Source
**Physiotherapy services**		Unit Costs of Health and Social Care 2020/21 (PSSRU)
Provided by GP surgery/practice – at the surgery/practic	40.5	Community-Based Scientific and Professional Staff, cost per hour, Band 5, physiotherapist, occupational therapist, speech and language therapist, podiatrist, clinical psychology assistant practitioner (higher level) counsellor (entry level), 1 hour
Provided by GP surgery/practice – at home	41.5	Community-Based Scientific and Professional Staff, cost per hour, Band 5, physiotherapist, occupational therapist, speech and language therapist, podiatrist, clinical psychology assistant practitioner (higher level) counsellor (entry level), 1 hour
Provided by a hospital – at home	39.5	Hospital based scientific and professional, cost per hour, Band 5, Physiotherapist, Occupational therapist, Speech and language therapist, Podiatrist, Clinical psychology assistant practitioner (higher level), Counsellor (entry level), 1 hour
Provided by a private clinic - at clinic	51.8	Hospital based scientific and professional, cost per hour, Band 6, Physiotherapist, Occupational therapist, Speech and language therapist, Podiatrist, Clinical psychology assistant practitioner (higher level), Counsellor (entry level), 1 hour
Consultations with an Occupational Therapist	88.4	Community-Based Scientific and Professional Staff, cost per hour Band 8.b., Physiotherapist consultant, Occupational therapist consultant, Clinical psychologist principal, Speech and language therapist principal, Podiatric consultant (surgery), Arts therapis t principal, 1 hour
**Community-based Services**		Unit Costs of Health and Social Care 2020/21 (PSSRU)
GP at the surgery/practice	33.0	Primary care practice professionals, Cost per surgery lasting 9.22 minutes (including direct care)
GP at home	132.0	Primary care practice professionals, GP Cost per hour (including direct care)
GP telephone contact	33.0	Primary care practice professionals, Cost per surgery lasting 9.22 minutes (including direct care)
Practice Nurse / Health Care Assistant at GP surgery	42.1	Primary care practice professionals, GP Practice Nurse (cost/hour)
Practice Nurse telephone contact	42.1	Primary care practice professionals, GP Practice Nurse (cost/hour)
Nurse at home	42.1	Primary care practice professionals, GP Practice Nurse (cost/hour)
Health Visitor	34.9	Community-based scientific and professional staff, cost per hour, Band 4

For peer review only

Home care/Home help	23.1	Social care staff, cost per hour, Home Care Worker
Food at home services (e.g. meals on wheels)	24.6	Social care staff, cost per hour, Support and outreach worker
Voluntary Agency Worker/Contact (e.g. from Age UK)	24.6	Social care staff, cost per hour, Support and outreach worker
**Hospital Services**		
Accident and Emergency	187.8	2019-20_National_schedule_of_NHS_costs_FY19_20_V2, A&E visits weighted average, inflated to 2020/21 prices using NHSCII Pay & Prices
Day case admission or procedure (but did not spend the night)	838.0	2019-20_National_schedule_of_NHS_costs_FY19_20_V2, Day cases weighted average, inflated to 2020/21 prices using NHSCII Pay & Prices, Assuming there is a procedure
Outpatient appointment	136.9	2019-20_National_schedule_of_NHS_costs_FY19_20_V2, OP appointments weighted average, inflated to 2020/21 prices using NHSCII Pay & Prices
**Time off work (productivity loss)**	20.4	Gross earning per hour, 2021, Earnings and hours worked, age group: ASHE Table 6 - Office for National Statistics (ons.gov.uk), Table 6.5a Hourly pay - Gross (£) - For all employee jobsa: United Kingdom, 2021, Age group 40-49
**Support from others (informal care)**	8.91	Minimum wage between April 2021 to March 2022, Minimum wage rates for 2022 - GOV.UK (www.gov.uk)
**Special Equipment (NHS costs)**		
Stick	3.5	Hospital procurement
One Crutch	3.58	Hospital procurement
Two Crutches	7.16	Hospital procurement
Frame	18.5	Hospital procurement
Raised toilet seat	7.2	Hospital procurement
Perching stool	17	Hospital procurement
Bath support	11	Hospital procurement
Shower seat	26.77	Hospital procurement
Comode toilet	19.83	Hospital procurement
Toilet rails	4.2	Hospital procurement
Commode with 2 Zimmer Frames	56.83	Hospital procurement
**Treatment costs**		
Boot	50	Hospital procurement
Plaster	25	Hospital procurement

For peer review only

Plaster technician (cost per hour)				Unit Costs of Health and Social Care 2020/21 (PSSRU)
Physiotherapist Band 4			35	Hospital based scientific and professional, cost per hour
Physiotherapist Band 5			39	Hospital based scientific and professional, cost per hour
Physiotherapist Band 6			52	Hospital based scientific and professional, cost per hour
Private expenses				Gym sessions and other sport sessions, Child care, Home cleaner, Food delivery (e.g. take away, eating out), Other, Equipments bought privately - Expenses provided by participant in the questionnaire, Inflated to 2020/21 prices using CPI
**Medication cost (Prescribed&out-of-pocket)**				Source: British National Formulary for medications April 2022
**Medication**	**Unit cost 2022**	**Drug tariff price (one pill)**	**Pills/day based on dose assumption**	**Dose (assumption)**
Ibuprofen 200mg	0.118	0.039	3	maintenance 200–400 mg 3 times a day
Paracetamol 500 mg	0.095	0.024	4	500mg every 4-6 hours
Codeine phosphate 15 mg	0.176	0.029	6	30 mg 3–4 times a day; usual dose 15–60 mg 3–4 times a day
Co-codamol 500mg / 8 mg	0.152	0.038	4	every 4–6 hours
Naproxen 250 mg	0.121	0.040	3	250 mg every 6–8 hours as required
Tramadol hydrocloride 50 mg	0.475	0.119	4	50–100 mg every 4–6 hours
Omeprazole 10 mg	0.332	0.332	1	10 mg once daily,
Antibiotic (Ampicillin 250 mg)	6.946	0.868	8	0.5–1 g every 6 hours.
Dalteparin (Fragmin)*	5.122	5.122	1	5000 units every 12 hours
Amitryptyline hydrocloride 10mg	0.027	0.027	1	Initially 5–10 mg daily, to be taken at night; increased in steps of 10 mg at least every 2 weeks as required; maximum 30 mg per day
Oramorph 10 mg	0.442	0.074	6	Initially 10 mg every 4 hours (acute)
Oxycodone Hydrochlorid 10mg	0.368	0.184	2	Initially 10 mg every 12 hours
Voltaren gel	4.020			
Gabapentin 600mg	0.071	0.047	1.5	Initially 300 mg once daily on day 1, then 300 mg twice daily on day 2, then 300 mg 3 times a day on day 3, alternatively initially 300 mg 3 times a day on day 1, then increased in steps of 300 mg every 2–3 days in 3 divided

For peer review only

				doses, adjusted according to response; maximum 3.6 g per day.
Rivaroxaban 15mg	1.800	1.800	1	10 mg once daily
Ibuprofen gel 100g	2.400			
Co-amoxiclav 500/125mg Tablets	0.264	0.088	3	500/125 mg 3 times a day
Flucoxacillin Capsules 500mg	0.166	0.041	4	500 mg 4 times a day for 5–7 days.
Metronidazole 400mg	0.246	0.082	3	

For peer review only

Table S2. Resource utilisation

	Week 3-6 post surgery						Week 7-12 post surgery					
	Plaster (N=120)			Boots (N=123)			Plaster (N=120)			Boots (N=123)		
	Non-missing	Users	Average use (occasions)	Non-missing	Users	Average use (occasions)	Non-missing	Users	Average use (occasions)	Non-missing	Users	Average use (occasions)
**Physiotherapy services**												
Provided by GP surgery/practice – at the surgery	101	0	0.00	104	3	0.04	77	5	0.10	77	7	0.25
Provided by GP surgery/practice – at your home	102	0	0.00	104	1	0.03	76	0	0.00	77	0	0.00
Provided by a hospital – at hospital	102	0	0.00	105	0	0.00	76	0	0.00	77	0	0.00
Provided by a hospital – at home	102	4	0.05	105	5	0.05	76	2	0.03	77	1	0.01
Provided by a private clinic - at clinic	102	0	0.00	105	2	0.04	76	6	0.28	77	4	0.18
Provided by a private clinic - at home	102	0	0.00	105	0	0.00	76	1	0.04	77	0	0.00
Consultations with an Occupational Therapist	102	5	0.07	105	2	0.05	76	7	0.20	77	11	0.19
**Community-based Services**												
GP at the surgery/practice	102	3	0.04	105	12	0.27	75	7	0.13	75	10	0.28
GP at your home	102	0	0.00	105	2	0.02	75	0	0.00	75	1	0.01
GP telephone contact	102	14	0.20	105	16	0.29	75	5	0.11	75	10	0.19
Practice Nurse / Health Care Assistant at GP surgery	100	0	0.00	104	9	0.09	75	0	0.00	75	5	0.07
Practice Nurse telephone contact	100	0	0.00	103	4	0.07	75	0	0.00	75	0	0.00
Nurse at your home	101	3	0.58	104	5	0.31	75	0	0.00	75	0	0.00
Health Visitor	100	1	0.01	104	0	0.00	75	0	0.00	75	0	0.00
Social worker (all contacts, including telephone calls)	100	0	0.00	104	0	0.00	75	0	0.00	75	0	0.00
Home care/Home help	100	1	0.01	104	4	0.20	75	0	0.00	75	0	0.00

For peer review only

Food at home services (e.g. meals on wheels)	100	0	0.00	104	3	0.09	75	0	0.00	75	74	3.95
Voluntary Agency Worker/Contact	100	0	0.00	103	1	0.02	75	0	0.00	75	0	0.00
**Hospital Services**												
Accident and Emergency	102	5	0.05	105	4	0.05	75	0	0.00	76	0	0.00
Day case admission or procedure (but did not spend the night)	102	1	0.01	105	1	0.01	75	0	0.00	76	0	0.00
Admitted to hospital overnight	98	0	0.00	102	0	0.00	75	0	0.00	76	0	0.00
Outpatient appointment	99	9	0.10	104	8	0.12	75	11	0.21	76	13	0.00
**Informal care (hours)**	102	81	70.33	105	88	43.92	78	47	33.28	78	49	51.65
**Time off work (hours)**	102	48	67.56	105	60	71.91	77	27	58.71	77	29	39.64
**Use of special equipment**	Non-missing	Users	%	Non-missing	Users	%	Non-missing	Users	%	Non-missing	Users	%
Special equipment private	99	2	2.0%	104	8	7.7%	75	0	0.0%	76	1	1.3%
Special equipment NHS (prescribed)	99	6	6.1%	104	3	2.9%	77	12	15.6%	77	22	28.6%
**Use of medication (Prescribed&out-of-pocket)**	101	45	44.6%	105	64	61.0%	76	34	44.7%	77	36	46.8%
**Private costs**												
Gym sessions and other sport sessions	100	1	1.0%	105	6	5.7%	77	4	5.2%	77	6	7.8%
Child care	100	4	4.0%	105	4	3.8%	76	1	1.3%	77	0	0.0%
Home cleaner	100	9	9.0%	105	16	15.2%	76	3	3.9%	77	4	5.2%
Food delivery (e.g. take away, eating out)	100	23	23.0%	105	21	20.0%	76	5	6.6%	77	9	11.7%
Other	99	3	3.0%	103	16	15.5%	75	1	1.3%	77	4	5.2%
Travel expenses	100	34	34.0%	105	34	32.4%	76	21	27.6%	77	19	24.7%
Other major private expenses	101	4	4.0%	105	3	2.9%	77	4	5.2%	75	5	6.7%

Table S3 Costs and outcomes by trial arm and by perspective on costs (raw data)

**Cost Component**		**Boot (N=123)**		**Plaster (N=120)**	**Difference**
NHS+PSS	n	Mean (95% CI)	n	Mean (95% CI)	Mean (95% CI)
Ankle treatment (boot or plaster)	117	£203 (£200, £206)	112	£179 (£177, £181)	£24 (£21, £28)
Physiotherapy in the community	70	£15 (£7, £23)	68	£4 (£1, £7)	£11 (£3, £20)
Physiotherapy in hospital	70	£24 (£13, £35)	68	£25 (£11, £39)	-£1 (-£19, £16)
GP practice services	68	£69 (£37, £100)	66	£33 (£7, £59)	£36 (-£5, £77)
Social Care services	68	£24 (£11, £36)	66	£7 (-£0, £14)	£17 (£2, £31)
A&E and Outpatient services	68	£60 (£24, £96)	65	£29 (£17, £40)	£31 (-£7, £70)
Hospital Admissions	66	£14 (-£6, £34)	66	£14 (-£6, £34)	£0 (-£28, £28)
NHS Equipment	69	£4 (£2, £6)	68	£2 (£1, £2)	£2 (-£0, £4)
Medications	70	£6 (£4, £8)	68	£6 (£1, £10)	£1 (-£4, £5)
Societal costs					
Private physiotherapy	70	£18 (£5, £30)	68	£19 (£7, £31)	-£2 (-£19, £16)
Additional medical Equipment	68	£8 (£3, £13)	66	£0 (-£0, £1)	£8 (£3, £12)
Private community care	104	£0 (-£0, £1)	100	£2 (-£1, £4)	-£1 (-£4, £2)
Other private expenditures on services/activities	67	£105 (£74, £136)	67	£170 (£30, £311)	-£65 (-£207, £77)
Other major expenditure not reported elsewhere	68	£11 (-£0, £23)	68	£11 (£2, £21)	-£0 (-£15, £15)
Productivity Loss (time-off work and leisure)	70	£1,915 (£1,493, £2,337)	69	£2,164 (£1,565, £2,764)	-£249 (-£976, £478)
Informal Care	71	£1,042 (£652, £1,432)	70	£1,185 (£719, £1,650)	-£143 (-£746, £460)
Outcomes					
QALYs gained (Hernandez Alava and Pudney)	60	0.1168 (0.1114, 0.1223)	61	0.1246 (0.1203, 0.1288)	-0.0077 (-0.0146, -0.0009)
QALY gained (Van Hout)	60	0.1086 (0.1027, 0.1144)	61	0.1184 (0.1140, 0.1229)	-0.0099 (-0.0172, -0.0026)
Orelud and Molander (primary outcome, Week7-Week2 baseline)	93	-55.4 (-59.5, -51.3)	91	-59.0 (-62.3, -55.7)	3.6 (-1.6, 8.8)
Orelud and Molander (Week12-Week2 baseline)	78	-41.3 (-45.3, -37.4)	74	-36.1 (-39.5, -32.7)	-5.3 (-10.5, 0.0)

For peer review only

Figure S1 – Distribution of costs: total NHS+PSS, total societal costs, productivity loss and cost of informal care.

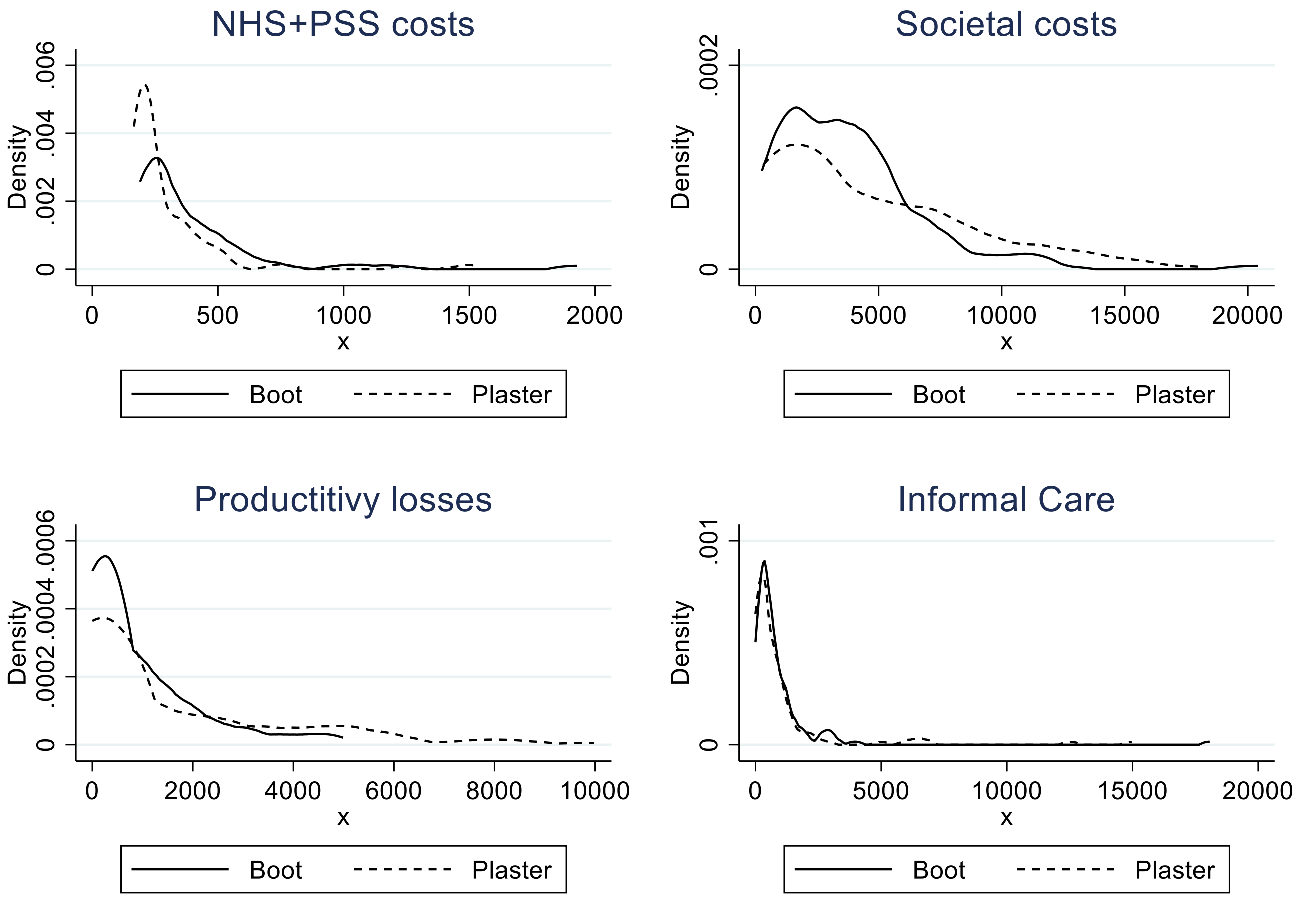

Figure S2 – Distribution of utility scores for QALY gains

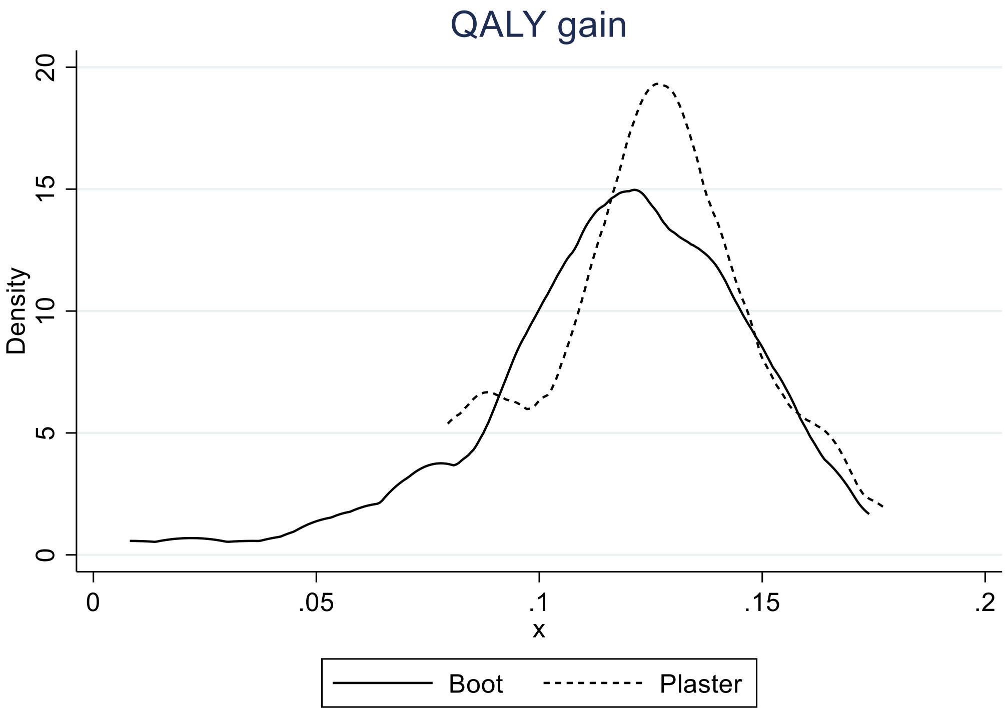

For peer review only - http://bmjopen.bmj.com/site/about/guidelines.xhtml

Figure S3 Cost-effectiveness Acceptability Curve comparing boot (intervention) to cast (control)

(a) NHS + PSS perspective

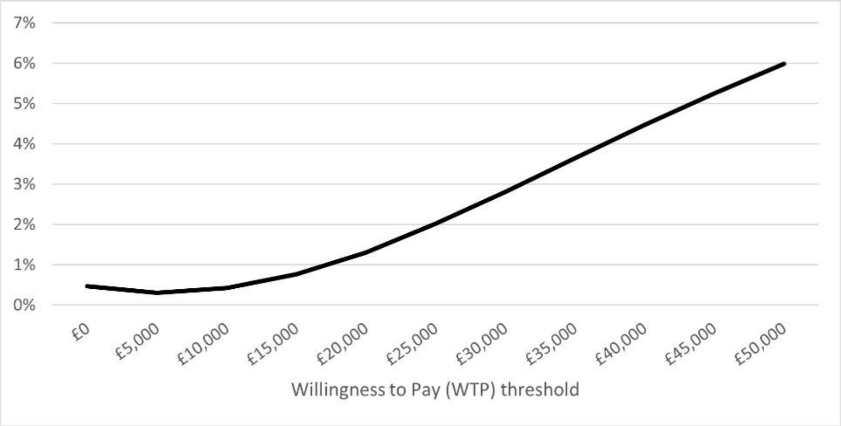

(b) Societal perspective

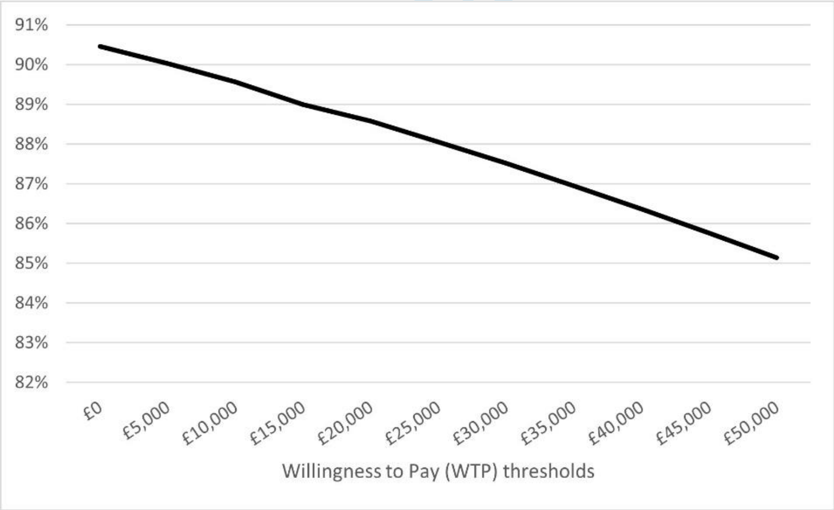

Table S4 One-way sensitivity analysis

**NHS+PSS perspective**<br>**One-way sensitivity analysis**	**Scenario 1**<br>**25% reduction in boots price**<br>Mean (95%CI)	**Scenario 2**<br>**50% reduction in boots price**<br>Mean (95%CI)	**Scenario 3**<br>**75% reduction in boots price**<br>Mean (95%CI)
Incremental Cost	£76 (£9 to £142)	£63 (-£3 to £130)	£51 (-£16 to £117)
Incremental QALY	-0.0020 (-0.0067 to 0.0026)	-0.0020 (-0.0067 to 0.0026)	-0.0020 (-0.0067 to 0.0026)
Net Monetary Benefit	-£117 (-£218 to -£15)	-£104 (-£206 to -£3)	-£92 (-£193 to £10)
Probability of Cost-effectiveness	2.2%	3.6%	5.7%

For peer review only

CHEERS 2022 Checklist

Topic	No.	Item	Location where item is reported
**Title**			
	1	Identify the study as an economic evaluation and specify the interventions being compared.	Title, Page 1
**Abstract**			
	2	Provide a structured summary that highlights context, key methods, results, and alternative analyses.	Abstract, Page 3
**Introduction**			
**Background and objectives**	3	Give the context for the study, the study question, and its practical relevance for decision making in policy or practice.	Introduction, 2nd paragraph
**Methods**			
**Health economic analysis plan**	4	Indicate whether a health economic analysis plan was developed and where available.	Methods, Overview of economic evaluation
**Study population**	5	Describe characteristics of the study population (such as age range, demographics, socioeconomic, or clinical characteristics).	Table 1
**Setting and location**	6	Provide relevant contextual information that may influence findings.	Methods, Trial design and ethics approval
**Comparators**	7	Describe the interventions or strategies being compared and why chosen.	Methods, Intervention and usual care
**Perspective**	8	State the perspective(s) adopted by the study and why chosen.	Methods, Overview of economic evaluation
**Time horizon**	9	State the time horizon for the study and why appropriate.	Methods, Trial design and ethics approval
**Discount rate**	10	Report the discount rate(s) and reason chosen.	Not used
**Selection of outcomes**	11	Describe what outcomes were used as the measure(s) of benefit(s) and harm(s).	Methods, Trial design and ethics approval
**Measurement of outcomes**	12	Describe how outcomes used to capture benefit(s) and harm(s) were measured.	Methods, Outcome data collection for the economic evaluation
**Valuation of outcomes**	13	Describe the population and methods used to measure and value outcomes.	Methods, Valuing EQ-5D-5L to derive QALYs
**Measurement and valuation of resources and costs**	14	Describe how costs were valued.	Methods, Valuing resource use to derive costs

Topic	No.	Item	Location where item is reported
**Currency, price date, and conversion**	15	Report the dates of the estimated resource quantities and unit costs, plus the currency and year of conversion.	Methods, Valuing resource use to derive costs
**Rationale and description of model**	16	If modelling is used, describe in detail and why used. Report if the model is publicly available and where it can be accessed.	Not used
**Analytics and assumptions**	17	Describe any methods for analysing or statistically transforming data, any extrapolation methods, and approaches for validating any model used.	Methods , Cost-Utility Analysis, Uncertainty and sensitivity analyses and Appendix
**Characterising heterogeneity**	18	Describe any methods used for estimating how the results of the study vary for subgroups.	Subgroup analysis was not preformed
**Characterising distributional effects**	19	Describe how impacts are distributed across different individuals or adjustments made to reflect priority populations.	Not analysed
**Characterising uncertainty**	20	Describe methods to characterise any sources of uncertainty in the analysis.	Methods , Cost-Utility Analysis, Uncertainty and sensitivity analyses
**Approach to engagement with patients and others affected by the study**	21	Describe any approaches to engage patients or service recipients, the general public, communities, or stakeholders (such as clinicians or payers) in the design of the study.	Methods, Patient and Public Involvement
**Results**			
**Study parameters**	22	Report all analytic inputs (such as values, ranges, references) including uncertainty or distributional assumptions.	Results, first paragraph and Table
**Summary of main results**	23	Report the mean values for the main categories of costs and outcomes of interest and summarise them in the most appropriate overall measure.	Results, Costs and outcomes and Cost-effectiveness base case and sensitivity analyses, Table 2 table 3
**Effect of uncertainty**	24	Describe how uncertainty about analytic judgments, inputs, or projections affect findings. Report the effect of choice of discount rate and time horizon, if applicable.	Results, Cost-effectiveness base case and sensitivity analyses, Figures 1-2 and Appendix
**Effect of engagement with patients and others affected by the study**	25	Report on any difference patient/service recipient, general public, community, or stakeholder involvement made to the approach or findings of the study	Not reported
**Discussion**			
**Study findings, limitations, generalisability, and current knowledge**	26	Report key findings, limitations, ethical or equity considerations not captured, and how these could affect patients, policy, or practice.	Discussion, Strengths and limitations
**Other relevant information**			

Topic	No.	Item	Location where item is reported
**Source of funding**	27	Describe how the study was funded and any role of the funder in the identification, design, conduct, and reporting of the analysis	End of manuscript
**Conflicts of interest**	28	Report authors conflicts of interest according to journal or International Committee of Medical Journal Editors requirements.	Submitted in the editorial system

From: Husereau D, Drummond M, Augustovski F, et al. Consolidated Health Economic Evaluation Reporting Standards 2022 (CHEERS 2022) Explanation and Elaboration: A Report of the ISPOR CHEERS II Good Practices Task Force. Value Health 2022;25. doi:10.1016/j.jval.2021.10.008

For peer review only

For peer review only

ART: **A**nkle **R**ecovery **T**rial

Does early mobilisation after **A**nkle fracture surgery enhance **R**ecovery? A randomised controlled **T**rial comparing the use of plaster versus removable support boot.

**Protocol Version and date**	Version 1.6 – 05/03/2018
**ISRCTN**	15497399
**REC Reference**	14/SC/1409
**Funder:**	NIHR Research for Patient Benefit (RfPB)
**Sponsor:**	Poole Hospital NHS Foundation Trust
**Sponsor Reference Number:**	P140218

For peer review only - http://bmjopen.bmj.com/site/about/guidelines.xhtml

General Information

This document describes the ART trial and provides information about procedures for entering patients into the trial. The protocol should not be used as an aide-memoire or guide for the treatment of other patients. Every care was taken in its drafting, but corrections or amendments may be necessary. These will be circulated to the registered investigators in the trial, but centres entering patients for the first time are advised to contact the study coordinating site to confirm they have the most up to date version. The protocol has been developed with reference to the Poole Hospital NHS Foundation Trust protocol template.

Lead applicant and Co-applicants

Chief Investigator:	Mr Bob Sangar<br>Poole Hospital NHS Foundation Trust
Consultant Surgeon:	Mr Mark Farrar<br>Poole Hospital NHS Foundation Trust
Trial Statistician:	Professor Peter Thomas,<br>Bournemouth University Clinical Research Unit
Methodologist:	Dr Sharon Docherty<br>Bournemouth University Clinical Research Unit
Qualitative Researcher:	Helen Allen<br>Bournemouth University Clinical Research Unit
Health Economist:	Dr Elsa Marques<br>University of Bristol
Senior Trial Manager:	Christopher Hayward<br>Plymouth University Peninsula Clinical Trials Unit
Lead Physiotherapist:	Rachel Martin<br>Poole Hospital NHS Foundation Trust

Operational research team contact details

**Chief Investigator:**	**Mr Bob Sangar**<br>Poole Hospital NHS Trust<br>Longfleet Rd, Poole, Dorset<br>BH15 2JB<br>Tel: 01202 665511<br>anuraag.sangar@poole.nhs.uk	**Trial Manager: (General Enquires)**	**Lee Tbaily**<br>Poole Hospital NHS Trust<br>Research & Innovation<br>Room 38, Cornelia House<br>Longfleet Rd, Poole, Dorset, BH15 2JB<br>Tel: 01202 442025<br>Lee.Tbaily@poole.nhs.uk
**Senior Trial Manager:**	**Christopher Hayward**<br>Peninsula Clinical Trials Unit<br>Rm N16, ITTC Building 1<br>Tamar Science Park<br>Derriford, Plymouth<br>PL6 8BX<br>Tel: 01752 431020<br>Christopher.Hayward-4@plymouth.ac.uk	**Data Manager:**	**Paul Williams**<br>Peninsula Clinical Trials Unit<br>Rm N16, ITTC Building 1<br>Tamar Science Park<br>Derriford, Plymouth<br>PL6 8BX<br>Tel: 01752 439828<br>Fax: 01752 315254<br>Paul.Williams@plymouth.ac.uk
**Sponsor:**	**Sarah Chessell**<br>Poole Hospital NHS Trust<br>Research & Innovation<br>Cornelia House<br>Longfleet Rd, Poole, Dorset<br>Tel: 01202 448125<br>Sarah.chessell@poole.nhs.uk	**Lead site Research Physiotherapists:**	**Rachel Martin & Adrian Hand**<br>Poole Hospital NHS Trust<br>Physiotherapy<br>Longfleet Rd, Poole, Dorset, BH15 2JB<br>Tel: 01202 448179 / 01202 442121<br>Rachel.martin@poole.nhs.uk<br>Adrian.Hand@Poole.nhs.uk

For peer review only - http://bmjopen.bmj.com/site/about/guidelines.xhtml

Table of Contents

1. STUDY SYNOPSIS 6

2. ABBREVIATIONS 8

3. INTRODUCTION 9

4. AIMS AND OBJECTIVES 11

4.1. Aim 11

4.2. Objectives 11

4.2.1. Primary objective 11

4.2.2. Secondary objectives 11

5. STUDY DESIGN 11

5.1. Study design overview 11

5.2. Design and bias considerations 13

5.3. Duration of patient participation 13

6. SETTING 13

7. OUTCOME MEASURES 13

7.1. Primary Outcome measure 13

7.2. Secondary outcome measures 14

7.2.1. Functional data 14

7.2.2. Healing status 14

7.2.3. EQ-5D-5L 14

7.2.4. Complications 14

7.2.5. Return to usual activities 15

7.3. Baseline Characteristics 15

7.3.1. Demographics and socioeconomics 15

7.3.2. Fracture characteristics 15

7.4. Resource Use 15

7.5. Adverse events 16

7.6. Patients' experiences 16

7.7. Mobilisation and adherence to exercise 16

8. PARTICIPANT SELECTION 17

8.1. Inclusion Criteria 17

8.2. Exclusion Criteria 17

8.2.1. Justification of exclusion criteria 17

8.3. Withdrawal criteria 17

9. STUDY SCHEDULE 18

9.1. Pre-trial patient management 19

For peer review only

For peer review only - http://bmjopen.bmj.com/site/about/guidelines.xhtml

9.2. Patient identification and approach ... *19*

9.3. Baseline clinic visit (2 weeks post-surgery) ... *19*
9.3.1. Consent process ... 19
9.3.2. Baseline questionnaire completion ... 20
9.3.3. Baseline CRF completion ... 20
9.3.4. Randomisation ... 20
9.3.5. Provision of treatment ... 21
9.3.6. Provision of Patient Diary ... 21
9.3.7. End of baseline visit procedures ... 21

9.4. 4-week follow-up visit (6 weeks post-surgery) ... *22*
9.4.1. 4-week follow-up questionnaire completion ... 22
9.4.2. 4-week follow-up CRF completion ... 22
9.4.1. Provision of Patient Diary ... 23
9.4.2. End of 4-week follow up visit procedures ... 23

9.5. 5-week follow-up questionnaire completion ... *23*

9.6. 10-week follow-up (12 weeks post-surgery) ... *24*
9.6.1. 10-week follow-up questionnaire completion ... 24
9.6.2. 10-week follow-up CRF completion ... 24

9.7. Telephone interviews ... *25*

9.8. Radiographic Evaluation ... *25*

10. INTERVENTION DETAILS ... 25

10.1. Removable support boot ... *25*

10.2. Plaster Cast ... *26*

10.3. Associated Risks ... *26*

11. ASSESSMENT OF SAFETY ... 26

11.1. Standard definitions ... *26*

11.2. Reportable events ... *27*

11.3. Procedures for Serious Adverse Event reporting ... *27*
11.3.1. Assessment of severity and causality ... 27
11.3.2. Assessment of expectedness ... 29

11.4. Processing Serious Adverse Event report forms ... *29*
11.4.1. Onward reporting ... 29

12. STATISTICS AND DATA ANALYSIS ... 30

12.1. Sample Size Calculation ... *30*

12.2. Description of statistical methods ... *30*
12.2.1. Baseline descriptives: ... 30
12.2.2. Primary outcome: ... 31
12.2.3. Secondary outcomes: ... 32

12.3. Qualitative Component ... *33*

12.4. Economic Evaluation ... *33*

13. ETHICAL, REGULATORY, ADMINISTRATIVE AND QUALITY ASSURANCE ... 35

13.1. Ethical considerations ... *35*

13.2. Declaration of Helsinki ... *36*

For peer review only - http://bmjopen.bmj.com/site/about/guidelines.xhtml

13.3. Research Governance *36*

13.4. Study Management *36*

13.5. Trial Steering Committee *36*

13.6. Monitoring *37*

13.7. Audit and Inspection *37*

14. DATA HANDLING AND RECORD KEEPING 37

14.1. Participant Numbering *37*

14.2. Source data *37*

14.3. Data Entry *37*

14.4. Data confidentiality and security *37*

14.5. Archiving *38*

15. FINANCE, INDEMNITY AND INSURANCE 38

15.1. Funding *38*

15.2. Sponsor *38*

15.3. Indemnity *38*

16. PUBLICATION POLICY AND INTELLECTUAL PROPERTY 38

16.1. Publication Policy *38*

16.2. Intellectual Property *38*

17. REFERENCES 39

Figure 1 Study design flowchart 12

Table 1: Tabulated Study Schedule 18

Table 2: Definitions of Causality 29

For peer review only

For peer review only - http://bmjopen.bmj.com/site/about/guidelines.xhtml

1. STUDY SYNOPSIS

**Study title**	Does early mobilisation after **A**nkle fracture surgery enhance **R**ecovery? A pragmatic multi-centre randomised controlled **T**rial with qualitative component and health economic analysis comparing the use of plaster versus removable support boot **(ART)**.
**Short study title:**	The Ankle Fracture Recovery Trial **(ART)**
**Study design**	This study is designed as a pragmatic multi-centre randomised controlled trial with qualitative component and health economic evaluation comparing plaster cast and support boots as methods of post-operative ankle fracture management.
**Study participants**	***Inclusion Criteria***<br><br>• Received surgery for fixation of unstable ankle fracture<br>• Provision of informed consent to participate<br><br>***Exclusion criteria***<br>• Under 16 year olds (skeletally immature).<br>• Poor skin condition at operation site.<br>• Serious concomitant disease (e.g. stroke, osteoporosis, arthritis).<br>• Diabetic neuropathy/other sensory neuropathy (lack of sensation).<br>• Non-ambulatory prior to injury.<br>• Active leg ulceration.<br>• Patients who are unable to understand the study information or unable to complete the outcome questionnaires.<br>• Surgeon concerned about quality of fixation/integrity of wound.<br>• Fracture requiring further stabilisation in/around the ankle (e.g. syndesmosis).<br>• Open ankle fracture (bone broken through skin).<br>• Participant is a participant in other concurrent interventional research which may over-burden the participant or confound data collection.<br>• Concomitant injuries which will have a confounding effect on rehabilitation in the opinion of the investigator.
**Number of participants**	276 patients in total (138 in each treatment group).
**Follow-up duration**	Patients will be followed up in clinic at 4 weeks post-baseline (6 weeks post-operatively) and via questionnaires), at 5 weeks post-baseline (7 weeks post-operatively) and 10 weeks post-baseline (12 weeks post-operatively). Qualitative telephone interviews with up to 20 participants will take place after the 10-week follow up.

For peer review only - http://bmjopen.bmj.com/site/about/guidelines.xhtml

**Planned study period**	48 months (37 month recruitment period)
**Study aim**	To evaluate the relative effectiveness and cost-effectiveness of two methods of post-operative ankle fracture management (plaster versus removable support boot allowing range of movement) and to provide evidence-based recommendations for best care in clinical practice.
**Study objectives**	1. To determine whether there is a difference in ankle function between the two types of treatment.<br>2. To determine whether there is a difference in quality of life between the two treatments.<br>3. To estimate which is the cost-effective treatment option to inform decision-making.<br>4. To explore patient experiences and the psychological and social impact of their treatment.
**Outcome measure data**	**Primary outcome:**<br>The primary outcome measure for this study is the Olerud and Molander ankle score at five weeks after randomisation.<br><br>**Secondary outcomes:**<br>• Ankle functional data (range of movement, weight-bearing)<br>• Standardised measure of general quality of life (EQ-5D-5L)<br>• Healing status<br>• Complications<br>• Return to Usual Activities<br><br>**Other data collected;**<br>• Baseline characteristics<br>• Healthcare resource use<br>• Adverse events<br>• Mobilisation and adherence to exercise
**Qualitative component**	Telephone interviews conducted at around the 10 week follow up time point with up to 20 participants to address:<br><br>• Experiences of each type of treatment (plaster/boot).<br>• Lifestyle implications – individual (e.g. washing), family, social, psychological, work, financial, residential, diet, transport (e.g. driving), leisure activities, and independence.<br>• Expectations, mind-set, confidence, including the perceived views of carers where appropriate.
**Interventions**	Patients will be allocated in a 1:1 ratio with both groups weight-bearing as tolerated:<br><br>1. **Plaster below knee** i.e. immobilised for four weeks.<br>2. **Removable support boot** with range of movement for four weeks.

For peer review only - http://bmjopen.bmj.com/site/about/guidelines.xhtml

2. ABBREVIATIONS

AE	Adverse Events
BUCRU	Bournemouth University Clinical Research Unit
CI	Chief Investigator
CEAC	Cost Effectiveness Acceptability Curves
CRF	Case Report Form
DVT	Deep Vein Thrombosis
HE	Health Economist
ICER	Incremental Cost Effectiveness Ratio
ICH GCP	International Conference on Harmonisation, Good Clinical Practice
IEP	Image Exchange Portal
INMB	Incremental Net Monetary Benefit
ISRCTN	International Standard Randomised Clinical Trial Number
NIHR	National Institute for Health Research
OMAS	Olerud and Molander Ankle Score
PenCTU	Peninsula Clinical Trials Unit
PI	Principal Investigator
PSS	Prescribed Specialised Services
QALY	Quality Adjusted Life Years
RCT	Randomised Controlled Trial
REC	Research Ethics Committee
RfPB	Research for Patient Benefit
SAE	Serious Adverse Events
TMG	Trial Management Group
TSC	Trial Steering Committee
WPAI	Work Productivity and Activity Impairment Questionnaire

For peer review only - http://bmjopen.bmj.com/site/about/guidelines.xhtml

3. INTRODUCTION

Approximately two million ankle fractures occur annually in the United Kingdom affecting 3.6% of the population, 75% in under 60 year olds[1], of which a high proportion need surgical fixation, with considerable cost to the National Health Service. Indeed, 7.2% of all fractures admitted to Poole Hospital are ankle fractures and 197 underwent surgical fixation in 2011[2] illustrating the scale of the issue (audit data suggest 104 in Basingstoke in 2012 and 76-80 in six month periods in 2009 and 2010 respectively in Portsmouth). Ankle fractures are a cause of short-term and sometimes long-term disability and pain. These injuries frequently require many weeks off work with subsequent economic consequences[3]. Six weeks of immobilisation in plaster is frequently advocated for all ankle fractures *not* requiring surgery. It is also recognised practice to immobilise a broken ankle after surgery for comfort, support and to allow the wound to heal. This tends to be with a plaster because it is cheap and readily available in the operating theatre and has been used for decades.

However, there has been no consensus for the management of ankle fractures post-operatively for those ankle fractures that need surgical fixation. Therefore, current treatment is not standardised in many Trusts in the National Health Service. There is evidence to suggest that removing plasters and wearing removable boots that allow early weight-bearing and range of movement of the ankle, maximises rehabilitation potential.

A number of studies have investigated the benefits of early weight-bearing and mobilisation following ankle fracture fixation. Early return to function should be the principal goal following surgical management[3]. Traditionally, patients have been managed in a plaster cast and have been strictly non-weight-bearing (hopping on their unaffected leg).

However, immobilisation can result in reduced range of movement and muscle deterioration[4-6]. Early mobilisation of ankles following open reduction and surgical internal fixation provides short-term benefits in improved range of movement, reduced swelling, pain, an earlier return to work, improved patient satisfaction, and better ankle function[7].

At Poole Hospital, fitting patients with a plaster following open reduction and surgical internal fixation costs approximately £20.84 for materials plus plaster technician labour costs for 10-15 minutes per case. Although there are a range of boots available on the market at varying prices, those used are chosen for their quality and value for money – for example, the pneumatic walker boot currently in use at Poole Hospital costs £52.39 and we expect it may be equally labour intensive (information provided by plaster technicians).

However, the boot may be more comfortable as it can be removed at night and for washing, and ultimately improves patient satisfaction[8]. Despite boots having fewer limitations than plaster, they can still be considered cumbersome and disposal of plasters and boots both have environmental impact.

For peer review only - http://bmjopen.bmj.com/site/about/guidelines.xhtml

Furthermore, some studies report conflicting and inconclusive findings with regards to complications. Lehtonen et al[9] found wound infections in 6.6% of those who mobilised early and a high incidence of deep vein thrombosis. On the other hand, Vioreanu et al[6] found patients who exercised once primary wound healing had taken place had fewer wound infections and the immobilised group had problems with deep vein thrombosis. Farsetti et al[10] compared two case series of 22 patients and found no correlation between wound complications and continuous passive movement applied for the first three weeks post-operatively.

Although there are two current trials underway, one in London[11], the other in Toronto[12], the London study is a smaller study (n=76) and does not encourage active movement through range of movement exercises whilst the Toronto study is unrealistic not allowing weight-bearing in plaster. The most recent systematic review by Thomas et al[3 (p 674)] and a Cochrane report by Lin et al[13 (p 431)], both recommend the need for more research with well-designed and conducted trials with adequate numbers and reporting. There is limited evidence supporting the use of a removable type of immobilisation and active range of movement and no previous studies, to the research team's knowledge, have investigated the socio-economic factors and psychological impact which may be related to return to function, independence, and work.

This research is needed now to ensure that the post-operative management of patients who have sustained an ankle fracture is consistent with best practice and considerate of patient experience. The research is particularly timely in this economic climate to allow patients to optimise their rehabilitation potential and return to full function and normal employment as soon as possible. There is currently no definitive study advising on the optimal treatment and rehabilitation of ankle fractures following surgery. This randomised study aims to objectively provide an answer. In particular, the study will provide guidance on which form of post-surgical treatment is more reliably able to restore an individual's independence, function, and mobility after ankle fracture surgery; as well as the probability of being a cost-effective treatment option for the NHS. It will also investigate whether the treatment regime influences a patient's return to work as well as exploring the wider socio-economic implications of the two treatments.

As part of patient and public involvement for the design of this study, twelve telephone consultations were conducted with service users who had received treatment for ankle fractures. One of the most important issues to patients suffering from ankle fractures was the lack of hygiene when immobilised in a plaster. The advantage provided by a removable boot in terms of being able to visualise and mobilise tissues and joints has been alluded to in several studies.

If there is a clear advantage of one treatment over another in terms of outcomes and costs, then treatment can be standardised with recommendations being made for local and national policy guidelines. This will directly benefit patients, employers, healthcare providers and the wider economy.

For peer review only - http://bmjopen.bmj.com/site/about/guidelines.xhtml

4. AIMS AND OBJECTIVES

4.1. Aim

To evaluate the relative effectiveness and cost-effectiveness of two methods of post-operative ankle fracture management (plaster versus removable support boot with range of movement) and to provide evidence-based recommendations for best care in clinical practice.

4.2. Objectives

4.2.1. Primary objective

- To determine whether there is a difference in function between the two types of treatment.

4.2.2. Secondary objectives

- To determine whether there is a difference in quality of life between the two treatments.
- To determine whether there is a difference in healing, complications and adverse events between the two treatments.
- To determine whether there is a difference between return to work, driving and usual activities between the two treatments
- To determine which is the most cost-effective treatment option if provided in the NHS.
- To explore patient experiences and the psychological and social impact of their treatment.

5. STUDY DESIGN

5.1. Study design overview

This study is designed as a pragmatic multi-centre randomised controlled trial with qualitative component and health economic evaluation comparing plaster cast and removable support boots as methods of post-operative ankle fracture management.

For peer review only - http://bmjopen.bmj.com/site/about/guidelines.xhtml

Figure 1 Study design flowchart

Illustrating flow of patients through the trial and the time points for data collection.

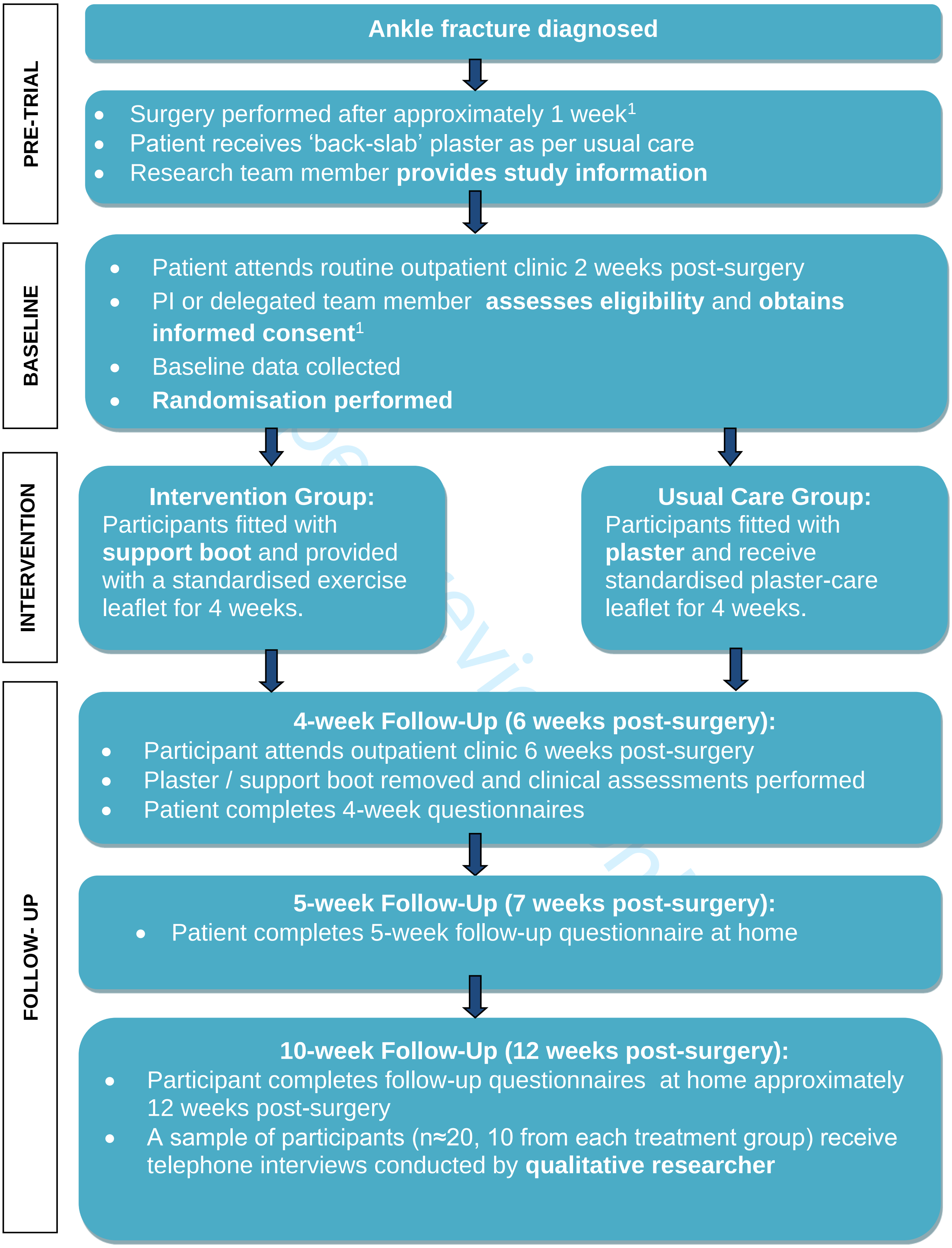

[1] Patients not requiring surgery, or who are ineligible or unwilling to participate in the trial will be excluded from the trial and managed as per usual care.

For peer review only - http://bmjopen.bmj.com/site/about/guidelines.xhtml

5.2. Design and bias considerations

The nature of the two interventions clearly makes it impossible to blind the patient or treating clinicians. Moreover the appearance of the leg at the follow-up appointments will make the allocation evident, so that those involved in outcome assessment also cannot be blinded. However, the trial statistician and data analysts will remain blinded to group allocation until the analyses are completed. Furthermore, the radiographic evaluations of healing status performed by the Chief Investigator at the end of the study (see section 9.8 Radiographic Evaluation) will be performed in a blinded manner.

5.3. Duration of patient participation

The expected duration of patient participation is approximately 10 weeks starting from randomisation and collection of baseline measures at the first clinic visit. Up to twenty participants will be selected for qualitative interviews soon after the 10-week time point. End of patient participation will be defined as the completion of data collection for the last patient followed-up in the study.

6. SETTING

The study will be conducted at three investigator sites initially; Poole Hospital NHS Foundation Trust, Basingstoke and North Hampshire NHS Foundation Trust and Queen Alexandra Hospital, Portsmouth Hospitals NHS Trust. Other sites may be invited to participate in the event that recruitment rate is lower than anticipated, subject to REC and NHS approvals. Conduct of the trial at each site will be led by a local Principal Investigator with support from research personnel trained in good clinical practice. Support will also be required from a physiotherapist(s) to perform protocol-specific clinical assessments, for which training will be provided.

7. OUTCOME MEASURES

This section is intended to list, describe and justify choice of outcomes rather than to describe how the data are collected for research purposes. Data collection is addressed in Section 9.

7.1. Primary Outcome measure

The primary outcome measure for this study is the Olerud and Molander[14] ankle symptom scale at five weeks after randomisation. The five week post-baseline data collection period is the critical period, as this is when it is anticipated that the group who had boots will have a greater level of function[3,6]. Most patients in both groups will no longer be using their allocated treatment and will have had at least 3 days without a plaster cast or boot to enable functional recovery of the ankle to be assessed. The study will also compare the ten week Olerud and Molander[14] ankle score to check whether any differences between groups still remain, and to see whether the ten week measures have returned to their (estimated) pre-injury values.

For peer review only

For peer review only - http://bmjopen.bmj.com/site/about/guidelines.xhtml

7.2. Secondary outcome measures

7.2.1. Functional data

Functional data including range of movement (dorsiflexion, plantarflexion, inversion, eversion (degree angles)), circumferential swelling and weight-bearing status at four weeks post baseline. Range of motion will be assessed using a goniometer. Weight-bearing will be classified as touch weight-bearing, partial weight-bearing or full weight-bearing. Circumferential swelling of the injured ankle will be measured using a tape measure. Circumference of the non-injured ankle will be measured in the same way and used as a comparison. Range of movement assessments and circumferential measurements must be conducted by a physiotherapist trained in the requirements of the study protocol and recorded in the patient's medical record.

7.2.2. Healing status

Healing status will be determined by the Chief Investigator's blinded review of pre-operative, intra-operative and post-operative X-ray films at the end of the study (see section 9.7 Radiographic Evaluation). The review will evaluate integrity of the surgical metal work (displaced / undisplaced) and whether or not there is evidence of fracture healing at the 4 week time point.

7.2.3. EQ-5D-5L

EQ-5D-5L will be completed by participants at baseline, 4-week, 5 week and 10-week time points. There is likely to be a marked difference in quality of life once the boot/ plaster is removed, and so measurements will be made at both 4 weeks (with the boot/ plaster on) and 5 weeks (with the boot/ plaster usually off). We anticipate that measurement at 4 weeks will attribute to the restrictive nature of the boot/ plaster, whilst at 5 weeks will collect data on recovery/ healing. The EQ-5D-5L is a standardised descriptive system of health-related quality of life states consisting of five dimensions (mobility, self-care, usual activities, pain/discomfort, anxiety/depression) each of which can take one of five responses. It captures relevant issues e.g. walking, problems washing/dressing, as well as problems with usual activities including work and family/leisure activities, pain, and psychological impact[15]. It will be used to derive quality adjusted life years (QALYs).

7.2.4. Complications

Complications including; Blisters (presence and severity; minor/major), Pressure sores (presence and severity; grade 1-5), Failure of fixation (presence; medial/lateral/both), Operation/revision surgery, Wound breakdown, Wound infection (presence and severity; Minor (treatable with antibiotics/dressing) / major (needs surgical debridement), Compartment Syndrome, DVT (presence; below knee/above knee), Nerve Injury (presence; sensory/motor/both) and Pulmonary embolism occurring up to 12 weeks post-surgery will be recorded. Complications recorded will include those reported by patients, identified by the PI or delegated member of the research team during their routine clinical assessment and from their review of hospital records.

For peer review only

For peer review only - http://bmjopen.bmj.com/site/about/guidelines.xhtml

7.2.5. Return to usual activities

At baseline, patients will be asked for their employment status prior to injury (unemployed, homemaker, volunteer, full time education, part-time paid employment, full time paid employment). At 10 weeks participants will be asked for their current employment status and will be asked whether their ankle injury has an impact on every day activities (0 to 10 scale anchored by no effect on daily activities and completely prevented daily activities).

Absenteeism (days off work), presenteeism (feeling less productive at work), and time off usual social activities, are productivity losses that we will measure at 4 and 10 weeks, by questions included in the resource use questionnaire. Participants will also be asked if they drove pre-injury, whether they had started driving again, and the date they resumed driving.

7.3. Baseline Characteristics

7.3.1. Demographics and socioeconomics

Gender, age, marital status living situation, education level, employment status, driving status and pre-surgery height and weight/BMI will be collected at baseline.

7.3.2. Fracture characteristics

Characteristics of the fracture (classification, pattern) and a description of the hardware used during surgery will be recorded following a blinded review of radiographs performed by the Chief Investigator (see section 9.7 Radiographic Evaluation). Classification will be coded as Weber A, B or C, pattern will be coded as medial malleolar fracture or deltoid ligament injury .Fracture Complexity will be reported in the baseline CRF and will be coded as simple fibular fracture and comminuted fracture.

7.4. Resource Use

(i) Resources associated with administration of treatment in both arms (including materials used and time and grade of staff involved in fitting/removing the plaster/boot and providing the necessary instruction to patients) will be captured by the research team in the CRF at the 2 week post-surgery (baseline) and 6-week post-surgery (4 week post-randomisation) appointment in clinic.

(ii) Resources delivered by the treating hospital[1] at 10 weeks from randomisation will be collected from a review of patients' hospital notes at the end of the patient's participation. Data on inpatient admissions, outpatient appointments and outpatient therapies provided, as well as diagnostic tests performed will be collected onto CRF's by the research team from randomisation (2-week post-surgery) until last follow-up (12 weeks post-surgery). Date of event will be collected, to allow for costing the two different periods of resource use, in the two economic analyses.

[1] In this context, 'treating hospital' refers to the hospital in which the ankle fracture surgery is performed.

For peer review only - http://bmjopen.bmj.com/site/about/guidelines.xhtml

(iii) Participants' use of other health and social care resources, as well as informal care and loss of productivity, will be captured at the 4-week and 10-week time points via patient-completed questionnaires. Patient reported data will be collected from randomisation on:

(a) Community-based health care services used. This will include contacts with GP doctor and nurses at the GP practice, at home or by telephone, and other health services required such as community physiotherapist and occupational therapist visits Use of concomitant medications will also be patient reported.
(b) Secondary care visits to hospitals
(c) Use of social services, such as home help, food at home services and contacts with social worker.
(d) Productivity losses, including a measurement of absenteeism and presenteeism, by adapting a few questions from the Work Productivity and Activity Impairment Questionnaire (WPAI): General Health Version 2.0[16], a licence-free, well-known and validated questionnaire commonly used to measure productivity losses when patients take time-off work or are unproductive at work[17-18.] We will also collect losses in leisure time and informal care from friends and relatives.
(e) Private expenses, such as travel, privately paid therapies or lost income.

The primary outcome is collected at 5 weeks post randomisation. Collection of resource use should follow the same time frame, so that costs and outcomes can be compared within the period. However, we feel strongly that the first resource use questionnaire should be administered face-to-face in clinic at 4 weeks, and not at the 5 week data collection point. A first resource use questionnaire administered in clinic will allow patients to interface with the research team to clarify any queries; check the questionnaire for missing data; and instruct patients on how to complete the second questionnaire at home at 10 weeks. Patients' lives (and their resource use) will be very different after their cast or boot is removed at the 4 week visit and this may affect their recollection of health care needs. In addition, including resource use questions at the 5 week time point (3-10 days post 4 week appointment), would increase patient burden and jeopardize completeness of the primary clinical outcome. We will later approximate, using statistical methods, cost assumptions for 3-10 day incremental cost period.

7.5. Adverse events

Serious adverse events will be collected from the point of randomisation at each data collection time point for the duration of the study. See section 11 (Assessment of safety) for details.

7.6. Patients' experiences

Patients' experiences of their treatment will be explored via telephone interviews conducted by the qualitative researcher in the team. See section 12.3 (Qualitative component) for details.

7.7. Mobilisation and adherence to exercise

Data will be collected to assess whether participants have adhered to instructions to complete exercises. Physiotherapists will make recommendations for participants to exercise and mobilise their ankle as often in a day as pain allows based on an

For peer review only

For peer review only - http://bmjopen.bmj.com/site/about/guidelines.xhtml

advice leaflet specific to their treatment allocation (recommended at least 3 times a day.) Compliance will be defined as once per day or more.

8. PARTICIPANT SELECTION

8.1. Inclusion Criteria

Patients must satisfy the following criteria to be enrolled in the study:

- Received surgery for fixation of unstable ankle fracture
- Provision of informed consent to participate.

8.2. Exclusion Criteria

Patients who meet any of the following criteria will be excluded from study participation:

- Under 16 years old (skeletally immature).
- Poor skin condition at operation site.
- Serious concomitant disease (e.g. stroke, osteoporosis, arthritis).
- Diabetic neuropathy/other sensory neuropathy (lack of sensation).
- Non-ambulatory prior to injury.
- Active leg ulceration.
- Patients who are unable to understand the study information or unable to complete the outcome questionnaires.
- Surgeon concerned about quality of fixation/integrity of wound.
- Fracture requiring further stabilisation in/around the ankle (e.g. syndesmosis).
- Open ankle fracture (bone broken through skin).
- Participant is a participant in other concurrent interventional research which may over-burden the participant or confound data collection.
- Concomitant injuries which will have a confounding effect on rehabilitation in the opinion of the investigator.

8.2.1. Justification of exclusion criteria

Patients with serious concomitant diseases or concomitant injury are excluded because they may have different treatment and rehabilitation options. Patients with sensory neuropathy are excluded as they may be prone to developing pressure ulcers caused by poorly-fitting plaster casts/boots. Patients who have suffered an open ankle fracture are excluded because they are at greater risk of developing infection.

8.3. Withdrawal criteria

Participants have the right to withdraw their participation in the study at any time without affecting the standard of care the patient receives. Clinicians may also advise patients to withdraw from the study should they acquire significant concomitant disease during the course of this treatment. Once withdrawn, the patient will be advised to discuss their further care plan with their consultant. Withdrawal will not adversely affect patients' care in any way.

For peer review only

For peer review only - http://bmjopen.bmj.com/site/about/guidelines.xhtml

9. STUDY SCHEDULE

This section describes conduct of the study in chronological order, detailing procedures for data collection at each of the time points.

Table 1: Tabulated Study Schedule

A tabulated study schedule to illustrate all data collection at each time point of the study;

TIMEPOINT	Week -2 (Surgery)	Baseline[1] (+2 weeks)	Week 4[1] (+6 weeks)	Week 5[2] (+7 weeks)	Week 10[2] (+12 weeks)	End of Study
**PRE-TRIAL:**						
Surgery	X					
Patient Information	X					
**ENROLMENT:**						
Eligibility screen		X				
Informed consent		X				
Baseline fracture characteristics		X				X[3]
Baseline socio-demographics		X				
Randomisation		X				
**INTERVENTIONS:**						
Plaster Cast		●———	———●			
Removable Support Boot		●———	———●			
Time to fit / remove Intervention		X	X			
**ASSESSMENTS:**						
Olerud & Molander Questionnaire		X		X	X	
EQ-5D-5L Questionnaire		X	X	X	X	
Resource Use[4]			X		X	
Return to usual activities					X	
Range of Ankle Movement			X			
Weight Bearing Status			X			
Circumferential Swelling			X			
Complications			X		X	
Serious Adverse Events		●———	———	———	———●	
Healing Status						X[3]
Participant Interviews					X	

[1] Baseline and 4-week follow-up data collected during participants' clinic visits
[2] 5-week and 10-week follow-up data completed by participants at home (sections 9.5 and 9.6)
[3] Healing status, fracture classification and pattern will be obtained by CI's blinded review of X-rays at end of study
[4] Resource use data collected primarily by patient-completed questionnaire. Services delivered by the treating hospital will be obtained by research teams' review of hospital records

For peer review only - http://bmjopen.bmj.com/site/about/guidelines.xhtml

9.1. Pre-trial patient management

Patients will undergo surgical fixation in accordance with local policy and practice. After surgery and prior to discharge, patients will be fitted with a 'back slab' plaster; a plaster support which temporarily immobilises the broken ankle and which has an incomplete front section so as to accommodate swelling. Prophylactic anticoagulation therapy (minimising risk of developing a blood clot) will be given as appropriate. This post-operative management will be provided in accordance with local policy and practice before the patient is discharged from hospital.

9.2. Patient identification and approach

Surgeons at participating sites will be made aware of the study and the profile of potentially eligible patients by the PI. Potentially eligible patients will be approached by a member of the local research team (employees of the Hospital Trust, usually research physiotherapists or research nurses) to discuss the ART trial - preferably prior to being discharged from hospital following their ankle surgery. In the event that the patient is discharged before they can be approached, a member of the research team may contact the patient at home. The research team member will briefly describe the trial and will provide the patient with a Participant Information Sheet (PIS). Patients will be advised that they will be invited to take part in the trial when they attend their routine post-operative outpatient visit, and that they may contact a member of the research team in the meantime with any queries (contact details given in the PIS). Between receiving the PIS and attending their routine post-operative outpatient visit, patients will have up to 2 weeks to consider participating in the trial but importantly, they must have no less than 24 hours. A record of the number of patients undergoing surgical fixation for unstable ankle fracture and those approached will be maintained by the research team at each site.

9.3. Baseline clinic visit (2 weeks post-surgery)

9.3.1. Consent process

At the routine post-operative outpatient visit, typically around two weeks after surgery, patients will be approached by the PI or delegated member of the research team in order to discuss participation in the trial. Potentially eligible patients who express willingness to participate will be asked to provide consent in accordance with Good Clinical Practice. The consent process will involve a full explanation of the trial given by the person taking consent. The person responsible for taking consent will be trained in GCP and Informed Consent. Patients will be advised that they are under no obligation to take part and that their ongoing care will be unaffected if they choose not to take part. Patients who choose not to take part will be asked to provide a reason for declining, but will be told that they do not have to give a reason if they wish not to. Patients will also be advised that they are free to withdraw from the trial at any time and that doing so will not affect their ongoing care.

Following this discussion, patients who are willing to participate will be asked to complete, sign and date the trial consent form, which will also be signed and dated by the person obtaining consent.

Eligibility will be determined via clinical assessment, patient questioning and review of the patients' hospital notes. The PI is responsible for confirming eligibility and for

For peer review only - http://bmjopen.bmj.com/site/about/guidelines.xhtml

obtaining consent but may delegate these duties appropriately to other members of the research team. Such delegation will be captured on a site-specific site delegation log. Any person obtaining written informed consent must have current GCP training and have received trial-specific training from the trial team. No clinical trial procedures will be conducted prior to taking consent from the participant.

A copy of the signed Informed Consent form will be given to the participant. The original signed form will be retained in the Investigator Site File and a copy placed in the patient's hospital notes. Note that any new information that arises during the trial that may affect participants' willingness to take part will be reviewed by the Trial Steering Committee. If necessary, this information will be communicated to all participants and they will be asked to re-confirm consent in light of the new information.

Patients who are found to be ineligible or who are unwilling to participate will be managed as per usual care. Reasons for non-participation (where given) will be recorded by the research team at each site.

9.3.2. Baseline questionnaire completion

After eligibility has been confirmed and written consent obtained, and prior to randomisation, patients will be provided with a questionnaire booklet to be completed during the visit. The baseline questionnaire booklet will comprise the following questions;

- Demographic and socio-economic questions
- Pre-injury Olerud and Molander (1984) ankle score (see section 7.1)
- EQ-5D-5L (see section 7.2)

9.3.3. Baseline CRF completion

A member of the research team will transcribe the following information from the patient's hospital record into the trial case report form (CRF):

- Participant information; name, date of birth, address contact details and preferred method of contact (to facilitate sending and retrieval of questionnaires and reminder alerts.)
- Fracture information; date of fracture, date of surgery, fracture side, complexity (Simple or comminuted fracture.)
- Weight and Height to compute Body Mass Index (BMI); participants may be weighed and measured at this visit if a recent weight and height is not available in the hospital notes.

9.3.4. Randomisation

Following completion of baseline information collection, a member of the research team will randomise the participant to one of the two treatment groups; Removable support boot, or plaster.

Patients will be randomised in order to minimise systematic bias, using a secure web-based system designed and maintained by the Peninsula Clinical Trials Unit

For peer review only

(PenCTU) according to the Unit's standard operating procedures. Computer generated randomisation codes prepared by the PenCTU will be kept secure, with access restricted to the data management team at the PenCTU. Participants will be allocated to the support boot group (intervention) or to the plaster group (control) in a 1:1 ratio, using random, even, block sizes between 2 and 8 to generate the allocation sequence and achieve balance in the numbers of participants allocated to each group. Randomisation will be stratified by site. The web-based randomisation system will be accessed securely via unique log-in details provided to each local team member by the CTU. The system ensures that allocation is appropriately concealed until the point of randomisation.

Upon randomisation, each participant will be assigned a unique Trial Number which will be used as the principal identifier on trial data collection forms and questionnaires. The research team member will disclose the allocation to the participant and will ensure the allocation is followed as described below.

9.3.5. Provision of treatment

Following allocation to treatment group, the patient will be directed to the facility (e.g. plaster room) in order to receive treatment. The support boot/below knee plaster (as per allocation) will be fitted by the plaster room technicians in accordance with local practice once any clips/stitches are removed. The time taken and materials used by plaster room technicians in fitting the boot/plaster in each case will be recorded on a designated form by the research team. A physiotherapist trained in the requirements of the study protocol will provide the patient with an advice leaflet specific to the participant's treatment allocation and teach them the exercises detailed. A member of the research team will also record the time the physiotherapist spends on educating patients about their treatment and the associated leaflets. These data will be used to cost the delivery of treatment in both arms for the economic evaluation as described in section 12.4.

9.3.6. Provision of Patient Diary

Completion of follow-up questionnaires may be difficult for some patients. Missing data and recall bias are frequent problems when asking patients to retrospectively recollect events at follow-up time points.

At baseline and 4 weeks, a research team member will also therefore provide each participant with a trial-specific Patient Diary and verbal instructions as to how this could be used. Participants will be advised to use the diary as an aide-memoire to prospectively record adverse events, healthcare resource use (visits to or from healthcare professionals such as GPs, community nurses, hospital admissions etc.) to facilitate completion of questionnaires at the next visit.

9.3.7. End of baseline visit procedures

The research team will ensure the following before the patient leaves the hospital:

- The baseline questionnaires are as complete as possible. If data are missing, any missed questions will be highlighted to the patient.
- The patient has the correct advice leaflet (according to their treatment allocation) and the Patient Diary.
- The patient is aware of when their next appointment is scheduled.

For peer review only - http://bmjopen.bmj.com/site/about/guidelines.xhtml

A member of the research team will subsequently ensure that:

- The patient's participation in the trial is appropriately recorded in the hospital records and that hospital notes are labelled / annotated in accordance with local practice.
- The CRF with data collected on delivery of treatment is completed.
- The approved GP letter is sent to the patient's General Practice.

The original completed questionnaires and CRF pages will be sent to PenCTU using FREEPOST envelopes – copies of these should be retained at the local site.

9.4. **4-week follow-up visit (6 weeks post-surgery)**

Participants in both treatment groups will attend a routine six-week post-operative appointment. Participants will have their plaster or support boot removed (unless otherwise indicated), routine clinical assessments and x-rays will be performed to guide the participant's subsequent management. 4-week follow up data will be collected at this visit.

9.4.1. 4-week follow-up questionnaire completion

The participant will be provided with a questionnaire to be completed during the visit. The 4-week questionnaire booklet will comprise:

- EQ-5D-5L (see section 7.2)
- Resource use questionnaire (see section 7.4)

Participants will be encouraged to use their Patient Diary to facilitate answering questions as appropriate.

9.4.2. 4-week follow-up CRF completion

Following removal of the plaster or support boot, the patient will receive routine clinical assessment and a member of the research team will transcribe the following information from the patient's hospital records into the trial case report form (CRF):

- Range of ankle movement (see section 7.2)*
- Weight-bearing status (see section 7.2)
- Circumferential swelling (see section 7.2)*
- Complications and associated antibiotic usage (see section 7.2)
- Time required to remove boot/ or plaster and re-fit if necessary (see section 7.4)
- Mobilisation and adherence to exercise (see section 7.7)

*Range of movement assessments and circumferential swelling measurements must be conducted by a physiotherapist trained in the requirements of the study protocol and documented in the patient's record.

For peer review only - http://bmjopen.bmj.com/site/about/guidelines.xhtml

9.4.1. Provision of Patient Diary

Each participant will be provided with a second trial-specific Patient Diary to facilitate completion of questionnaires at the 10-week time point.

9.4.2. End of 4-week follow up visit procedures

A member of the research team will ensure the following before the patient leaves the hospital:

- A copy of the 5-week follow-up questionnaires (and a freepost envelope) will be given to participants. They will be instructed on how to complete the questionnaire using their preferred option (online, phone or post). The importance of completing the questionnaire within 3-10 days will be emphasised. Participants will be encouraged to complete the questionnaire online. If they would prefer not to use the online method, they will be encouraged to complete over the phone. Those participants, who state that they are unable to complete the questionnaire online or by telephone, will use postal completion. Participants who are completing questionnaires online will be emailed to access this approximately 3 days after their 4 week clinic appointment. Participants who have opted for telephone completion will be telephoned approximately 5 days after their 4 week clinic appointment. Participants will be encouraged to have the paper version of the questionnaire by their side during the phone call.
- The patient has their Patient Diary and is aware that they will be receiving questionnaires in approximately 6 weeks' time by post/email and that they may be contacted in the event that the questionnaires are not returned so as to prompt for return or to complete the questionnaire over the telephone.

The research team will also ensure that the patient's continuing participation in the trial is appropriately recorded in the hospital records in accordance with local practice.

Completed questionnaires and CRF pages will be sent to PenCTU using FREEPOST envelopes.

9.5. **5-week follow-up questionnaire completion**

The 5-week follow-up requires participants to complete a questionnaire comprising the following:

- Olerud and Molander (1984) ankle score (see section 7.1)
- EQ-5D-5L (see section 7.2)

The aim is to collect data on primary outcome and EQ-5D-5L at 5 days after the participants 4 week clinic visit. Most participants will have had their plaster / boot removed at the 4 week appointment. It is thought that any benefits resulting from use of the boot would be apparent at this time point. The time window for a valid response will be defined as 3-10 days after the 4 week appointment. The method of data collection has been designed to minimise the risk of missing data. Too much missing data may have a negative impact on the statistical analysis and

For peer review only - http://bmjopen.bmj.com/site/about/guidelines.xhtml

interpretation of results (e.g. less precision and possibility of bias). For ease of communication this follow-up will be referred to as the 5 week follow-up.

PenCTU staff will monitor receipt of questionnaires and will issue reminders to patients if the questionnaire has not been returned. If a questionnaire has not been received within 8 days from the week 4 visit, attempts will be made to contact the participant by telephone and the option of completing the questionnaire by telephone will be available.

9.6. 10-week follow-up (12 weeks post-surgery)

9.6.1. 10-week follow-up questionnaire completion

The 10-week follow-up requires participants to complete a questionnaire comprising the following:

- Olerud and Molander (1984) ankle score (see section 7.1)
- EQ-5D-5L (see section 7.2)
- Resource use questionnaire (see section 7.4)
- Return to activities (see section 7.2)

PenCTU staff will issue questionnaires to participants according to a participant schedule created in the trial database through registration of randomisation and 4-week follow-up dates. The information accompanying the questionnaire will remind participants to make use of their Patient Diaries when completing the questionnaires and request that they are returned within 7 days. The time window for a valid response will be defined as 28 days from the issue of the first week 10 questionnaire. If patients have a preference to receive the questionnaire by post, a self-addressed FREEPOST envelope will be included to facilitate postal return. Participants who have opted to complete questionnaires online will be emailed to access the questionnaire. PenCTU staff will monitor the sending and receipt of questionnaires and will issue another questionnaire with a reminder if the first one has not been returned within 10 days from issue. If a completed questionnaire has not been received by 14 days from issue of the first questionnaire, attempts will be made to contact the participant by telephone. Questionnaires may be completed over the telephone if online/postal questionnaires have not been returned or are lost - if participants feel unable to complete the full questionnaire, participants will have the option of completing a subset of the questionnaire booklet by telephone. Priority will be given to collecting the Olerud & Molander ankle score and EQ-5D-5L.

9.6.2. 10-week follow-up CRF completion

Secondary care healthcare resource use information within the 10 week trial period will be collected and transcribed from the hospital records into the CRF by the local research team. Data extracted will include hospital admissions, outpatient visits, including physiotherapy sessions, day case procedures, visits to Accidents & Emergency departments and diagnostic tests or investigations conducted outside the outpatient visit tariff (See Section 7.4 Resource Use).

A small proportion of patients with complicated recoveries may need further clinical evaluation at 12-weeks post-surgery, as per standard care. The research team will

For peer review only

record the date when the patient was discharged or whether they returned to clinic for further follow up at 10 weeks post baseline (12 weeks-post surgery).

Completed CRF pages will be sent to PenCTU using FREEPOST envelopes.

9.7. Telephone interviews

At approximately ten weeks post-baseline, telephone interviews will be conducted by the trial qualitative researcher with up to 20 participants (10 from each treatment group) in order to collect information on patients' experience of their treatment, including psychological and social impact. Telephone interviews will last up to 60 minutes and will be recorded digitally. Further details and description of proposed analysis is given in section 12.3 (Qualitative component).

9.8. Radiographic Evaluation

Pre-operative, intra-operative and post-operative X-rays will be taken for each participant as per routine care. In some cases, particularly where recovery is complicated, further X-rays within the 12-week trial period may also be taken. At the end of the study, pre-, intra- and post-operative X-rays, plus any further X-rays taken within the trial period will be reviewed by the CI in a blinded fashion on a participant-by-participant basis. For each participant, the CI will record the following on a bespoke data collection form:

- Fracture classification (Weber A, B or C)
- Fracture pattern (medial malleolar fracture or deltoid ligament injury)
- Integrity of surgical metalwork (displaced or undisplaced)
- Evidence of post-operative fracture healing (yes/no)

The data collection forms will be returned to the CTU for entry into the trial database. Research teams at local sites will liaise with their X-ray departments to arrange anonymisation and transfer of images. Instructions of procedures by which X-rays are collected, labelled, stored and sent are detailed in a separate work instruction.

10. INTERVENTION DETAILS

10.1. Removable support boot

Patients in the boot group will be fitted with a removable support boot/pneumatic walker brace. To ensure the trial is pragmatic, any model of boot may be used, providing it meets the following specifications:

- Has at least two air cells to provide compression
- Rigid (e.g. plastic) anterior and posterior outer sections. NB: Boots with soft (e.g. foam only) anterior sections are not suitable as they offer reduced support to the ankle
- Suitable for weight-bearing and removable to allow for mobilisation of the ankle

For peer review only - http://bmjopen.bmj.com/site/about/guidelines.xhtml

The CI and Lead Site Research Physiotherapist will assess the specifications of support boots prior to their use in the trial to ensure their suitability to deliver the trial intervention.

Patients in the boot group will be given a trial specific advice leaflet advising regular exercise by a physiotherapist trained in the requirements of the protocol e.g. regular removal for exercise during the day/boot off at night as tolerated. The leaflet will provide illustrated instructions for completion of the following exercises; Straight leg raise, Ankle dorsiflexion/plantarflexion, Ankle inversion/eversion, Calf stretch, and Resisted plantarflexion. Patients will be encouraged to mobilise their ankle as often in a day as pain allows (recommended at least 3 times a day.)
Participants will be advised on gait re-education with crutches and will progress to weight-bearing as able.

10.2. Plaster Cast

The plaster cast used will be a standard below knee rigid plaster cast shell with soft lining to provide support in a fixed position to the ankle. The plaster cast is rigid enough to support weight-bearing and is custom built for the patient.

The plaster group will also receive a trial specific advice leaflet on plaster care/mobilisation of free joints. The leaflet will provide illustrated instructions of the following exercises: Toe flexion/extension, Static quads and Straight leg raises. Patients will be encouraged to mobilise their ankle as often in a day as pain allows (recommended at least 3 times a day.)
Participants will be advised on gait re-education with crutches and will progress to weight-bearing as able.

10.3. Associated Risks

The risks associated with participating in the ART study are no different to the risks of receiving standard treatment outside the study.
However, known complications include: blisters, pressure sores, failure of fixation, operation/revision surgery, wound infections, compartment syndrome, deep vein thrombosis (DVT), nerve injury, wound breakdown, and pulmonary embolism.

Participants randomised to plaster may experience more pressure sores, stiffness, difficulty accessing and monitoring wounds and similarly, participants in a support boot may experience problematic wounds.

11. ASSESSMENT OF SAFETY

11.1. Standard definitions

Adverse events (AE) are defined as any untoward medical occurrence in a clinical trial subject and which do not necessarily have a causal relationship with the treatment.

Adverse reactions (AR) are defined as an event that is considered to have a suspected causal relationship to the trial intervention (plaster or support boot.)

For peer review only

Serious adverse events (SAE) are defined as any untoward and unexpected medical occurrence that:

- Results in death
- Is life-threatening[1]
- Requires hospitalisation or prolongation of existing inpatients´ hospitalisation,
- Results in persistent or significant disability or incapacity
- Is a congenital anomaly or birth defect

11.2. Reportable events

In the ART trial, all SAEs (as defined above) will be reported to PenCTU regardless of causality. Events which are not serious will not be collected or reported as adverse events but complications listed in section 10.3 will be reported as outcome measures.

11.3. Procedures for Serious Adverse Event reporting

Procedures for collecting and reporting SAEs will be described in a separate trial-specific Work Instruction and will be discussed during investigator site initiation meetings.

SAEs may be identified via patient report at clinic visits or from receipt of resource use questionnaires which indicate a hospital admission. All SAEs will be reported to CTU using a bespoke Serious Adverse Event reporting form (SAE form). PIs or delegated staff must fax/email the completed SAE form to CTU within 24 hours of becoming aware of the event. The SAE report form will include a description of the event and an assessment of severity and causality as described below. PenCTU will maintain a register of all reported SAEs.

11.3.1. Assessment of severity and causality

All SAEs must be assessed for severity by the PI(s) or by those members of the research team specifically authorised by the Principal Investigator(s) to assess SAEs, as documented in the site delegation log. Assessments of severity will be made according to the following conventions:

Mild:	An event that is easily tolerated by the patient, causing minimal discomfort and not interfering with every day activities
Moderate:	An event that is sufficiently discomforting to interfere with normal everyday activities
Severe:	An event that prevents normal everyday activities

The term ‘severe’, used to describe the intensity of an event or reaction, should not be confused with the term ‘serious’ which is a regulatory definition. For example, a headache may be severe but not serious, while a minor stroke may be serious but is not severe.

---

[1] Life-threatening in the definition of a serious adverse event or serious adverse reaction refers to an event in which the subject was at risk of death at the time of event; it does not refer to an event which hypothetically might have caused death if it were more severe.[20]

For peer review only

For peer review only - http://bmjopen.bmj.com/site/about/guidelines.xhtml

An assessment of the causal relationship between an SAE and trial participation will be made according to the standardised guidance given in Table 2 below:

For peer review only

For peer review only - http://bmjopen.bmj.com/site/about/guidelines.xhtml

Table 2: Definitions of Causality

Relationship	***Description***
Unrelated	*There is no evidence of any causal relationship*
Unlikely	*There is little evidence to suggest there is a causal relationship (e.g. The event did not occur within a reasonable time after administration of the trial treatment/procedure). There is another reasonable explanation for the event (e.g. The participant's clinical condition, other concomitant treatment).*
Possible	*There is some evidence to suggest a causal relationship (e.g. Because the event occurs within a reasonable time after administration of the trial treatment/procedure). However, the influence of other factors may have contributed to the event (e.g. The participant's clinical condition, other concomitant treatments).*
Probable	*There is evidence to suggest a causal relationship and the influence of other factors is unlikely.*
Definitely	*There is clear evidence to suggest a causal relationship and other possible contributing factors can be ruled out.*

11.3.2. Assessment of expectedness

SAEs deemed to be possibly, probably or definitely related to trial participation will be classified as SARs. In such cases, the PI or authorised delegate will make a judgement on whether the event is expected or unexpected. The assessment of expectedness will be made with reference to the known risks listed in section 10.3. An event will be classed as unexpected if it is not listed in protocol section 10.3.

11.4. Processing Serious Adverse Event report forms

On receipt of a completed SAE form, PenCTU will assign a unique SAE number and confirm receipt of the event to the reporting site and to the study Sponsor. Staff from the reporting site will file the receipt confirmation and the original SAE report form in the relevant participants CRF. Any further correspondence relating to the same event (e.g. data query forms) must also be filed with the original form.

If incomplete information is available at the time of reporting, all appropriate information relating to the SAE will be forwarded to PenCTU as soon as possible. All serious adverse events will be followed up until resolution.

11.4.1. Onward reporting

SAEs will be reported on a periodic basis to the Sponsor, CI and Trial Steering Committee in order to maintain sufficient oversight of trial safety.

SAEs deemed related to the trial procedures and which are unexpected are subject to expedited reporting; they will be notified to the Research Ethics Committee (REC) within 15 days for a non-life threatening event and within 7 days for a life-threatening event. Such events will be reported to the Trial Steering Committee on an ongoing basis.

For peer review only - http://bmjopen.bmj.com/site/about/guidelines.xhtml

12. STATISTICS AND DATA ANALYSIS

12.1. Sample Size Calculation

The study is powered to detect a 10-point difference in ankle score since patient and public involvement indicated that walking without aids/getting back to work were the most important issues. A change from support with a stick/crutch to no support and a change to getting back to normal work/activities of daily life from simpler or part-time work are both associated with a 10-point change on the Olerud and Molander[14] subjective scale (primary outcome).

An estimate of standard deviation of the ankle score six weeks post-surgery was derived from an audit of 18 patients having plaster casts at Poole Hospital. This figure of 21.9 is very similar in magnitude to those reported in the Cochrane review[13]. Based on an unpaired t-test with two groups of equal size, assuming 90% power, a two-sided 0.05 significance level, a standard deviation of 21.9 and a mean difference of 10 points between the plaster and boot groups, a total sample size of 204 (102 in each group) is required[21]. The study will over-recruit by 20%[22] to allow for non-responders, or missing data, not to contribute data to the main analysis. However it is anticipated that the true percentage will be reduced by offering flexible methods of completion; either online, post or by phone. As the study progresses, the percentage with missing primary outcome data will be monitored and discussed at regular trial management group meetings and reductions/ increases in over-recruitment will be made accordingly so that the target of 204 with completed data is met.

The study will aim to recruit 246 patients in total (123 in each group) over a 2 year recruitment period.

12.2. Description of statistical methods

A detailed Statistical Analysis Plan will be developed and signed off by the Trial Steering Committee before any analyses commence. The anonymised data will be analysed by ‘intention to treat’ (i.e. patients will be analysed in the treatment arm they were randomised to, even if they received something else, or swapped treatments). The analysis and initial write-up will be blinded to the treatment groups and will report according to the Consolidated Standards of Reporting Trials[23-24], including a participant flow chart. No imputation methods for missing data will be used for the main effectiveness analyses.

12.2.1. Baseline descriptives:

Baseline descriptive data on demographics (gender, age, marital status, whether living alone, highest educational level, employment status, driving status), fracture characteristics (fracture classification, pattern, complexity), BMI, Olerud and Molander ankle score pre-injury, and EQ-5D will be presented overall and for both groups separately. This will help with (a) assessment of external validity of the trial, and (b) to see whether the 2 groups were comparable at baseline (no significance tests will be conducted). The Olerud and Molander scale includes a question on activity compared to before injury. This would make this question confusing to complete at baseline, and so for baseline only this question has been dropped and

For peer review only - http://bmjopen.bmj.com/site/about/guidelines.xhtml

all participants will be given the maximum score of 20 for this question (i.e. the score for work and activities same as before injury).

12.2.2. Primary outcome:

The primary outcome is the Olerud and Molander[14] ankle score (measured out of 100) five weeks post-baseline. Questionnaires that were not completed in the 3-10 day time window will be treated as missing data.

Multiple regression including study site, age and fracture complexity as a factor will be used to compare mean ankle score at five weeks post-baseline between the plaster and boot groups.
Instead of implementing stratification of age and fracture complexity at randomisation (which in any case could be problematic if there is a large number of strata with a relatively small sample size), the potential issue of imbalance in age and fracture complexity between the two arms of the trial will be addressed by adjusting (stratifying) for the covariates of age (as a continuous variable) and fracture complexity in the analysis. Thus the proposed analysis of the primary outcome, for example, will use multiple regression and, in addition to treatment arm, include the pre-specified variables of study site, age and fracture complexity.

Two groups are proposed regarding fracture complexity: Simple fibular fracture and comminuted fracture. If the fibular fracture has a single fracture line with two discrete fragments only, it comprises a 'simple' fracture. If the fibula has more than one fracture line and therefore more than two discrete fragments that require accurate reduction to achieve stability, it comprises a comminuted fracture. The pattern and complexity of the fracture can influence the choice of hardware used by the surgeon to achieve stability. Simple fractures are fixed with unlocked plates whereas complex fractures can require more sophisticated and expensive locked plates.

The pre-baseline measure is collected retrospectively and so has not been included in the primary analysis. By ten weeks post-baseline, it is anticipated that both groups will be similar on the primary outcome, and so this has not been included in an overall repeated measures type analysis. The five week post-baseline data collection period is the critical period, as this is when it is anticipated that the group who had boots will have a greater level of function[3,6].

Some additional analyses (including sensitivity analyses) on primary outcome will also be conducted:
(a) It is acknowledged that some participants (in either group) will leave the 4 week appointment with a plaster cast or boot, and so will complete the scale whilst still wearing the plaster/ boot. In a supplementary sensitivity analysis these participants will be excluded from the analysis to see if the results change.
(b) Pre-injury Olerud and Molander scale will be included as a covariate.
(c) Whether or not the patient had a medial malleolar fracture will be included as a covariate
(d) Any baseline variables that appear, by chance, to differ between the groups will be added in as covariates.
(e) The effectiveness of the intervention may vary across different subgroups (strata) of patients (e.g. age and fracture complexity). Therefore for each outcome

For peer review only - http://bmjopen.bmj.com/site/about/guidelines.xhtml

supplementary statistical analyses are proposed in which it is tested whether there is a statistical interaction between age and treatment arm and between fracture complexity and treatment arm. It is however acknowledged that statistical power will be reduced for these significance tests and they will be considered to be exploratory. Age will be grouped as: under 65 years of age at the time of fracture and 65 + years.

(f) The main analysis will use an "intention to treat" approach. As a supplementary analysis two sets of "per protocol" analyses will also be conducted. In the first of these, patients will be analysed according to the method (boot or plaster) that they actually wore. If patients crossed over from one to the other during the 4 week period they will be excluded from the per protocol analysis. If patients didn't wear the boot/ plaster for the full 4 weeks they will be excluded. In the second analysis participants will additionally be excluded if they didn't perform the exercises recommended. Thus if they performed the exercises less than once per day they will be excluded

(g) Data on primary outcome completed outside of the 3-10 day window will be included in the analysis

(h) The robustness of the results to missing data will be assessed by repeating the analysis incorporating the imputed values that will be derived as part of the economic analysis (see section 12.4)

The study will also compare the ten week Olerud and Molander Ankle score between groups to check whether any differences still remain at 10 weeks.

To see whether patients in both groups have returned to pre-injury levels of function we will calculate the mean difference (95% CI) between pre-injury and 10 week values for each group, and the proportion of patients whose score is worse by 10 points or more. These statistics will also be compared between groups.

12.2.3. Secondary outcomes:

The profiles from the patients' answers to the EQ-5D-5L will be weighted using the EuroQol's published United Kingdom societal utility tariffs to produce a composite, utility based quality of life score and EuroQol has piloted the 5L valuation scores[25]. If final scores are not available by the end of the trial, the crosswalk developed by EuroQol from the three level scores will be used. Quality Adjusted Life Years will then be created from the four time point utility scores assuming a linear change between the time points and using the area under the curve approach. Incremental QALY gains between arms will then be estimated using regression analysis adjusted for baseline utility and site, age and fracture complexity variables.

Multiple regression will be used to investigate differences between the two groups in the other continuous outcomes measured at four weeks post-baseline (e.g. degrees of range of movement (dorsiflexion, plantarflexion, inversion, eversion), circumferential swelling and 10 weeks (e.g. return to daily activities on 0-10 scale). Complications in the two groups (presence and severity), healing status (displaced / undisplaced and evidence of post-operative fracture healing (yes/no)), employment status at 10 weeks, driving status and weight bearing status will be investigated using logistic regression (binomial or multinomial depending on the number of categories), again taking study site, age and fracture complexity into account. Survival analysis will be used to investigate the time from randomisation to starting to drive again. Supplementary analysis (b) to (e) as described for the primary outcome will also be conducted for secondary outcomes.)

For peer review only - http://bmjopen.bmj.com/site/about/guidelines.xhtml

12.3. Qualitative Component

At approximately ten weeks post-baseline, telephone interviews will be carried out with up to 20 participants or until data saturation. A maximum variation strategy will be used to capture a range of experiences of participants receiving each treatment. Telephone interviews will last up to 60 minutes, will be recorded digitally, and were chosen to reduce costs and for convenience to the patients. The topic guide has been developed through collaboration with service users, and addresses:

- Experiences of each type of treatment (plaster/boot).
- Lifestyle implications – individual (e.g. washing), family (including caregivers, as requested by feedback from patient and public involvement), social, psychological, work, financial, residential, transport (e.g. driving), diet, leisure activities, and independence.
- Expectations, mind-set, confidence, including the perceived views of their carers as suggested by the patient and public involvement.

The telephone interviews will be transcribed verbatim, anonymised, and coded using Thematic Analysis[26]. A second researcher will analyse a selection of transcripts to validate the codebook/coding. It is anticipated that the findings from this qualitative study will enhance the results by explaining the psychological and social impact of each treatment.

12.4. Economic Evaluation

The primary economic evaluation will compare the costs and health benefits of the support boot compared to plaster in short-term ankle fracture management from an NHS and Personal Social Services perspective. In sensitivity analysis, an economic evaluation from a societal perspective on costs will be performed.

The evaluation will be:

1. A cost-utility analysis at ten weeks post-randomisation; Quality Adjusted Life Years (QALYs) between arms will be compared to identify whether any treatment arm is dominant (i.e. less costly and more effective). If no arm is dominant, results will be presented as a bootstrapped incremental cost-effectiveness ratio (ICERs) with respect to QALYs gained. Bootstrapped estimates of costs and effects will be plotted in the cost-effectiveness plane. Incremental net monetary benefit statistics (INMB) will be derived using the £20,000 and £30,000 societal willingness to pay thresholds for a QALY. Cost-Effectiveness Acceptability Curves (CEACs) will be plotted to show the probability of the intervention being cost-effective at a range of societal willingness to pay thresholds, thus addressing uncertainty around the adoption decision. If one arm is dominant, ICERs are not meaningful and we will only report INMB statistics and plot the cost-effectiveness planes and CEAC.

2. A cost-effectiveness analysis at approximately 5 weeks post-baseline, coinciding with the collection of primary clinical outcome; Costs and Olerud and Molander scores between arms will be compared to identify whether any treatment arm is dominant. If no arm is dominant, results will be presented as a bootstrapped incremental cost-effectiveness ratio with respect to the Olerud and Molander[14] scale. If the treatment arm is a dominant or dominated strategy, ICERs will be

For peer review only - http://bmjopen.bmj.com/site/about/guidelines.xhtml

negative and not meaningful. In that case, we will only report INMB statistics in relation to QALYs and will not proceed any further with the cost-effectiveness analysis with respect to the Olerud and Molander scale.

Collecting 5 week and 10 week resource use:

Resource use collection will take place as defined in above section “7.4 Resource Use” in this protocol.

At 10 weeks, resource use for the primary economic result will result from adding secondary care resource use collected from the review of 10 weeks of medical notes and the two resource use questionnaires at 4 weeks and 10 weeks.

For the second economic result, the primary clinical outcome collected at approximately 5 weeks (4 weeks + 3-10 days) after randomisation will be compared with costs for the same period. We will identify the proportion of secondary care cost, collected from medical notes, taking place in the additional days post the 4 week clinic visit for the patient, and assume the patient would have an equal proportion of cost for the other cost categories, collected by questionnaire. This assumed incremental patient reported cost, will be added to the 4 week cost data collected from questionnaires and the corresponding period cost from medical notes, to compose a total patient cost from baseline to collection of primary clinical outcome data.

Valuing resources:

Resource use will be valued using unit costs for health and social care, department of health reference costs or other national routine sources[27-30]whenever available. Trust finance departments will be contacted for procurement costs on the boot and plaster materials. Productivity losses will measure absenteeism and presenteeism and be valued using the human capital approach[31-32] in the main analysis, and the friction costs approach in sensitivity analysis. Estimations will be adjusted for pre-randomisation work status and return to work.

Analysis of costs and outcomes:

Costs and QALYs (mean and confidence intervals) will be estimated using regression analysis, adjusting for site, age, and fracture complexity in addition to baseline utility for QALYs). We will ascertain whether the treatment arm would be dominant, i.e. less costly and more effective, or dominated, i.e. more costly and less effective, with respect to QALYs and the Olerud and Molander scale.

Missing data in the economic result:

Cost is cumulative and additive: missing data in one cost component at one follow-up time point means that the full cost per patient cannot be computed. A specific missing data strategy has been developed for the economic evaluation, in case of poor patient-completion of resource use questions.

Missing data will be imputed using multiple imputation methods, in a two-stage ordinary least squares model, adjusting for socio-demographic characteristics

For peer review only - http://bmjopen.bmj.com/site/about/guidelines.xhtml

collected at baseline. As the primary economic result are parameters (ICERs / NMB) relating Costs and QALYs, the imputation models will simultaneous include disaggregated cost categories and utilities.

If less than 30% of the cost and QALY data is missing, the main economic analyses will report the complete case scenario. If more than 30% of data is missing, the main economic analysis will report the primary economic result using the completed imputed dataset, with the complete case scenario as the secondary analysis. Should the secondary analysis using the primary clinical outcome require imputation, imputed value sets from the statistical analysis will be used to estimate the economic result parameters.

Sensitivity analysis:

One way and scenario sensitivity analysis will be used to address uncertainty of costing and methodological assumptions. These may include, for example, adding a societal perspective to the primary NHS+PSS (prescribed specialised services) result, valuing productivity losses using a different approach (i.e. friction cost), and costing assumptions for the 3 to 10 days incremental cost period not directly captured in questionnaires. Results will be presented disaggregated by perspective (i.e. National Health Service/patients/societal) for clear decision-making information.

13. ETHICAL, REGULATORY, ADMINISTRATIVE AND QUALITY ASSURANCE

13.1. Ethical considerations

This trial was designed around the patient pathway and the usual management of ankle fractures within National Health Service Trusts, and therefore raises no special ethical issues. Staff will be trained in Good Clinical Practice which will be followed at all times.

An application for ethics approval will be made to a National Research Ethics Service Committee (REC). The trial will not proceed until Ethics approval and Trust approval from each participating site is obtained. Any amendments to the protocol will be submitted for REC approval and local NHS approval as appropriate.

Annual progress reports will be submitted to the REC using the recognised National Research Ethics Service (NRES) template. An end-of-trial declaration will be provided to the REC within 90 days of trial conclusion or within 15 days of trial termination in the event the trial is prematurely terminated.

Since the trial involves no Investigational Medicinal Product or non-CE marked medical devices, authorisation from the Medicines and Healthcare products Regulatory Agency will not be sought.

For peer review only

For peer review only - http://bmjopen.bmj.com/site/about/guidelines.xhtml

13.2. Declaration of Helsinki

The trial will be conducted in accordance with the ethical principles that have their origin in the Declaration of Helsinki, and that are consistent with GCP.

13.3. Research Governance

This trial will adhere to the principles outlined in the International Conference on Harmonisation Good Clinical Practice (ICH GCP) guidelines. It will be conducted in compliance with the protocol, NHS Research Governance Framework for Health and Social Care (2005 2nd edition) and other regulatory requirements as appropriate.

13.4. Study Management

The Trial Manager will co-ordinate the study (overseen by the Chief Investigator, supported by the Bournemouth University Clinical Research Unit and Peninsula Clinical Trials Unit). The Peninsula Clinical Trials Unit is a United Kingdom Clinical Research Collaboration registered Clinical Trials Unit and will conduct randomisation and manage the data following their Standard Operating Procedures.

Bournemouth University will support both the quantitative and qualitative analysis, the University of Bristol will support economic analysis, and Peninsula Clinical Trials Unit will provide randomisation, data management services and senior trial management support. The CI will be responsible for the overall running of the trial and will be in regular contact with PIs at the other participating sites.

The Trial Management Group (TMG) chaired by the Chief Investigator, will include all co-applicants, Trial Manager and service user representatives. The TMG will take responsibility for monitoring progress, ensuring development of documentation and forms, monitoring participant recruitment, follow-up and budget, discussing analysis, results, draft reports and dissemination. The TMG will meet regularly prior to the start of the study, and then in person every six months and remotely in between. There will be no Data Monitoring Committee (DMC) since both treatments are used in clinical practice.

13.5. Trial Steering Committee

The Trial Steering Committee (TSC) is responsible for the overall supervision of the trial, including overseeing recruitment, ensuring milestones are achieved and general scientific probity is maintained. In the absence of the DMC, the TSC will take on the role of reviewing adverse events, maintaining safety and will make recommendations for the premature discontinuation of the study where necessary.

The TSC will be chaired independently and will comprise of 6 full members; 3 independent experts (clinician(s)/statistician(s)), the Chief Investigator, and a service user representative. The Trial managers, data manager, sponsor and a funder representative will be invited to TSC meetings as observers. Members will meet at least annually as part of the Trial Steering Committee and will follow functions according to the Medical Research Council Good Clinical Practice guidelines[33]. The composition and function will be described in a TSC charter prepared prior to study commencement.

For peer review only - http://bmjopen.bmj.com/site/about/guidelines.xhtml

13.6. Monitoring

Continuous data monitoring will be undertaken by the study team. Monitoring visits to all sites will also be conducted to check protocol compliance during the study, and as required where problems/potential problems are identified. The frequency and extent of monitoring visits will be described in a monitoring plan, the first version of which will be finalised prior to participant recruitment. Visits will be carried out by the Trial Manager and/or the Sponsor's quality assurance staff. Relevant trial-related documents will be made available by the PIs and research team for monitoring.

13.7. Audit and Inspection

The investigator(s)/ institution(s) will permit trial-related monitoring, audits, REC review, and regulatory inspection(s), providing direct access to source data and essential documents. Trial participants are informed of this during the informed consent discussion. Participants will consent to provide access to their medical notes.
In the event of the site being notified directly of a regulatory inspection, the sponsor requests the investigator to notify the sponsor.

14. DATA HANDLING AND RECORD KEEPING

14.1. Participant Numbering

Upon randomisation, each participant will be allocated a unique trial number and will be identified in study documentation by his/her trial number and initials.

14.2. Source data

The first record of any study related visit or assessment should be recorded in the patient's medical record or patient questionnaire, which becomes the source data. Data from source documents will be transcribed into study specific CRFs by authorised personnel on the delegation log. All X-ray images will be anonymised and transferred securely via The Image Exchange Portal (IEP) or by anonymised CD.

14.3. Data Entry

Data will be recorded on study-specific case report forms (CRFs). Completed CRFs will be sent to the PenCTU for double-data entry on to a password-protected database accessed via the Internet. Forms will be tracked using a web-based trial management system. Double-entered data will be compared for discrepancies using a stored procedure. Discrepant data will be verified using the original paper data sheets. The data manager will also coordinate the sending, tracking, receipt and entry of patient questionnaires.

Anonymised socio-demographic, outcome and resource use data will be shared with Bournemouth University and the University of Bristol for analysis to be performed.

14.4. Data confidentiality and security

Investigators will ensure that the patients' anonymity is maintained on all documents. Data will be collected and stored in accordance with the Data Protection Act 1998.

For peer review only - http://bmjopen.bmj.com/site/about/guidelines.xhtml

PenCTU will store anonymised study data in locked filing cabinets within a locked office. Electronic records will be stored in a SQL server database, stored on a restricted access, secure server maintained by the University of Plymouth. The website will be encrypted using SSL. Direct access to the trial data will be restricted to members of the research team and the CTU, with access granted to the Sponsor on request. Copies of original study data retained at study sites will be securely stored for the duration of the study prior to archiving.

14.5. Archiving

Following completion of trial data analysis, data and essential documentation will be archived in a secure location for at least five years after the end of the trial, in accordance with the Sponsor's standard operating procedure. No trial-related records should be destroyed unless or until the Sponsor gives authorisation to do so.

15. FINANCE, INDEMNITY AND INSURANCE

15.1. Funding

The study will be funded by the NIHR Research for Patient Benefit (RfPB) Grant, reference number; PB-PG-0213-30021. Poole Hospital will centrally manage the financial arrangements in accordance with National Institute for Health Research, Research for Patient Benefit and Trust requirements.

15.2. Sponsor

The study is sponsored and coordinated by Poole Hospital NHS Foundation Trust.

15.3. Indemnity

Poole Hospital NHS Foundation Trust holds standard NHS Hospital Indemnity and insurance cover with NHS Litigation Authority for NHS Trusts in England. Standard NHS cover for negligent harm is in place. There are no specific arrangements to cover for non-negligent harm.

16. PUBLICATION POLICY AND INTELLECTUAL PROPERTY

16.1. Publication Policy

The results of the study will be disseminated via presentations at appropriate scientific meetings and conferences and publication in appropriate peer-reviewed journals. The Trial Management Group will produce a publication policy which will outline the intended outputs, authorship and contribution, target outlets.

If the participants would like to be informed of the results of the study, they can be sent a plain English summary of the study results after the end of the study.

16.2. Intellectual Property

Arrangements with collaborating sites will be in accordance with the relevant clauses from the NIHR standard template for non-commercial studies. Intellectual Property rights will be described in agreements between the sponsor and collaborating institutions.

For peer review only - http://bmjopen.bmj.com/site/about/guidelines.xhtml

17. REFERENCES

1. Donaldson LJ, Reckless IP, Scholes S, Mindell JS, Shelton NJ. The epidemiology of fractures in England. *Journal of Epidemiology and Community Health* 2008;62(2): 174-180.

2. Lloyd J, Lockhart J, Sisak K, Middleton R. How to cut overnight stays and improve trauma pathways. *Health Service Journal* 2012. http://m.hsj.co.uk/5041062.article (accessed 11 January 2013).

3. Thomas G, Whalley H, Modi C. Early mobilization of operatively fixed ankle fractures: a systematic review. *Foot and Ankle International* 2009;30(7): 666-674.

4. Shaffer MA, Okereke E, Esterhai JL Jr, Elliott MA, Walker GA, Yim SH, Vandenborne K. Effects of immobilization on plantar-flexion torque, fatigue resistance, and functional ability following an ankle fracture. *Physical Therapy* 2000;80(8): 769–780.

5. Stevens JE, Walter GA, Okereke E, Scarborough MT, Esterhai JL, George SZ, Kelley MJ, Tillman SM, Gibbs JD, Eilliot MA, Frimel TN, Gibbs CP, Vandenborne K. Muscle adaptations with immobilization and rehabilitation after ankle fracture. *Medicine and Science in Sports and Exercise* 2004;30(10): 1695–1701.

6. Vioreanu M, Dudeney S, Hurson B, Kelly E, O'Rourke K, Quinlan W. Early mobilisation in removable cast compared with immobilisation in a cast after operative treatment of ankle fractures: a prospective randomised study. *Foot and Ankle International* 2007;28(1): 13-18.

7. Simanski C, Maegele M, Lefering R, Lehnen D, Kawel N, Riess P, Yücel N, Tiling T, Bouillion B. Functional treatment and early weight-bearing after an ankle fracture; a prospective study. *Journal of Orthopaedic Trauma* 2006;20(2): 108-114.

8. Dogra AS, Rangan A. Early mobilisation versus immobilisation of surgically treated ankle fractures. Prospective randomised control trial. *Injury* 1999;30(6): 417-419.

9. Lehtonen H, Jarvinen T, Honkonen S, Nyman M, Vihtonen K, Jarvinen M. Use of a cast compared with a functional ankle brace after operative treatment of an ankle fracture. A prospective, randomized study. *Journal of Bone and Joint Surgery* 2003;85(2): 205-211.

10. Farsetti P, Caterini R, Potenza V, De Luna V, De Maio F, Ippolito E. Immediate continuous passive motion after internal fixation of an ankle fracture. *Journal of Orthopaedics and Traumatology* 2009;10(2): 63-69.

11. ISRCTN Register. *A trial comparing weight-bearing to non-weight-bearing following ankle fracture fixation.*

For peer review only - http://bmjopen.bmj.com/site/about/guidelines.xhtml

http://www.controlledtrials.com/mrct/trial/printfriendly/2293127 (accessed 11 January 2013).

12. US National Institutes of Health. *Early Weight-bearing and Mobilization versus Non-Weight-bearing and Mobilization in Unstable Ankle Fractures.* http://clinicaltrials.gov/ct2/show/nct01196338 (accessed 11 January 2013).

13. Lin CW, Moseley AM, Refshauge KM. Effects of rehabilitation after ankle fracture: a Cochrane systematic review. *European Journal of Physical and Rehabilitation Medicine* 2009;45(3): 431-441.

14. Olerud C, Molander H. A scoring scale for symptom evaluation after ankle fracture. *Archives of Orthopaedic and Traumatic Surgery* 1984;103(3): 190-194.

15. Rabin R, Oemar M, Oppe M, Janssen B, Herdman M *EQ-5D-5L User Guide. Basic Information on How to Use the EQ-5D-5L Instrument.* Version 1.0. EuroQol Group Executive Office on behalf of the EuroQol Group. Rotterdam: EuroQol Group; 2011. http://www.euroqol.org/fileadmin/user_upload/Documenten/PDF/Folders_Flyers/ UserGuide_EQ-5D-5L.pdf (accessed 31 October 2011).

16. Reilly Associates. *WPAI: GH V2.0.* http://www.reillyassociates.net/WPAI_GH.html (accessed 04 December 2012).

17. Zhang W, Bansback N, Boonen A, Young A, Singh A, Anis AH. Validity of the work productivity and activity impairment questionnaire – general health version in patients with rheumatoid arthritis. *Arthritis Research and Therapy* 2010;12(5): R177.

18. Braakman-Jansen LMA, Taal E, Kuper IH, van de Laar MAFJ. Productivity loss due to absenteeism and presenteeism by different instruments in patients with RA and subjects without RA. *Rheumatology* 2012;51(2): 354-361.

19. Weber BG. *Die Verletzungen Des Oberen Sprunggelenkes.* Second edition. Berne: Verlag Hans Huber; 1972.

20. European Commission, Detailed guidance on the collection, verification and presentation on adverse reaction reports arising from clinical trials of medicinal products for human use, ENTERPRISE AND INDUSTRY DIRECTORATE-GENERAL, May 2006, http://ec.europa.eu/health/files/eudralex/vol-10/21_susar_rev2_2006_04_11_en.pdf (accessed 10 June 2014.)

21. Faul F, Erdfelder E, Lang A-G, Buchner A. G*Power 3: A flexible statistical power analysis program for the social, behavioral, and biomedical sciences. *Behavior Research Methods* 2007;39(2): 175-191.

For peer review only - http://bmjopen.bmj.com/site/about/guidelines.xhtml

22. Sackett DL, Straus SE, Scott Richardson W, Rosenberg W, Haynes RB. *Evidence-Based Medicine. How to Practice and Teach EBM.* Second Edition. Edinburgh: Churchill Livingstone, Harcourt Publishers Limited; 2000.

23. Moher D, Hopewell S, Schultz KF, Montori V, Gotzsche PC, Devereaux PJ, Elbourne D, Egger, M, Altman DG. CONSORT 2010 Explanation and Elaboration: updated guidelines for reporting parallel group randomised trials. *British Medical Journal* 2010;340: e1-28.

24. Schulz KF, Altman DG, Moher D for the CONSORT Group. CONSORT 2010 Statement: updated guidelines for reporting parallel group randomised trials. *British Medical Journal* 2010;340: 698-702.

25. Herdman M, Gudex C, Lloyd A, Janssen M, Kind P, Parkin D, Bonsel G, Badia X. Development and preliminary testing of the new five-level version of EQ-5D (EQ- 5D-5L). *Quality of Life Research* 2011;20(10): 1727-1736.

26. Braun V, Clarke V. Using thematic analysis in psychology. *Qualitative Research in Psychology* 2006;3(2): 77-101.

27. Curtis L. *Unit Costs of Health & Social Care 2011*. Canterbury: Personal Social Services Research Unit; 2011. http://www.pssru.ac.uk/pdf/uc/uc2011/uc2011.pdf (accessed 16 January 2013).

28. Department of Health. *NHS 2010-11 Reference Costs Publication;* 2011. http://www.dh.gov.uk/prod_consum_dh/groups/dh_digitalassets/documents/digitalasset/dh_131160.pdf (accessed 11 January 2013).

29. British Medical Association, Royal Pharmaceutical Society of Great Britain, Joint Formulary Committee. *British National Formulary*. 62nd Edition. London: Pharmaceutical Press; 2011.

30. Office for National Statistics. *Average Weekly Earnings Dataset* 2011. http://www.ons.gov.uk/ons/rel/lms/labour-market-statistics/october-2011/earnings-tsd-dataset.html (accessed 11 January 2013).

31. Koopmanschap MA, Rutten FF, van Ineveld BM, van Roijen L. The friction cost method for measuring indirect costs of disease. *Journal of Health Economics* 1995;14(2): 171-189.

32. van den Hout WB. The value of productivity: human-capital versus friction-cost method. *Annals of the Rheumatic Diseases* 2010;69(Supplement I): i89-i91.

33. Medical Research Council. *MRC guidelines for good clinical practice in clinical trials.* MRC Clinical Trials Series 2010. http://www.mrc.ac.uk/Utilities/Documentrecord/index.htm?d=MRC002416 (accessed 11 January 2013).

For peer review only

For peer review only - http://bmjopen.bmj.com/site/about/guidelines.xhtml

BMJ Open

Use of removable support boot vs cast for early mobilisation after ankle fracture surgery: Cost-effectiveness analysis and qualitative findings of the Ankle Recovery Trial. (ART)

Journal:	*BMJ Open*
Manuscript ID	bmjopen-2023-073542.R1
Article Type:	Original research
Date Submitted by the Author:	05-Oct-2023

Complete List of Authors:	Baji, Petra; University of Bristol; Corvinus University of Budapest, Barbosa, Estela; University of Bristol; UKPRP VISION Consortium Heaslip, Vanessa; University of Salford, Directorate of Nursing and Midwifery; University of Stavanger Sangar, Bob; University Hospitals Dorset NHS Foundation Trust Tbaily, Lee; University Hospitals Dorset NHS Foundation Trust Martin, Rachel; University Hospitals Dorset NHS Foundation Trust Docherty, Sharon ; Bournemouth University, Department of Medical Science and Public Health Allen, Helen; Bournemouth University, Bournemouth University Clinical Research Unit Hayward, Christopher; Plymouth University, Peninsula Clinical Trials Unit Marques, Elsa; University of Bristol, Bristol Medical School; University of Bristol, NIHR Biomedical Research Centre

**Primary Subject Heading**:	Health economics
Secondary Subject Heading:	Surgery
Keywords:	Foot & ankle < ORTHOPAEDIC & TRAUMA SURGERY, Clinical Trial, HEALTH ECONOMICS

SCHOLARONE™
Manuscripts

I, the Submitting Author has the right to grant and does grant on behalf of all authors of the Work (as defined in the below author licence), an exclusive licence and/or a non-exclusive licence for contributions from authors who are: i) UK Crown employees; ii) where BMJ has agreed a CC-BY licence shall apply, and/or iii) in accordance with the terms applicable for US Federal Government officers or employees acting as part of their official duties; on a worldwide, perpetual, irrevocable, royalty-free basis to BMJ Publishing Group Ltd ("BMJ") its licensees and where the relevant Journal is co-owned by BMJ to the co-owners of the Journal, to publish the Work in this journal and any other BMJ products and to exploit all rights, as set out in our licence.

The Submitting Author accepts and understands that any supply made under these terms is made by BMJ to the Submitting Author unless you are acting as an employee on behalf of your employer or a postgraduate student of an affiliated institution which is paying any applicable article publishing charge ("APC") for Open Access articles. Where the Submitting Author wishes to make the Work available on an Open Access basis (and intends to pay the relevant APC), the terms of reuse of such Open Access shall be governed by a Creative Commons licence – details of these licences and which Creative Commons licence will apply to this Work are set out in our licence referred to above.

Other than as permitted in any relevant BMJ Author's Self Archiving Policies, I confirm this Work has not been accepted for publication elsewhere, is not being considered for publication elsewhere and does not duplicate material already published. I confirm all authors consent to publication of this Work and authorise the granting of this licence.

For peer review only

**Title**	**Use of removable support boot vs cast for early mobilisation after ankle fracture surgery: Cost-effectiveness analysis and qualitative findings of the Ankle Recovery Trial. (ART)**

Petra Baji* (Corresponding author)	Musculoskeletal Research Unit, Bristol Medical School, Translational Health Sciences, University of Bristol, BS10 5NB Bristol, United Kingdom<br>Corvinus University of Budapest, Budapest, Hungary<br>Email: petra.baji@bristol.ac.uk
Estela C. Barbosa*	Bristol Medical School, Population Health Sciences, University of Bristol, BS10 5NB Bristol, United Kingdom<br>UKPRP VISION Consortium, London, UK<br>Email: e.capelasbarbosa@bristol.ac.uk
Vanessa Heaslip	Directorate of Nursing and Midwifery, University of Salford, United Kingdom<br>Department of Social Work, University of Stavanger, Norway
Bob Sangar	University Hospitals Dorset NHS Foundation Trust, United Kingdom
Lee Tbaily	University Hospitals Dorset NHS Foundation Trust, United Kingdom
Rachel Martin	University Hospitals Dorset NHS Foundation Trust, United Kingdom
Sharon Docherty	Department of Medical Science and Public Health, Bournemouth University, United Kingdom
Helen Allen	Bournemouth University Clinical Research Unit, Bournemouth University, United Kingdom

For peer review only

Christopher Hayward Peninsula Clinical Trials Unit, University of Plymouth, United Kingdom

Elsa Marques Musculoskeletal Research Unit, Bristol Medical School, Translational Health Sciences
NIHR Biomedical Research Centre, University of Bristol, Bristol
United Kingdom
e.marques@bristol.ac.uk

* shared first authorship

Key words Ankle fracture surgery, early mobilisation, plaster, support boot, RCT, cost-effectiveness.

Word count 3793

(excluding title page, abstract, tables and declarations and references)

For peer review only

Abstract (297/300)

Objectives: To estimate the cost-effectiveness of using a removable boot versus a cast following ankle fracture from the National Health Service and Personal Social Services (NHS+PSS) payer, and societal perspectives; and explore the impact of both treatments on participants' activities of daily living.

Design: Cost-effectiveness analyses and qualitative interviews performed alongside a pragmatic multi-centre randomised controlled trial.

Setting: Eight UK NHS secondary care trusts.

Participants: 243 participants (60.5% female, on average 48.2 years of age [standard deviation 16.4]) with ankle fracture. Qualitative interviews with sixteen participants.

Interventions: removable air boot vs plaster cast 2-weeks after surgery weight bearing as able with group specific exercises.

Primary and secondary outcome measures: quality-adjusted life years (QALYs) estimated from the EQ-5D-5L questionnaire, costs, and incremental net monetary benefit statistics (INMB) measured 12-weeks after surgery, for a society willing-to-pay £20,000 per QALY.

Results: Care in the boot group cost, on average, £88 (95% CI £22 to £155) per patient more than in the plaster group from the NHS+PSS perspective. When including all societal costs, the boot saved, on average, £676 per patient (95% CI -£337 to £1,689). Although there was no evidence of a QALY difference between the groups (-0.0020 [95% CI -0.0067 to 0.0026]), the qualitative findings suggest participants felt the boot enhanced their quality of life. Patients in the boot felt more independent and empowered to take on family responsibilities and social activities.

Conclusions: While the removable boot is slightly more expensive than plaster cast for the NHS+PSS payer at 12-weeks after surgery, it reduces productivity losses and the need for informal care while empowering patients. Given that differences in QALYs and costs to the NHS are small, the decision to use a boot or plaster following ankle surgery could be left to patients' and clinicians' preferences.

Trial Registration: ISRCTN15497399

Key words: Ankle fracture surgery, early mobilisation, plaster, support boot, RCT, cost-effectiveness.

For peer review only

Article Summary

Strengths and limitations of this study

- *We performed a full economic evaluation alongside a definitive multi-centre randomized controlled trial in the UK comparing a removable boot with plaster cast for treatment of ankle fractures two-weeks post-surgery weight bearing as able.*
- *Our study collected resource use data from 243 trial participants and a review of medical notes at the trial centres to estimate costs from both the National Health Service and Personal Social Services (NHS+PSS) payer, and the societal perspectives.*
- *The study was strengthened by a nested qualitative study to explore the impact of both treatments on participants' activities of daily living.*
- *The RCT was not powered to detect a difference in quality-of-life or costs and there is uncertainty in the economic result, with the confidence interval around the incremental net monetary benefit statistics crossing the null.*

For peer review only

Background

Every year there are about 20,000 people in England admitted for ankle fractures. (1) affecting people of all ages but majority of working age. (2) This has been associated with long-term disability and pain, resulting in time-off work and other economic consequences. (3, 4) According to a recent systematic review, the direct costs of ankle fractures ranges from $1,908 to $19,555 worldwide. (5) In England, the cost of inpatient hospital care for ankle fractures was estimated to exceed £63.1 million in 2016/17. (1) Costs from the social perspective are even higher due to indirect costs associated with fractures, such as productivity loss and informal care costs, that contribute to almost half of total costs. (6) A previous meta-analysis suggested that the early return to function should be the main goal following surgical management of fractures, (7) which could also reduce economic impact.

About half of patients with ankle fracture require surgery for ankle stabilisation. These patients are typically immobilised with a plaster cast immediately following surgery. After a couple of weeks, ankle management options are more varied. Another plaster cast can be applied for several more weeks, which allows weight bearing and maximizes support, but has been associated with joint stiffness, muscle atrophy, and deep vein thrombosis. (8-10) One alternative is to use an air boot that can be removed to enable ankle mobilisation exercises, and for comfort and personal care, but could increase the risk of complications. (10, 11) Systematic reviews conclude that well-designed, prospective studies are still needed to determine which immobilization technique offers the most benefit for treating ankle fractures. (7, 12) . Furthermore, evidence on cost-effectiveness of boots compared to casts is missing from the literature. (12)

The Ankle Recovery Trial (ART) compared early mobilisation in a removable boot with cast immobilisation following ankle fracture fixation where early weight bearing was encouraged in both groups. Participants were followed from two weeks until 12-weeks post-surgery and the trial included a full economic evaluation and nested qualitative study. Clinically there were no observed differences in ankle function between treatment groups at any time point. Overall early weight bearing in both the boot or cast was safe with low wound complication rates (7%) that, although more frequent in the boot group, were minor (13). The aim of this paper is to report the results of the cost-effectiveness and related qualitative analyses to further the evidence base and inform future national guidance on post-surgical ankle management.

For peer review only - http://bmjopen.bmj.com/site/about/guidelines.xhtml

Methods

Trial design and ethics approval

A health economic evaluation and qualitative component were included in the ART pragmatic multi-centre RCT to provide a more inclusive picture. The trial was conducted across eight UK NHS secondary care trusts between July 2015 and November 2018 and compared casts with removable support boots, combined with weight bearing as able, as methods of ankle fracture management from two weeks post-surgery. Most participants at the 6-week follow-up timepoint were expected to remove their plaster cast or boot and start to mobilise freely. Questionnaires were completed at randomization (2 weeks after surgery), then at 6-, 7-, and 12-weeks post-surgery. The primary clinical endpoint was the Olerud and Molander ankle score (OMAS) (14) measured at 7-weeks post-surgery, when participants should have adapted to mobilising without plaster cast or boot. The ART trial was approved by the South Central – Hampshire A Research Ethics Committee (reference 14/SC/1409) and was prospectively registered (ISRCTN: 15497399). Results on clinical effectiveness (such as primary outcome measure and secondary outcomes, such as OMAS at week 12, EQ-5D VAS scores at week 7 and 12, secondary mechanistic measures at 6 weeks, weight-bearing status, use of walking aids, return to driving and work, impact on daily activities at weeks 6 and 12, as well as complications and serious adverse events at 6 and 12 weeks) can be found in the final report to the Funder (13) and will be reported in a separate paper.

Patient and Public Involvement

Twelve patient advisors, most of whom had experienced an ankle fracture, advised on several aspects of the trial, including determining the minimum significant difference in the primary outcome, decision to include a qualitative component, refining data collection tools, and on patient facing materials and shaping the interview topic guide. Two advisors also attended the trial steering committee.

Participants

For peer review only - http://bmjopen.bmj.com/site/about/guidelines.xhtml

All adults (>16 years old) undergoing open reduction and internal fixation (ORIF) surgery for an unstable ankle fracture were eligible. Exclusion criteria were: open ankle fracture; concern about quality of fixation/wound integrity; requiring further stabilisation in/around the ankle (e.g. syndesmosis); active leg ulceration; poor skin condition at operating site; serious concomitant disease, diabetic/other sensory neuropathy; non-ambulatory prior to injury; inability to understand/complete outcome questionnaires; enrolment in other interventional research which may confound data collection; concomitant injuries which may affect rehabilitation.

Intervention and usual care

Participants were randomly allocated to receive either a removable air boot (intervention) or plaster cast (control) at the two-weeks post-surgery appointment. All participants were provided a group-specific exercise leaflet demonstrated by a research physiotherapist. Participants were encouraged to perform exercises as often as pain allowed, advised on gait re-education with crutches and progress to weight bearing as able. Removable support boots could be any make or model with at least two air cells, rigid (e.g. plastic) anterior and posterior outer sections, and were suitable for weight-bearing. Casts were standard below-knee rigid shells with soft lining which supported the ankle in plantigrade and were provided with a plaster shoe to allow weight-bearing. At the six-week appointment participants had their cast or boot removed (as appropriate), and were followed up for an additional 6 weeks, by which time they were expected to return to normal activities.

Overview of economic evaluation

We conducted a cost–utility analysis and a cost-effectiveness analysis, comparing the boot with the cast group at 12-weeks after ankle stabilisation surgery. The primary analysis took a National Health Service plus Personal Social Services (NHS+PSS) perspective. The secondary analysis took a societal perspective on costs, which included private expenses, informal care, and productivity losses. All analyses followed the pre-specified health economics analysis plan (15) and were performed in STATA version 17. The CHEERS 2022 checklist was followed for the reporting the results of the economic evaluations.

Resource use data collection for the economic evaluation

For peer review only

At 2-weeks post-surgery, the physiotherapist and/or plaster technicians recorded staff grade, time, and resources required to fit the boot or the plaster, including brand of boot. Research nurses/physiotherapists reviewed medical notes at trial centres to collect data on further hospital visits within the study 10-week period. Resources used within the first 2 weeks of surgery were not collected, as randomisation took place 2 weeks after surgery. Other resource use was collected from participants in postal questionnaires at 6- and 12- weeks follow-up points. Questionnaires asked about physiotherapy received in hospital or in the community; general practice (GP) contacts with doctors and nurses; additional outpatient appointments or A&E attendances and admissions to hospitals outside the recruiting centre; NHS equipment (e.g., walking aids); prescribed medications; contacts with social worker, home care help services, and food-at-home services; privately paid physiotherapy, medical equipment, and home care or food services; productivity losses measured in terms of time-off work (absenteeism) and unproductive time at work (presenteeism); and time spent on informal care by a friend or relative.

Outcome data collection for the economic evaluation

Outcome data were collected at baseline, 6-, 7-, and 12-weeks post-surgery. We used responses to the EQ-5D-5L questionnaire to compute a utility score to estimate quality-adjusted life years (QALYs) (16). The EQ-5D-5L is a patient-reported outcome instrument, standardised and validated to measure generic health-related quality of life (16). It includes five dimensions: mobility, self-care, usual activities, pain/discomfort, and anxiety/depression. It is widely used in economic analysis in clinical trials as it allows for the direct comparison of health benefits across different clinical interventions.

OMAS was the primary clinical outcome of the trial. The OMAS is patient-reported outcome measure with scores varying between 1 and 100. A clinically meaningful difference in OMAS was defined as 10-points or more(17).

Valuing resource use to derive costs

We used a micro-costing approach to estimate the cost of delivering treatment in both arms. We used local procurement prices for the removable boots and plaster materials, and valued staff grades and time spent delivering both treatments using 2020/21 prices from the Unit Costs of Health and Social Care 2021 (PSSRU)(18). Secondary care resources were valued

For peer review only - http://bmjopen.bmj.com/site/about/guidelines.xhtml

using the UK NHS National Collection of Costs 2019/20 (19) (2019/20 prices were inflated to 2020/21 prices using the NHS Cost Inflation Index (NHSCII) for Pay & Prices). Medication costs were derived from the British National Formulary for medications (2022 prices) (20). Productivity losses were valued using a human capital approach and the ONS averaged gross earning per hour (2021) for the relevant age group of the trial participants (age 40-49) (21), and the minimum wage rates between April 2021 to March 2022 for informal care (22). Unit costs applied and their sources are presented in the Appendix Table S1. Costs were calculated by multiplying the units of resource used by its unit cost.

Valuing EQ-5D-5L to derive QALYs

EQ-5D-5L utility scores at baseline, 6,7 and 12-weeks post-operative were mapped from the 3L version using Hernandez Alava and Pudney`s algorithm (23) Utility scores are bound at a maximum of 1 (corresponding to perfect health), where 0 corresponds to death. Negative values were permitted for health states worse than death. We calculated accumulated QALY gains per patient using the area-under-the-curve approach, assuming a linear change between utility scores at each time point(24). For comparison, we also produced utility scores using Van Hout's algorithm, which was the recommended mapping algorithm by NICE before 2022 (25).

Cost-Utility Analysis

Analyses were intention-to-treat, where participants' data were analysed based on their randomly allocated treatment group.

We imputed missing costs, utility scores, and Olerud and Molander ankle scores (OMAS) at each time point using multiple imputation models (26) with chained equations, with 50 sets and predictive mean matching, assuming that data were not missing completely at random (details of imputation model in Appendix Imputation of missing data). Costs and outcomes were then adjusted for hospital site (trial stratification variable) and pre-specified variables (age, sex, and fracture complexity) (15). QALYs and OMAS were further adjusted for baseline scores (24). We plotted costs and outcomes distributions to explore differences between the groups and cost drivers.

For peer review only

We jointly estimated the difference between groups in terms of costs and outcomes using Seemingly Unrelated Regressions (SUR) and computed the correlation of residuals.

We estimated cost-effectiveness in relation to QALYs from the NHS+PSS and societal perspectives using the Incremental Net Monetary Benefit (INMB) with a willingness-to-pay (WTP) threshold of £20,000 per QALY (27). We plotted the results of 1,000 bootstrapped iterations of the incremental costs and incremental effects in cost-effectiveness planes (CEP) and the corresponding cost-effectiveness acceptability curves (CEAC) to illustrate the uncertainty surrounding the decision to adopt the intervention.

Uncertainty and sensitivity analyses

We conducted a complete case analysis to illustrate the impact of missing data and the imputation model in results. To account for model uncertainty we adjusted costs and QALYs also including relationship status, alone living status, employment status, education, and body mass index. We carried out one-way sensitivity analysis for scenarios where we decreased the price of boots by 25%, 50% and 75% to explore the impact of lower prices of boots on the results.

Overview of the qualitative study

Participants were invited to participate in a qualitative interview towards the end of their study period, at approximately 12 weeks post-surgery. Two qualitative researchers chose individuals from the pool of positive responders, ensuring a balance of treatment options, sex, age, hospital site, and baseline OMAS scores. Participants were invited to a 45-60 minute telephone semi-structured interview; a date and time to suit the participants was set for those who agreed to participate. Data were audio recorded and transcribed verbatim. During the interviews, participants were asked to discuss their experiences of living with the treatment (boot or cast) as well as exploring the impact this had on their activities of daily living (personal hygiene, sleep, mobility, work, diet), psychological implications (mood and independence) as well as wider impact on their family and social life (changes to routines, caring responsibilities, finances).

Analysis of qualitative data

For peer review only - http://bmjopen.bmj.com/site/about/guidelines.xhtml

Qualitative interviews were analysed using the six-step process of thematic analysis by Braun and Clarke (28). We read transcripts to gain a sense of the whole before step-by-step analysis of each transcript to identify codes, categories, and initial themes. Once all interviews were analysed individually, a cross case analysis was undertaken, identifying themes across the whole data set. These themes and the process of analysis were shared with a second qualitative researcher to ensure credibility of the analytical process.

Results

Sample

243 participants were randomised between August 2015 and September 2018 (n=123 boot group and n=120 plaster group). Table 1 describes participant demographics and socio-economic characteristics by arm. No major differences were observed between trial arms for all variables.

Sixteen trial participants recruited across the 8 study sites consented to be interviewed; 6 were males and 10 were females, between the ages of 24 and 77 years (mean age was 49.2, median age was 50.5); 7 received a plaster cast and 9 received a boot. Most ankle fractures were simple (n=14), whilst two were comminuted (bone is broken into more than two pieces) and there was a range of baseline OMAS scores between 5 and 80.

Costs and outcomes

Table 2 reports costs and outcomes by trial arm including imputed data. Units of resource use per arm is presented in the Appendix, Table S2 and all available cost data (complete cost categories only) are presented in Appendix Table S3. The ankle management treatment cost difference reflects the difference in price between the boot (£50) and plaster (£25), and the difference in staff grade and time fitting each. On average, it took the technicians 25.3 minutes to fit the cast (SD 17.1) and 23.7 minutes to fit the boot (SD 24.9).

NHS+PSS cost differences are small and in favour of the plaster group. From a societal perspective, the differences are larger, particularly in relation to productivity loss and informal care, and favour the boot group (Figures S1 and S2 of the Appendix).

For peer review only - http://bmjopen.bmj.com/site/about/guidelines.xhtml

Cost-effectiveness base case and sensitivity analyses

Table 3 presents the cost-effectiveness analysis results from an NHS+PSS perspective and a societal perspective for the base case and sensitivity analyses. From an NHS+PSS perspective, treatment in the boot group costs, on average, £88 (95% CI £22, £155) per patient more than in the plaster group. From a societal perspective, the boot saved, on average, £676 per patient compared with plaster [95% CI -1,689, £337]). The cost savings for the boot are driven by reduced informal care and time-off productive work. There was no evidence of a QALY difference between the arms (mean QALY gain -0.0020 (95% CI -0.0067, 0.0026).

From an NHS+PSS perspective the boot is dominated, with a negative INMB statistic (mean -£129 [95% CI -£231, -£28)]). When including additional privately incurred costs, productivity losses and the burden of informal care in a societal perspective, the boot is cost-effective when willing-to-pay £20,000 per QALY, with a mean INMB £634, 95% CI [-£266, £1,535]). The societal perspective results, although deriving larger net benefits, are also more uncertain, with wider confidence intervals that cross the null.

The cost-effectiveness plane for the NHS+PSS perspective shows a high concentration of results in the upper left quadrant (Figure S3 a) of the Appendix) reflecting a dominated treatment option. This corresponds to a low probability of the boot being cost-effective even at high willingness-to-pay thresholds in the cost-effectiveness acceptability curve (Figure S4 a) of the Appendix). From a societal perspective (Figure S3 b) of the Appendix) shows a high concentration of results in the lower-left quadrant, but a higher likelihood of being cost-effective (Figure S4 b) of the Appendix).

These findings are robust in sensitivity analyses to the model specification. Our findings are sensitive to the imputation model. In a complete case analysis, the societal perspective results are closer to those of the NHS+PSS results, with the boot group reporting, on average, higher costs than the cast group. When boot prices decreased by 25%, 50% and 75% in sensitivity analyses, the NHS+PSS cost differences between arms decreased to £76 (95% CI £9, £142), £63 (95% CI -£3, £130) and £51 (95% CI -£16, £117), respectively, indicating no evidence of a differences in costs, QALYs, or INMB statistics (see Appendix Table S4).

Qualitative findings

For peer review only - http://bmjopen.bmj.com/site/about/guidelines.xhtml

The economic results of lower productivity losses and informal care costs in the boot group were reflected in the qualitative interviews. Having a fracture irrespective of the treatment offered (boot or cast) increased reliance on others and this was typically managed by family members and friends which was frustrating for participants, many of whom expressed feeling helpless (Table 4 – Feelings).

Those with the boot expressed an ability to become either more self-caring or take back family responsibilities earlier, such as walking to the kitchen to prepare dinner and making their own drinks. These activities were harder for those in cast due to immobility (Table 4 – Daily activities).

It was also apparent that having a boot enabled participants to continue their wider social connections; they travelled further and continued with wider social engagements (outside the home) than those with a cast. In contrast, those with a cast talked about having to miss family engagements and wider social activities where people with the boot tended to continue to attend these, even if it required hiring additional support, such as a wheelchair (Table 4 – Social life).

Patients in the cast group appeared to have increased personal costs purchasing additional clothing and equipment to accommodate the use of the cast (Table 4 – Private expenses).

Patients in the cast group expressed difficulty with stair climbing, while those with boots who described being able to weight bear did not express similar difficulties. Another advantage identified by participants in boots was the ability to remove it to undertake the prescribed exercises, which they felt aided a quicker recovery (Table 4 - Mobility). Of the five participants who expressed a preference, two in cast would have preferred boots and the other three were glad they had received boots.

In the qualitative interviews, participants reported that they felt the boot enhanced their quality of life (Table 4 – Quality of life).

Discussion

Statement of principal findings

For peer review only - http://bmjopen.bmj.com/site/about/guidelines.xhtml

We found that the boot group had, on average, higher NHS+PSS costs but differences between groups were small. Differences in QALY gains between groups were negligible. When including costs with informal care burden and productivity losses from time-off or unproductive work, the boot saves society on average £676, and could be a cost-effective intervention when willing-to-pay £20,000 per QALY. Qualitative interviews with sixteen participants complemented the quantitative analysis to better understand patients' perspectives. Lower productivity losses and informal care need in the boot group were reflected in the interviews, and some participants in the boot group highlighted that they were more satisfied with their treatment option, felt more independent, and were able to return to their usual activities sooner.

Strengths and limitations

Our study reports a full economic evaluation from an NHS+PSS perspective and a nested qualitative study conducted alongside a large pragmatic multicentre RCT in the management of ankle factures after surgery. The nested qualitative study shed further light onto the interpretation of economic findings. The ART trial is the largest RCT to date comparing removable support boot with a plaster cast for early mobilisation after ankle fracture surgery and ours is the first cost-effectiveness evaluation comparing the two ankle management treatment options.

Despite data collection taking place several years ago, we believe our results are relevant and transferable to current NHS practice. The ART trial was powered to detect a difference in the primary outcome (OMAS score at 7 weeks), and not difference in costs, QALYs, or INMB statistics, consequently there is more uncertainty in the results, especially from the societal perspective. Missing data for the economic evaluation was relatively high and not completely at random. Our base-case results include imputed data, due to the risk of biases in a complete case analyses, which changed the results for the societal perspective. The larger cost driver in favour of the boot is informal care burden. Measuring the burden for carers in economic evaluation is often neglected and methods to value carer burden are not contemplated in the NICE reference case.(27)

The EQ-5D-5L questionnaire appears to be too insensitive to pick up important aspects of quality of life that the boot can provide to patients. It does not directly measure the broader aspects of wellbeing and ability that were captured in the qualitative interviews. The

For peer review only - http://bmjopen.bmj.com/site/about/guidelines.xhtml

perception of increased wellbeing in the qualitative interviews, however, may be a result of expectations towards the newer technology, which is difficult to objectively measure.

Results in context

A recent systematic review of the clinical effectiveness and cost-effectiveness of orthotic walking boots for patients with ankle fractures or ligament injuries, found no previous cost-effectiveness studies comparing boots with casts (12). According to our knowledge, this is the first cost-effectiveness evaluation alongside a trial comparing boots with cast.

Nevertheless, a few other studies have also compared resource use, return to work or to normal activities between functional treatment and cast groups. For example, Egol et al (2000) reported earlier return to work in the brace group for patients who underwent internal fixation,(29) however these patients were all instructed to avoid weight-bearing on the affected side for six weeks. Honignmann (2007) found that for patients who had malleolar fractures followed by open reduction and internal fixation, patients in the orthesis group with prescribed full weight bearing returned to work sooner, however the difference was not significant.(30) Simansky et al (2006) and Lehtonen et al (2003) no significant differences in return to work comparing functional treatment versus cast patients who underwent open reduction and internal fixation. (11, 31)

In our study, no differences were observed in how soon participants returned to driving or full pre-injury work duties. The boot group indicated at the 6-week post-operative time point that their injury was having a greater impact on daily activities; however, this difference was no longer present 4 weeks later.

Similarly to our results, Kearney et al. (2021) and Haque et al. (2023) found no significant difference between brace and cast groups in terms of EQ 5D-5L scores at any time point (32, 33).

Conclusion

For peer review only - http://bmjopen.bmj.com/site/about/guidelines.xhtml

While the boot is more expensive than cast for the NHS+PSS payer, it reduces productivity losses and the need for informal care and empowers patients. Given that differences in QALYs and costs to the NHS are small, the decision to use a boot or cast following ankle surgery could be left to patients' and clinicians' preferences.

List of Abbreviations

ART – The Ankle Recovery Trial

CEAC – Cost-effectiveness Acceptability Curve

CEA – Cost-Effectiveness Analysis

CUA – Cost Utility Analysis

INMB – Incremental Net Monetary Benefit

NHS – National Health System

NICE - National Institute for Health Care and Excellence

OMAS – Olerud and Molander Scoring System

PSS – Personal Social Services

QALY – Quality-Adjusted Life Year

QoL – Quality-of-Life

RCT – Randomised Controlled Trial

SUR - Seemingly Unrelated Regressions

UK – United Kingdom

WTP – Willingness to Pay

For peer review only

Declarations

Acknowledgements

The views expressed herein are those of the authors and not necessarily those of the NHS, the NIHR, or the Department of Health and Social Care.

We thank Pete Thomas for his immerse contribution in designing the study, securing study funding and in writing the statistical analysis plan that had influenced the health economics analysis plan.

The protocol for the study

The protocol for the study is available via:

ART: Ankle Recovery Trial Does early mobilisation after Ankle fracture surgery enhance Recovery? A randomised controlled Trial comparing the use of plaster versus removable support boot. Study Protocol. Version 1.6 – 05/03/2018 Available at: https://www.isrctn.com/editorial/retrieveFile/7b25ff02-5f9f-4c56-b51d-3a8cf6a33197/30174. Accessed: 28/09/2023

SHEAP is available via:

STATISTICAL AND HEALTH ECONOMICS ANALYSIS PLAN ART: Ankle Recovery Trial Does early mobilisation after Ankle fracture surgery enhance Recovery? A randomised controlled Trial comparing the use of plaster versus removable support boot. Available at: https://www.isrctn.com/editorial/retrieveFile/0af5db91-f4d3-407a-9f19-500a904edfbc/30174. Accessed: 28/09/2023

Funding statement

This report is independent research funded by the National Institute for Health and Care Research (NIHR) under its Research for Patient Benefit Programme 'Does early mobilisation after Ankle fracture surgery enhance Recovery? A pragmatic multi-centre randomised controlled Trial with qualitative component and health economic analysis comparing the use of plaster versus support boot (ART)', PB-PG-0213-30021. The views expressed in this publication are those of the author(s) and not necessarily those of the NHS, the National Institute for Health and Care Research or the Department of Health and Social Care.

A competing interests statement

The authors declare that they have no competing interests.

For peer review only

Author contributions

AS, RM, EMRM, CH, HA, conceived the idea for the study and secured funding. EMRM designed the economic evaluation and supervised PB and ECB in performing the economic evaluation. PB, ECB, EMRM had access to data and conducted the economic analysis. VH designed and VH and HA conducted the qualitative study. LT was trial manager with oversight of data collection and management. SD managed the data sources and performed the analysis of clinical outcomes. ECB, PB, EMRM, and VH drafted the manuscript. All authors critically revised the manuscript and approved the final version for submission. PB and ECB contributed equally to this paper as first authors.

Patient consent form

Informed Consent forms were filled in by each patients participated in the ART trial (ISRCTN15497399). No individual person's data are included in the manuscript.

Data sharing statement

The datasets used and/or analysed during the current study are available from the corresponding author on reasonable request.

For peer review only

For peer review only - http://bmjopen.bmj.com/site/about/guidelines.xhtml

References

1. Scott LJ, Jones T, Whitehouse MR, Robinson PW, Hollingworth W. Exploring trends in admissions and treatment for ankle fractures: a longitudinal cohort study of routinely collected hospital data in England. BMC Health Services Research. 2020;20:1-10.
2. Gill JR, Lostis E, Bleibleh SS, Hennessy MS, Thondickal F, Gadd RJ, et al. Weight-bearing in ankle fractures: An audit of UK practice. The Foot. 2019;39:28-36.
3. McPhail SM, Dunstan J, Canning J, Haines TP. Life impact of ankle fractures: Qualitative analysis of patient and clinician experiences. BMC musculoskeletal disorders. 2012;13(1):1-13.
4. Nilsson GM, Jonsson K, Ekdahl CS, Eneroth M. Unsatisfactory outcome following surgical intervention of ankle fractures. Foot and Ankle Surgery. 2005;11(1):11-6.
5. Bielska IA, Wang X, Lee R, Johnson AP. The health economics of ankle and foot sprains and fractures: A systematic review of English-language published papers. Part 2: The direct and indirect costs of injury. The Foot. 2019;39:115-21.
6. Noback PC, Freibott CE, Dougherty T, Swart EF, Rosenwasser MP, Vosseller JT. Estimates of direct and indirect costs of ankle fractures: a prospective analysis. JBJS. 2020;102(24):2166-73.
7. Lin CWC, Donkers NA, Refshauge KM, Beckenkamp PR, Khera K, Moseley AM. Rehabilitation for ankle fractures in adults. Cochrane database of systematic reviews. 2012(11).
8. Shaffer MA, Okereke E, Esterhai Jr JL, Elliott MA, Walter GA, Yim SH, et al. Effects of immobilization on plantar-flexion torque, fatigue resistance, and functional ability following an ankle fracture. Physical Therapy. 2000;80(8):769-80.
9. Stevens JE, Walter GA, Okereke E, Scarborough MT, Esterhai JL, George SZ, et al. Muscle adaptations with immobilization and rehabilitation after ankle fracture. Medicine and science in sports and exercise. 2004;36(10):1695-701.
10. Vioreanu M, Dudeney S, Hurson B, Kelly E, O'Rourke K, Quinlan W. Early mobilization in a removable cast compared with immobilization in a cast after operative treatment of ankle fractures: a prospective randomized study. Foot & ankle international. 2007;28(1):13-9.
11. Lehtonen H, Järvinen TL, Honkonen S, Nyman M, Vihtonen K, Järvinen M. Use of a cast compared with a functional ankle brace after operative treatment of an ankle fracture: a prospective, randomized study. JBJS. 2003;85(2):205-11.
12. Banerjee S, Ryce A. Orthotic Walking Boots for Patients with Fractures or Ligament Injuries: A Review of Clinical Effectiveness and Cost-Effectiveness. 2019.
13. Final report submitted to National Institute for Health Research (NIHR), Research for Patient Benefit. Project Title: Does early mobilisation after Ankle fracture surgery enhance Recovery? A pragmatic multi-centre randomised controlled Trial with qualitative component and health economic analysis comparing the use of plaster versus support boot (ART)

Reference Number: PB-PG-0213-30021. Availale from authors upon request.
14. Olerud C, Molander H. A scoring scale for symptom evaluation after ankle fracture. Archives of orthopaedic and traumatic surgery. 1984;103:190-4.
15. Thomas P, Marques E, Docherty S, Sangar S. Statistical and Health Economics Analysis Plan: ART Ankle Recovery Trial version 1.1. University of Bristol. 28th Nov 2018. Available at: https://research-information.bris.ac.uk/en/publications/statistical-and-health-economics-analysis-plan-art-ankle-recovery Accessed: 26/01/2023 2018.
16. Herdman M, Gudex C, Lloyd A, Janssen M, Kind P, Parkin D, et al. Development and preliminary testing of the new five-level version of EQ-5D (EQ-5D-5L). Quality of life research. 2011;20(10):1727-36.
17. Olerud C, Molander H. A scoring scale for symptom evaluation after ankle fracture. Archives of orthopaedic and traumatic surgery. 1984;103(3):190-4.
18. Jones KC, Burns A. Unit Costs of Health and Social Care 2021. Unit Costs of Health and Social Care . . Kent, UK: Personal Social Services Research Unit 2021.

For peer review only - http://bmjopen.bmj.com/site/about/guidelines.xhtml

19. England N. NHS England: 2019/20 National Cost Collection Data Publication. National Schedule of NHS Costs 2019/20. First published: 22 June 2021. 2021.
20. British Medical Association and the Royal Pharmaceutical Society. BNF 83 British National Formulary March - September 2022.
21. Statistics OfN. Annual Survey of Hours and Earnings (ASHE) Earnings and hours worked, age group: ASHE Table 6, Table 6.5a Hourly pay - Gross (£) - For all employee jobsa: United Kingdom, 2021. Release date: 26 October 2021. 2021.
22. Gov.uk: Minimum wage rates for 2022. Available: https://www.gov.uk/government/publications/minimum-wage-rates-for-2022. Accessed: 23/06/2022 [
23. Hernández-Alava M, Pudney S. Econometric modelling of multiple self-reports of health states: the switch from EQ-5D-3L to EQ-5D-5L in evaluating drug therapies for rheumatoid arthritis. Journal of Health Economics. 2017;55:139-52.
24. Manca A, Hawkins N, Sculpher MJ. Estimating mean QALYs in trial-based cost-effectiveness analysis: the importance of controlling for baseline utility. Health economics. 2005;14(5):487-96.
25. Van Hout B, Janssen M, Feng Y-S, Kohlmann T, Busschbach J, Golicki D, et al. Interim scoring for the EQ-5D-5L: mapping the EQ-5D-5L to EQ-5D-3L value sets. Value in health. 2012;15(5):708-15.
26. Faria R, Gomes M, Epstein D, White IR. A guide to handling missing data in cost-effectiveness analysis conducted within randomised controlled trials. Pharmacoeconomics. 2014;32(12):1157-70.
27. National Institute for Health and Care Excellence. NICE health technology evaluations: the manual. Published: 31 January 2022
28. Braun V, Clarke V. Using thematic analysis in psychology. Qualitative research in psychology. 2006;3(2):77-101.
29. Egol K, Dolan R, Koval K. Functional outcome of surgery for fractures of the ankle: a prospective, randomised comparison of management in a cast or a functional brace. The Journal of Bone & Joint Surgery British Volume. 2000;82(2):246-9.
30. Honigmann P, Goldhahn S, Rosenkranz J, Audigé L, Geissmann D, Babst R. Aftertreatment of malleolar fractures following ORIF—functional compared to protected functional in a vacuum-stabilized orthesis: a randomized controlled trial. Archives of orthopaedic and trauma surgery. 2007;127:195-203.
31. Simanski CJ, Maegele MG, Lefering R, Lehnen DM, Kawel N, Riess P, et al. Functional treatment and early weightbearing after an ankle fracture: a prospective study. Journal of orthopaedic trauma. 2006;20(2):108-14.
32. Haque A, Parsons H, Parsons N, Costa M, Redmond A, Mason J, et al. Use of cast immobilization versus removable brace in adults with an ankle fracture: two-year follow-up of a multicentre randomized controlled trial. Bone and Joint Journal. 2023;105(4).
33. Kearney R, McKeown R, Parsons H, Haque A, Parsons N, Nwankwo H, et al. Use of cast immobilisation versus removable brace in adults with an ankle fracture: multicentre randomised controlled trial. bmj. 2021;374.

For peer review only - http://bmjopen.bmj.com/site/about/guidelines.xhtml

Tables

Table 1 - Participants baseline characteristics

		Boot (n = 123)		Cast (n = 120)	
		**Mean**	**SD**	**Mean**	**SD**
Age	n=243	48.75	15.71	47.7	17.1
BMI	n=239	28.29	5.79	27.42	5.54
		**Count**	**%**	**Count**	**%**
Gender	Male	46	37.4%	49	40.8%
	Female	77	62.6%	70	58.3%
	Missing	0	0.0%	1	0.8%
Fracture complexity	Simple	101	82.1%	99	82.5%
	Other	21	17.1%	19	15.8%
	Missing	1	0.8%	2	1.7%
Living alone prior to injury	Yes	96	78.0%	95	79.2%
	No	25	20.3%	18	15.0%
	Missing	2	1.6%	7	5.8%
Marital status	Single	34	27.6%	34	28.3%
	Married	56	45.5%	61	50.8%
	Civil partnership	8	6.5%	9	7.5%
	Divorced/ partnership dissolved	16	13.0%	3	2.5%
	Widowed/surviving civil partner	6	4.9%	6	5.0%
	Missing	3	2.4%	7	5.8%
Education	None	9	7.3%	15	12.5%
	GCSE and A/AS level	49	39.8%	47	39.2%
	First degree	21	17.1%	18	15.0%
	Higher degree	19	15.4%	14	11.7%
	Other	11	8.9%	13	10.8%
	Missing	14	11.4%	13	10.8%
Employment	Full time paid employment	62	50.4%	53	44.2%
	Part time paid employment	15	12.2%	15	12.5%
	Retired	24	19.5%	20	16.7%
	Volunteer	0	0.0%	1	0.8%
	Unemployed	4	3.3%	4	3.3%
	Looking after home	7	5.7%	7	5.8%
	Full time education	2	1.6%	6	5.0%
	Other	7	5.7%	7	5.8%
	Missing	2	1.7%	7	5.8%

For peer review only

Table 2 – Costs and outcomes by trial arm and by perspective on costs (imputed data)

	**Boot (N=123)**	**Cast (N=120)**	**Difference**
NHS+PSS	Mean (95% CI)	Mean (95% CI)	Mean (95% CI)
Ankle treatment (boot or cast)	£203 (£200, £206)	£179 (£177, £181)	£24 (£20, £28)
Physiotherapy in the community	£11 (£4, £18)	£4 (£1, £7)	£7 (-£1, £14)
Physiotherapy in hospital	£26 (£15, £37)	£24 (£12, £36)	£2 (-£14, £18)
GP practice services	£57 (£30, £83)	£37 (£9, £64)	£20 (-£18, £58)
Social Care services	£19 (£8, £30)	£6 (£0, £12)	£13 (£0, £25)
A&E and Outpatient services	£55 (£25, £85)	£31 (£18, £43)	£24 (-£9, £57)
Hospital Admissions	£8 (-£7, £23)	£8 (-£7, £24)	-£0 (-£22, £22)
NHS Equipment	£3 (£1, £5)	£3 (£1, £5)	£0 (-£2, £3)
Medications	£7 (£4, £9)	£5 (£1, £8)	£2 (-£2, £6)
Private costs and productivity losses			
Private physiotherapy	£16 (£5, £27)	£19 (£7, £31)	-£3 (-£19, £13)
Additional medical Equipment	£5 (£2, £9)	£1 (-£0, £2)	£4 (£0, £8)
Private community care	£0 (-£0, £1)	£1 (-£1, £4)	-£1 (-£3, £2)
Other private expenditures on services/activities	£105 (£75, £134)	£139 (£31, £247)	-£34 (-£145, £76)
Other major expenditure not reported elsewhere	£10 (-£1, £20)	£20 (£2, £38)	-£10 (-£31, £10)
Productivity Loss (time-off work and leisure)	£2,359 (£1,912, £2,807)	£2,995 (£2,334, £3,657)	-£636 (-£1,427, £155)
Informal Care	£888 (£567, £1,209)	£1,025 (£630, £1,420)	-£137 (-£642, £367)
Outcomes			
QALYs gained (Hernandez Alava and Pudney)	0.1195 (0.1144, 0.1247)	0.1248 (0.1208, 0.1287)	-0.0052 (-0.0117, 0.0013)
QALYs gained (Van Hout)	0.1117 (0.1062, 0.1172)	0.1189 (0.1146, 0.1231)	-0.0071 (-0.0141, -0.0002)
Olerud and Molander (primary outcome, Week7-Week2 baseline)	-55.0 (-59.1, -51.0)	-59.0 (-62.3, -55.7)	4.0 (-1.2, 9.2)
Olerud and Molander (Week12-Week2 baseline)	-39.7 (-43.7, -35.8)	-35.5 (-39.1, -31.8)	-4.3 (-9.7, 1.1)

Note: Costs are in 2020/21 prices.

For peer review only - http://bmjopen.bmj.com/site/about/guidelines.xhtml

Table 3 – Cost Utility Analysis results

Cost Utility Analysis	NHS+PSS Perspective Mean (95% CI)	Societal perspective Mean (95% CI)
**Base Case**		
Incremental Cost	£88 (£22 to £155)	-£676 (-£1,689 to £337)
Incremental QALY	-0.0020 (-0.0067 to 0.0026)	-0.0021 (-0.0067 to 0.0026)
Net Monetary Benefit	-£129 (-£231 to -£28)	£634 (-£266 to £1,535)
Probability boot is cost-effective at £20,000/QALY	1.3%	88.6%
**Sensitivity Analysis**		
Models type 2 – adjusting for additional covariates		
Incremental Cost	£74 (£8 to £140)	-£1,017 (-£1,967 to -£67)
Incremental QALY	-0.0021 (-0.0067 to 0.0025)	-0.0021 (-0.0067 to 0.0025)
Net Monetary Benefit	-£116 (-£217 to -£15)	£974 (£99 to £1,850)
Probability boot is cost-effective at £20,000/QALY	2.3%	97.5%
Complete Case Analysis		
Incremental Cost	£123 (£19 to £227)	£323 (-£934 to £1,580)
Incremental QALY	-0.0010 (-0.0076 to 0.0056)	-0.0027 (-0.0096 to 0.0041)
Net Monetary Benefit	-£143 (-£327 to £40)	-£378 (-£1,771 to £1,015)
Probability boot is cost-effective at £20,000/QALY	4.1%	28.3%

Models type 1 (base case) adjust for age, gender, fracture complexity and hospital site; Models type 2 also include relationship status (married/partnership vs other), alone living status, employment status (working part or full time vs other), education (higher degree vs other) and BMI;
INMB = incremental net monetary benefit measured at a willingness to pay for a QALY of £20,000. QALY = quality-adjusted life year.

For peer review only

Table 4 Quotes from the qualitative interviews

Topic	Quotes from the qualitative interviews
Feelings related to ankle fracture	*"I had depression before and I think that didn't help that I've got that tendency as well…there was some sense of isolation more to do with not being able to see people a lot of the time or not having the same routine. The other thing I found really challenging was having to ask for help*" (Female 44 cast)<br><br>*"It had a huge impact on my independence because it meant I was relying on other people to even leave the house"* (Female 52 cast)
Daily activities	*"There's only so much you want to be waited on, everyone says I dream of that but when it comes to it, it actually drives you insane, to be able to get back and cook meals for my children and to be able to go and make myself a cup of tea and then carry it to another room is fantastic* (Female 46 boot)<br><br>*"…for me to be reliant on somebody else, it was degrading…and I felt vulnerable like in a really vulnerable state definitely …I found* [with boot] *I was less dependent on other people because I could move around a bit more, I could have a bath or shower myself, I could get in and out of bed on my own"* (Female 27 boot)<br><br>*"It put a huge burden on my daughter who was having to come 4 times a day, but then [with boot] I could go into the kitchen for a large degree to, make a meal for myself you know"* (Female 77 boot)
Social life and events	*"Basically, I was stuck around the house, we had to cancel our holiday… to be honest I just sat round the house pretty much for six weeks"* (Male 69 cast)<br><br>*"there were family BBQs where basically my husband took the kids and I just relaxed at home, it was a bit too much"* (Female 44 cast)<br><br>*"I feel the boot is a very good protection around my leg and ankle particularly if I was out and I was using the wheelchair quite a lot as we have a busy social life… I think would have been more twitchy had I had a plaster"* (Female 69 boot)

For peer review only - http://bmjopen.bmj.com/site/about/guidelines.xhtml

	"We hired a wheelchair for me because we went to the [event] with the kids in [place] and that was a whole day come night-time thing" (Female 46 boot)
Private expenses	*"I obviously had to buy a waterproof cover for the leg…. I went out and brought a couple of pairs of cropped leggings…I was obviously very limited what I could wear because of the plaster, probably spent about £30 on the cover and leggings"* (Female 52 cast)<br><br>*"I could still wear leggings, I didn't have to cut up any trousers"* (Female 27 boot)<br><br>*"I bought one of those like big hot water bottle kind of thing that went up your leg for showers and things which costs about £14"* (Female 49 cast)<br><br>*"I had to cut up 4 pairs of my leggings up because my leggings wouldn't go over the plaster"* (Female 54 cast)
Mobility	*"…because the foot got really swollen* [from toes to my knee], *so the advantage of taking the boot off and putting peas on it, in your own mind you were doing something to ease the cause"* (Female 53 boot)<br><br>*"One of the main advantages was I was able to start my physio a lot earlier… I was able to practice flexing and getting my foot up and turning it. The flexibility in my foot its better, it's completely back to normal now where I think if it was in a cast… everything would have started to seize up a lot more and would have been harder to get the flexibility back"* (Female 46 boot).
Quality of life	*"I found the boot easier than the plaster. The plaster was very heavy and I found with the boot I could actually weight bear a bit more and get on with day to day running of life"* (Female 53 boot)<br><br>*"I wanted the boot because I knew the boot would be better for me personally… because I am so active I wanted to get around and do things"* (Male 24 boot)

For peer review only - http://bmjopen.bmj.com/site/about/guidelines.xhtml

For peer review only

For peer review only - http://bmjopen.bmj.com/site/about/guidelines.xhtml

Appendix

Use of removable support boot vs plaster for early mobilisation after ankle fracture surgery: Cost-effectiveness analysis and qualitative findings of the Ankle Recovery Trial. Baji et al.

Imputation of missing data

We imputed missing cost variables, utility scores, and Olerud and Molander ankle scores (OMAS) at each time point using multiple imputation methods (21). We applied chained equations, with 50 sets and predictive mean matching, assuming that data was not missing completely at random. Our imputation model considered treatment arm, hospital site, age, sex, body mass index, relationship status (married/partnership vs other), if the participant lived alone before the injury, education (higher degree vs no higher degree), employment status (working full-time or part-time vs not working) as well as fracture complexity (complex vs not complex), baseline utility, and baseline OMAS scores.

Stata code for imputation

```
mi set wide //register dataset to be imputed
mi register imputed costphysiogp_4 costphysiohospital_4 costphysioprivate_4 /*
 */ costgp_4 costsocialcare_4 costcommunityprivate_4 /*
 */ costhospitalAEoutpatient_4 costhospitaladmission_4 /*
 */ costequipmentprivate_4 costequipmentnhs_4 costotherprivate_4 /*
 */ costmedication_4 costphysiogp_10 costphysiohospital_10 costgp_10 /*
 */ costsocialcare_10 costhospitalAEoutpatient_10 costhospitaladmission_10  /*
 */ costequipmentnhs_10 costequipmentprivate_10 costotherprivate_10  /*
 */  cost_timeoff_4 cost_informalcare_4 cost_majorimpactprivate_4   /*
 */  costmedication_10 costphysioprivate_10  /*
 */   cost_timeoff_10 cost_informalcare_10 cost_majorimpactprivate_10 cost_fitting
cost_intervention //cost variables to be imputed
mi register imputed utility_4 utility_5 utility_10 //QALY variables to be imputed
mi register imputed OreludMolander_w5 OreludMolander_w10 // outcome variables to be
imputed
mi register imputed female complexity utility_baseline OreludMolander_baseline bmi
married_dummy alone work_dummy degree // stratification variables to be imputed
mi register regular treatmentgroup age  site   //regular variables that do not require imputation

mi impute chained (pmm , knn(10)) costphysiogp_4 costphysiohospital_4
costphysioprivate_4 /*
 */ costgp_4 costsocialcare_4 costcommunityprivate_4 /*
 */ costhospitalAEoutpatient_4 costhospitaladmission_4 /*
 */ costequipmentprivate_4 costequipmentnhs_4 costotherprivate_4 /*
 */ costmedication_4 costphysiogp_10 costphysiohospital_10 costgp_10 /*
 */ costsocialcare_10 costhospitalAEoutpatient_10 costhospitaladmission_10  /*
```

For peer review only

```
*/ costequipmentnhs_10 costequipmentprivate_10 costotherprivate_10  /*
*/  cost_timeoff_4 cost_informalcare_4 cost_majorimpactprivate_4   /*
*/  costmedication_10 costphysioprivate_10  /*
*/   cost_timeoff_10 cost_informalcare_10 cost_majorimpactprivate_10 /*
*/ cost_fitting cost_intervention utility_4 utility_5 utility_10 /*
*/ OreludMolander_w5 OreludMolander_w10  /*
*/  female complexity utility_baseline OreludMolander_baseline bmi married_dummy alone
work_dummy degree= treatmentgroup age  site , add(50) by(treatmentgroup) rseed(10) force
```

For peer review only

Table S1. Unit costs

Item	Cost (£)	Source
**Physiotherapy services**		Unit Costs of Health and Social Care 2020/21 (PSSRU)
Provided by GP surgery/practice – at the surgery/practic	40.5	Community-Based Scientific and Professional Staff, cost per hour, Band 5, physiotherapist, occupational therapist, speech and language therapist, podiatrist, clinical psychology assistant practitioner (higher level) counsellor (entry level), 1 hour
Provided by GP surgery/practice – at home	41.5	Community-Based Scientific and Professional Staff, cost per hour, Band 5, physiotherapist, occupational therapist, speech and language therapist, podiatrist, clinical psychology assistant practitioner (higher level) counsellor (entry level), 1 hour
Provided by a hospital – at home	39.5	Hospital based scientific and professional, cost per hour, Band 5, Physiotherapist, Occupational therapist, Speech and language therapist, Podiatrist, Clinical psychology assistant practitioner (higher level), Counsellor (entry level), 1 hour
Provided by a private clinic - at clinic	51.8	Hospital based scientific and professional, cost per hour, Band 6, Physiotherapist, Occupational therapist, Speech and language therapist, Podiatrist, Clinical psychology assistant practitioner (higher level), Counsellor (entry level), 1 hour
Consultations with an Occupational Therapist	88.4	Community-Based Scientific and Professional Staff, cost per hour Band 8.b., Physiotherapist consultant, Occupational therapist consultant, Clinical psychologist principal, Speech and language therapist principal, Podiatric consultant (surgery), Arts therapis t principal, 1 hour
**Community-based Services**		Unit Costs of Health and Social Care 2020/21 (PSSRU)
GP at the surgery/practice	33.0	Primary care practice professionals, Cost per surgery lasting 9.22 minutes (including direct care)
GP at home	132.0	Primary care practice professionals, GP Cost per hour (including direct care)
GP telephone contact	33.0	Primary care practice professionals, Cost per surgery lasting 9.22 minutes (including direct care)
Practice Nurse / Health Care Assistant at GP surgery	42.1	Primary care practice professionals, GP Practice Nurse (cost/hour)
Practice Nurse telephone contact	42.1	Primary care practice professionals, GP Practice Nurse (cost/hour)
Nurse at home	42.1	Primary care practice professionals, GP Practice Nurse (cost/hour)
Health Visitor	34.9	Community-based scientific and professional staff, cost per hour, Band 4

For peer review only

Home care/Home help	23.1	Social care staff, cost per hour, Home Care Worker
Food at home services (e.g. meals on wheels)	24.6	Social care staff, cost per hour, Support and outreach worker
Voluntary Agency Worker/Contact (e.g. from Age UK)	24.6	Social care staff, cost per hour, Support and outreach worker
**Hospital Services**		
Accident and Emergency	187.8	2019-20_National_schedule_of_NHS_costs_FY19_20_V2, A&E visits weighted average, inflated to 2020/21 prices using NHSCII Pay & Prices
Day case admission or procedure (but did not spend the night)	838.0	2019-20_National_schedule_of_NHS_costs_FY19_20_V2, Day cases weighted average, inflated to 2020/21 prices using NHSCII Pay & Prices, Assuming there is a procedure
Outpatient appointment	136.9	2019-20_National_schedule_of_NHS_costs_FY19_20_V2, OP appointments weighted average, inflated to 2020/21 prices using NHSCII Pay & Prices
**Time off work (productivity loss)**	20.4	Gross earning per hour, 2021, Earnings and hours worked, age group: ASHE Table 6 - Office for National Statistics (ons.gov.uk), Table 6.5a Hourly pay - Gross (£) - For all employee jobsa: United Kingdom, 2021, Age group 40-49
**Support from others (informal care)**	8.91	Minimum wage between April 2021 to March 2022, Minimum wage rates for 2022 - GOV.UK (www.gov.uk)
**Special Equipment (NHS costs)**		
Stick	3.5	Hospital procurement, 2022
One Crutch	3.58	
Two Crutches	7.16	
Frame	18.5	
Raised toilet seat	7.2	
Perching stool	17	
Bath support	11	
Shower seat	26.77	
Comode toilet	19.83	
Toilet rails	4.2	
Commode with 2 Zimmer Frames	56.83	
**Treatment costs**		
Boot	50	Hospital procurement
Plaster	25	Hospital procurement

For peer review only

Plaster technician (cost per hour)		Unit Costs of Health and Social Care 2020/21 (PSSRU)
Physiotherapist Band 4	35	Hospital based scientific and professional, cost per hour
Physiotherapist Band 5	39	Hospital based scientific and professional, cost per hour
Physiotherapist Band 6	52	Hospital based scientific and professional, cost per hour
Private expenses		Gym sessions and other sport sessions, Child care, Home cleaner, Food delivery (e.g. take away, eating out), Other, Equipments bought privately - Expenses provided by participant in the questionnaire, Inflated to 2020/21 prices using CPI
**Medication cost (Prescribed&out-of-pocket)**		Source: British National Formulary for medications April 2022

**Medication**	**Unit cost 2022**	**Drug tariff price (one pill)**	**Pills/day based on dose assumption**	**Dose (assumption)**
Ibuprofen 200mg	0.118	0.039	3	maintenance 200–400 mg 3 times a day
Paracetamol 500 mg	0.095	0.024	4	500mg every 4-6 hours
Codeine phosphate 15 mg	0.176	0.029	6	30 mg 3–4 times a day; usual dose 15–60 mg 3–4 times a day
Co-codamol 500mg / 8 mg	0.152	0.038	4	every 4–6 hours
Naproxen 250 mg	0.121	0.040	3	250 mg every 6–8 hours as required
Tramadol hydrocloride 50 mg	0.475	0.119	4	50–100 mg every 4–6 hours
Omeprazole 10 mg	0.332	0.332	1	10 mg once daily,
Antibiotic (Ampicillin 250 mg)	6.946	0.868	8	0.5–1 g every 6 hours.
Dalteparin (Fragmin)*	5.122	5.122	1	5000 units every 12 hours
Amitryptyline hydrocloride 10mg	0.027	0.027	1	Initially 5–10 mg daily, to be taken at night; increased in steps of 10 mg at least every 2 weeks as required; maximum 30 mg per day
Oramorph 10 mg	0.442	0.074	6	Initially 10 mg every 4 hours (acute)
Oxycodone Hydrochlorid 10mg	0.368	0.184	2	Initially 10 mg every 12 hours
Voltaren gel	4.020			
Gabapentin 600mg	0.071	0.047	1.5	Initially 300 mg once daily on day 1, then 300 mg twice daily on day 2, then 300 mg 3 times a day on day 3, alternatively initially 300 mg 3 times a day on day 1, then increased in steps of 300 mg every 2–3 days in 3 divided

For peer review only

				doses, adjusted according to response; maximum 3.6 g per day.
Rivaroxaban 15mg	1.800	1.800	1	10 mg once daily
Ibuprofen gel 100g	2.400			
Co-amoxiclav 500/125mg Tablets	0.264	0.088	3	500/125 mg 3 times a day
Flucoxacillin Capsules 500mg	0.166	0.041	4	500 mg 4 times a day for 5–7 days.
Metronidazole 400mg	0.246	0.082	3	

For peer review only

Table S2. Resource utilisation

	Week 3-6 post surgery						Week 7-12 post surgery					
	Plaster (N=120)			Boots (N=123)			Plaster (N=120)			Boots (N=123)		
	Non-missing	Users	Average use (occasions)	Non-missing	Users	Average use (occasions)	Non-missing	Users	Average use (occasions)	Non-missing	Users	Average use (occasions)
**Physiotherapy services**												
Provided by GP surgery/practice – at the surgery	101	0	0.00	104	3	0.04	77	5	0.10	77	7	0.25
Provided by GP surgery/practice – at your home	102	0	0.00	104	1	0.03	76	0	0.00	77	0	0.00
Provided by a hospital – at hospital	102	0	0.00	105	0	0.00	76	0	0.00	77	0	0.00
Provided by a hospital – at home	102	4	0.05	105	5	0.05	76	2	0.03	77	1	0.01
Provided by a private clinic - at clinic	102	0	0.00	105	2	0.04	76	6	0.28	77	4	0.18
Provided by a private clinic - at home	102	0	0.00	105	0	0.00	76	1	0.04	77	0	0.00
Consultations with an Occupational Therapist	102	5	0.07	105	2	0.05	76	7	0.20	77	11	0.19
**Community-based Services**												
GP at the surgery/practice	102	3	0.04	105	12	0.27	75	7	0.13	75	10	0.28
GP at your home	102	0	0.00	105	2	0.02	75	0	0.00	75	1	0.01
GP telephone contact	102	14	0.20	105	16	0.29	75	5	0.11	75	10	0.19
Practice Nurse / Health Care Assistant at GP surgery	100	0	0.00	104	9	0.09	75	0	0.00	75	5	0.07
Practice Nurse telephone contact	100	0	0.00	103	4	0.07	75	0	0.00	75	0	0.00
Nurse at your home	101	3	0.58	104	5	0.31	75	0	0.00	75	0	0.00
Health Visitor	100	1	0.01	104	0	0.00	75	0	0.00	75	0	0.00
Social worker (all contacts, including telephone calls)	100	0	0.00	104	0	0.00	75	0	0.00	75	0	0.00
Home care/Home help	100	1	0.01	104	4	0.20	75	0	0.00	75	0	0.00

For peer review only

Food at home services (e.g. meals on wheels)	100	0	0.00	104	3	0.09	75	0	0.00	75	74	3.95
Voluntary Agency Worker/Contact	100	0	0.00	103	1	0.02	75	0	0.00	75	0	0.00
**Hospital Services**												
Accident and Emergency	102	5	0.05	105	4	0.05	75	0	0.00	76	0	0.00
Day case admission or procedure (but did not spend the night)	102	1	0.01	105	1	0.01	75	0	0.00	76	0	0.00
Admitted to hospital overnight	98	0	0.00	102	0	0.00	75	0	0.00	76	0	0.00
Outpatient appointment	99	9	0.10	104	8	0.12	75	11	0.21	76	13	0.00
**Informal care (hours)**	102	81	70.33	105	88	43.92	78	47	33.28	78	49	51.65
**Time off work (hours)**	102	48	67.56	105	60	71.91	77	27	58.71	77	29	39.64
**Use of special equipment**	Non-missing	Users	%	Non-missing	Users	%	Non-missing	Users	%	Non-missing	Users	%
Special equipment private	99	2	2.0%	104	8	7.7%	75	0	0.0%	76	1	1.3%
Special equipment NHS (prescribed)	99	6	6.1%	104	3	2.9%	77	12	15.6%	77	22	28.6%
**Use of medication (Prescribed&out-of-pocket)**	101	45	44.6%	105	64	61.0%	76	34	44.7%	77	36	46.8%
**Private costs**												
Gym sessions and other sport sessions	100	1	1.0%	105	6	5.7%	77	4	5.2%	77	6	7.8%
Child care	100	4	4.0%	105	4	3.8%	76	1	1.3%	77	0	0.0%
Home cleaner	100	9	9.0%	105	16	15.2%	76	3	3.9%	77	4	5.2%
Food delivery (e.g. take away, eating out)	100	23	23.0%	105	21	20.0%	76	5	6.6%	77	9	11.7%
Other	99	3	3.0%	103	16	15.5%	75	1	1.3%	77	4	5.2%
Travel expenses	100	34	34.0%	105	34	32.4%	76	21	27.6%	77	19	24.7%
Other major private expenses	101	4	4.0%	105	3	2.9%	77	4	5.2%	75	5	6.7%

For peer review only

Table S3 Costs and outcomes by trial arm and by perspective on costs (raw data)

**Cost Component**	**Boot (N=123)**		**Plaster (N=120)**		**Difference**
NHS+PSS	n	Mean (95% CI)	n	Mean (95% CI)	Mean (95% CI)
Ankle treatment (boot or plaster)	117	£203 (£200, £206)	112	£179 (£177, £181)	£24 (£21, £28)
Physiotherapy in the community	70	£15 (£7, £23)	68	£4 (£1, £7)	£11 (£3, £20)
Physiotherapy in hospital	70	£24 (£13, £35)	68	£25 (£11, £39)	-£1 (-£19, £16)
GP practice services	68	£69 (£37, £100)	66	£33 (£7, £59)	£36 (-£5, £77)
Social Care services	68	£24 (£11, £36)	66	£7 (-£0, £14)	£17 (£2, £31)
A&E and Outpatient services	68	£60 (£24, £96)	65	£29 (£17, £40)	£31 (-£7, £70)
Hospital Admissions	66	£14 (-£6, £34)	66	£14 (-£6, £34)	£0 (-£28, £28)
NHS Equipment	69	£4 (£2, £6)	68	£2 (£1, £2)	£2 (-£0, £4)
Medications	70	£6 (£4, £8)	68	£6 (£1, £10)	£1 (-£4, £5)
Societal costs					
Private physiotherapy	70	£18 (£5, £30)	68	£19 (£7, £31)	-£2 (-£19, £16)
Additional medical Equipment	68	£8 (£3, £13)	66	£0 (-£0, £1)	£8 (£3, £12)
Private community care	104	£0 (-£0, £1)	100	£2 (-£1, £4)	-£1 (-£4, £2)
Other private expenditures on services/activities	67	£105 (£74, £136)	67	£170 (£30, £311)	-£65 (-£207, £77)
Other major expenditure not reported elsewhere	68	£11 (-£0, £23)	68	£11 (£2, £21)	-£0 (-£15, £15)
Productivity Loss (time-off work and leisure)	70	£1,915 (£1,493, £2,337)	69	£2,164 (£1,565, £2,764)	-£249 (-£976, £478)
Informal Care	71	£1,042 (£652, £1,432)	70	£1,185 (£719, £1,650)	-£143 (-£746, £460)
Outcomes					
QALYs gained (Hernandez Alava and Pudney)	60	0.1168 (0.1114, 0.1223)	61	0.1246 (0.1203, 0.1288)	-0.0077 (-0.0146, -0.0009)
QALY gained (Van Hout)	60	0.1086 (0.1027, 0.1144)	61	0.1184 (0.1140, 0.1229)	-0.0099 (-0.0172, -0.0026)
Orelud and Molander (primary outcome, Week7-Week2)	93	-55.4 (-59.5, -51.3)	91	-59.0 (-62.3, -55.7)	3.6 (-1.6, 8.8)
Orelud and Molander (Week12-Week2)	78	-41.3 (-45.3, -37.4)	74	-36.1 (-39.5, -32.7)	-5.3 (-10.5, 0.0)

Note: Costs are in 2020/21 prices.

For peer review only

Figure S1 – Distribution of costs: total NHS+PSS, total societal costs, productivity loss and cost of informal care.

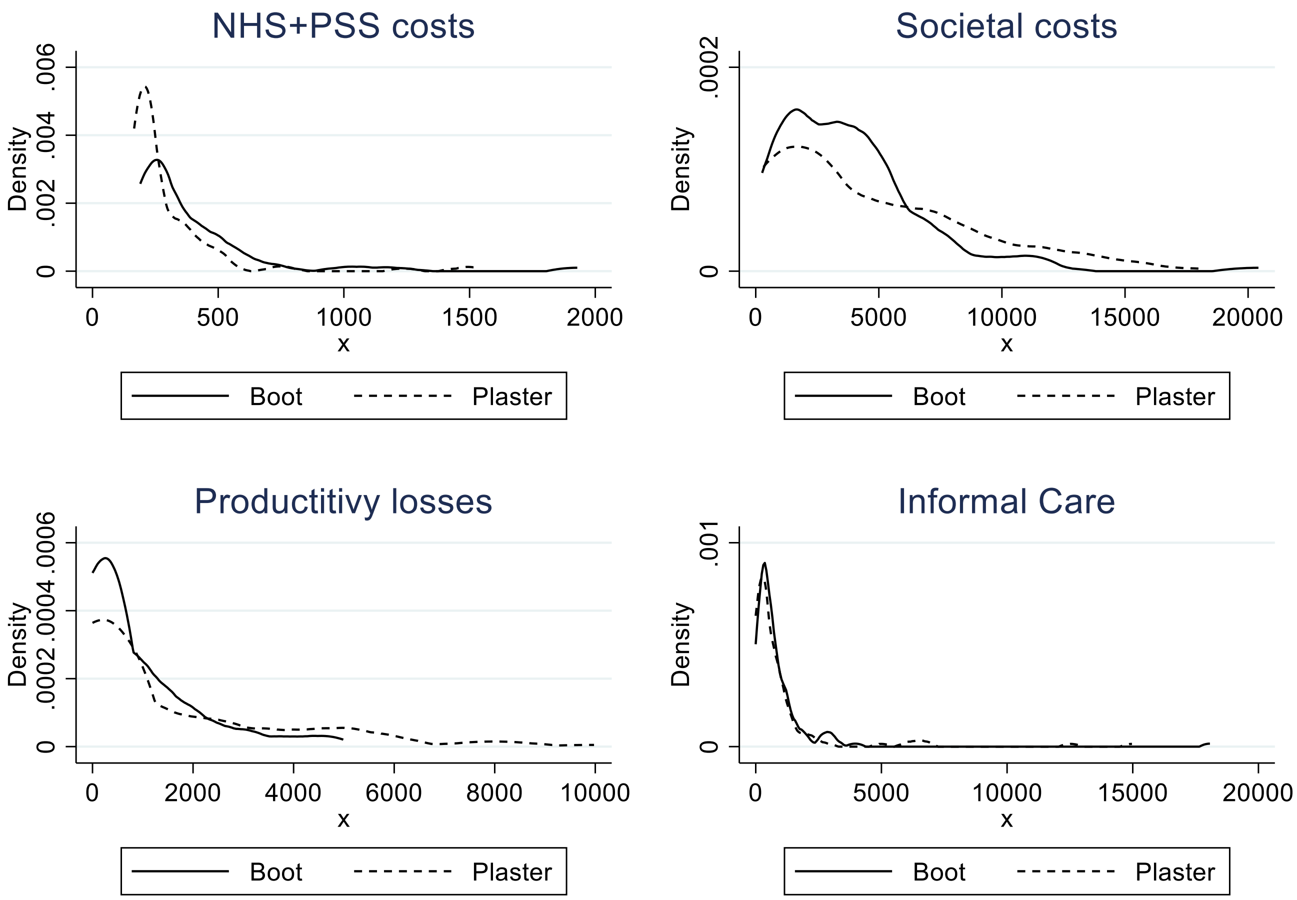

Figure S2 – Distribution of utility scores for QALY gains

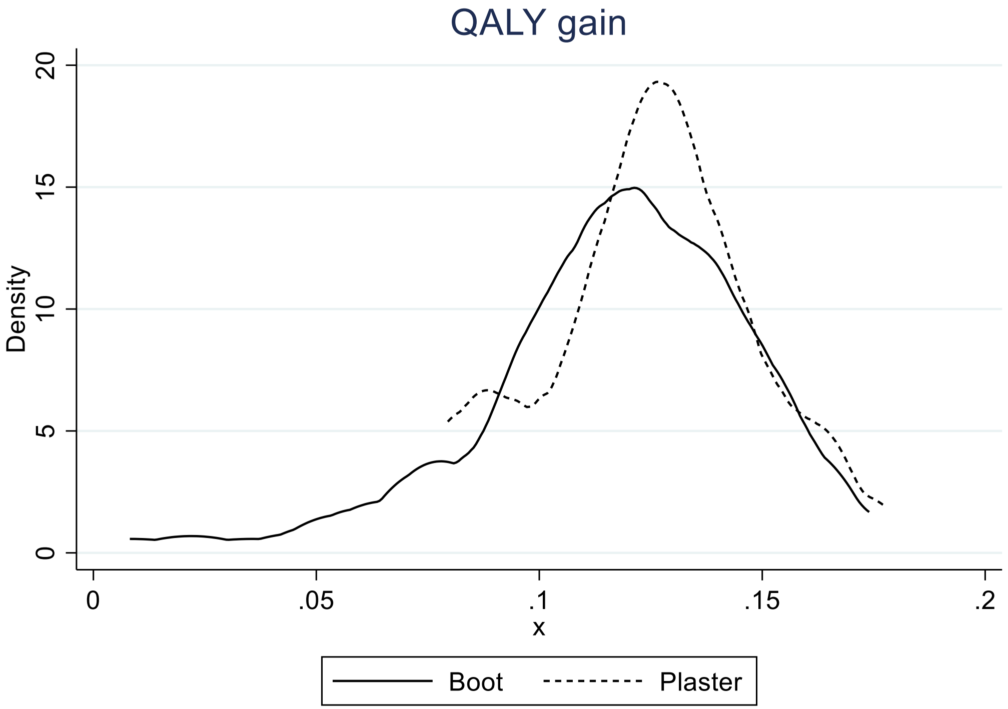

Figure S3 Cost-effectiveness Plane comparing boot (intervention) to cast (control)

a) NHS + PSS perspective

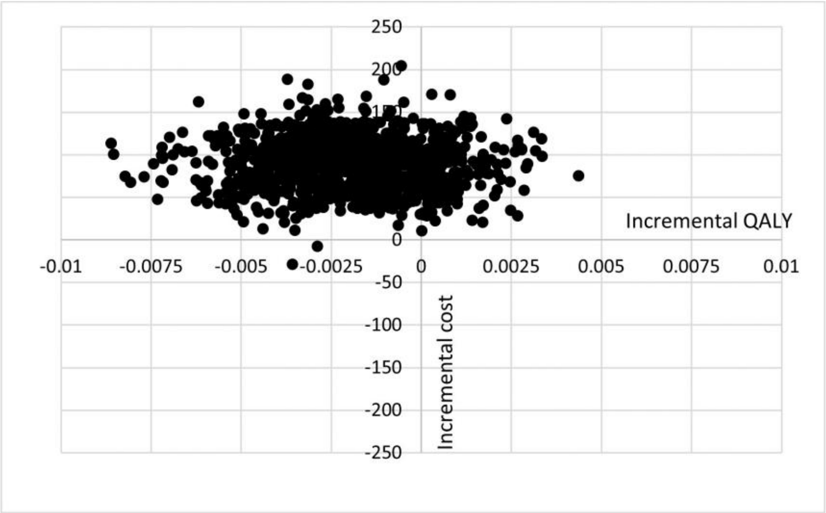

b) Societal perspective

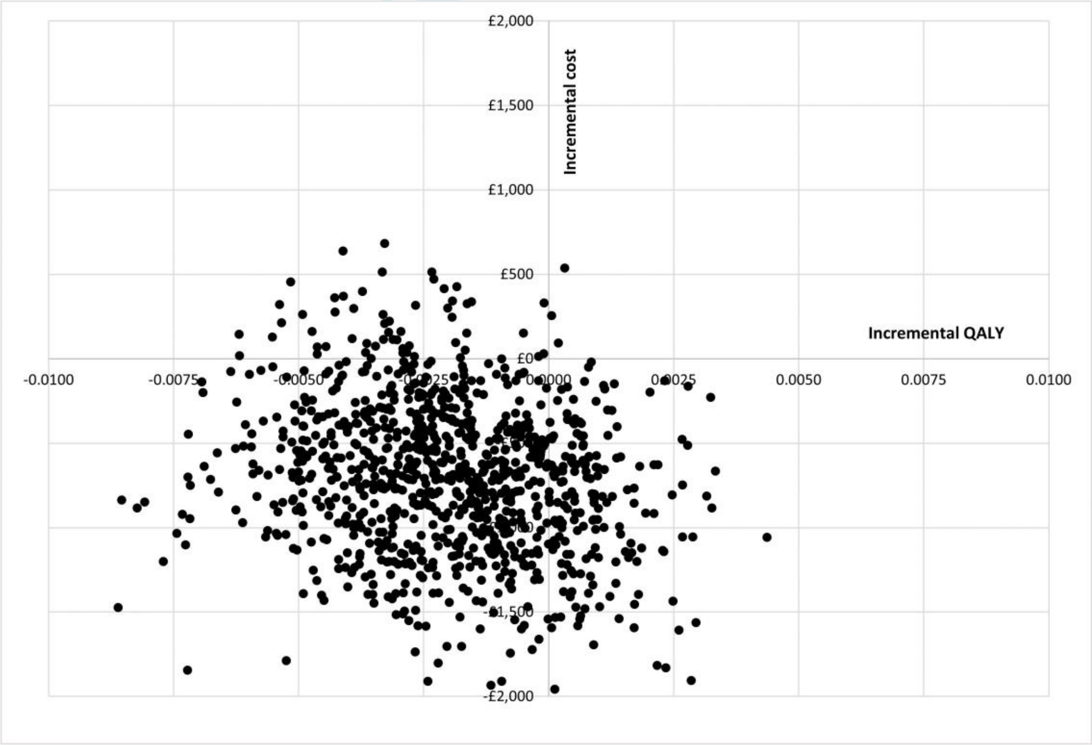

Figure S4 Cost-effectiveness Acceptability Curve comparing boot (intervention) to cast (control)

(a) NHS + PSS perspective

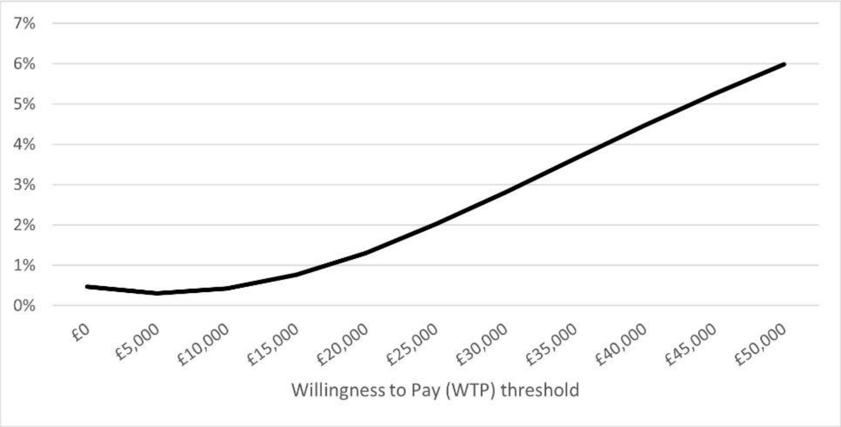

(b) Societal perspective

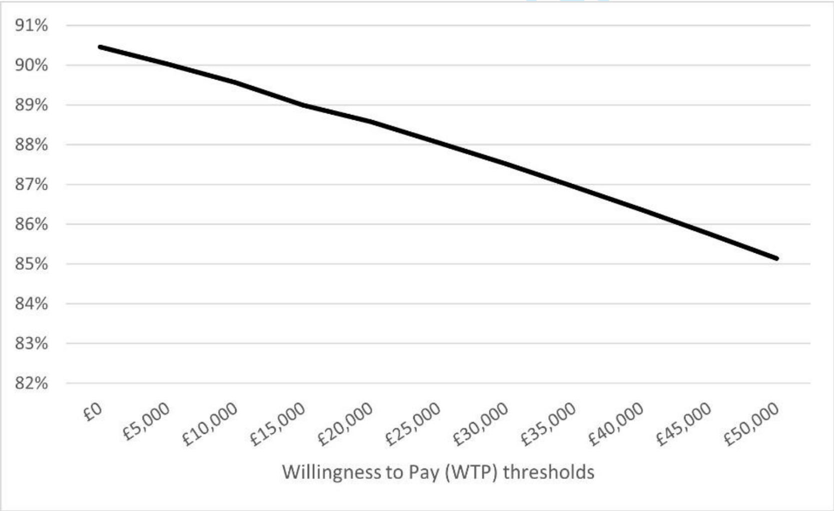

For peer review only - http://bmjopen.bmj.com/site/about/guidelines.xhtml

Table S5 One-way sensitivity analysis

**NHS+PSS perspective**<br>**One-way sensitivity analysis**	**Scenario 1**<br>**25% reduction in boots price**<br>Mean (95%CI)	**Scenario 2**<br>**50% reduction in boots price**<br>Mean (95%CI)	**Scenario 3**<br>**75% reduction in boots price**<br>Mean (95%CI)
Incremental Cost	£76 (£9 to £142)	£63 (-£3 to £130)	£51 (-£16 to £117)
Incremental QALY	-0.0020 (-0.0067 to 0.0026)	-0.0020 (-0.0067 to 0.0026)	-0.0020 (-0.0067 to 0.0026)
Net Monetary Benefit	-£117 (-£218 to -£15)	-£104 (-£206 to -£3)	-£92 (-£193 to £10)
Probability of Cost-effectiveness	2.2%	3.6%	5.7%

For peer review only

CHEERS 2022 Checklist

Topic	No.	Item	Location where item is reported
**Title**			
	1	Identify the study as an economic evaluation and specify the interventions being compared.	Title, Page 1
**Abstract**			
	2	Provide a structured summary that highlights context, key methods, results, and alternative analyses.	Abstract, Page 3
**Introduction**			
**Background and objectives**	3	Give the context for the study, the study question, and its practical relevance for decision making in policy or practice.	Background, 2nd and 3rd paragraphs
**Methods**			
**Health economic analysis plan**	4	Indicate whether a health economic analysis plan was developed and where available.	Methods, Overview of economic evaluation
**Study population**	5	Describe characteristics of the study population (such as age range, demographics, socioeconomic, or clinical characteristics).	Table 1
**Setting and location**	6	Provide relevant contextual information that may influence findings.	Methods, Trial design and ethics approval
**Comparators**	7	Describe the interventions or strategies being compared and why chosen.	Methods, Intervention and usual care
**Perspective**	8	State the perspective(s) adopted by the study and why chosen.	Methods, Overview of economic evaluation
**Time horizon**	9	State the time horizon for the study and why appropriate.	Methods, Trial design and ethics approval
**Discount rate**	10	Report the discount rate(s) and reason chosen.	Not used
**Selection of outcomes**	11	Describe what outcomes were used as the measure(s) of benefit(s) and harm(s).	Methods, Trial design and ethics approval

Topic	No.	Item	Location where item is reported
**Measurement of outcomes**	12	Describe how outcomes used to capture benefit(s) and harm(s) were measured.	Methods, Outcome data collection for the economic evaluation
**Valuation of outcomes**	13	Describe the population and methods used to measure and value outcomes.	Methods, Valuing EQ-5D-5L to derive QALYs
**Measurement and valuation of resources and costs**	14	Describe how costs were valued.	Methods, Valuing resource use to derive costs
**Currency, price date, and conversion**	15	Report the dates of the estimated resource quantities and unit costs, plus the currency and year of conversion.	Methods, Valuing resource use to derive costs
**Rationale and description of model**	16	If modelling is used, describe in detail and why used. Report if the model is publicly available and where it can be accessed.	Not used
**Analytics and assumptions**	17	Describe any methods for analysing or statistically transforming data, any extrapolation methods, and approaches for validating any model used.	Methods, Cost-Utility Analysis, Uncertainty and sensitivity analyses and Appendix
**Characterising heterogeneity**	18	Describe any methods used for estimating how the results of the study vary for subgroups.	Subgroup analysis was not preformed
**Characterising distributional effects**	19	Describe how impacts are distributed across different individuals or adjustments made to reflect priority populations.	Not analysed
**Characterising uncertainty**	20	Describe methods to characterise any sources of uncertainty in the analysis.	Methods , Cost-Utility Analysis, Uncertainty and sensitivity analyses
**Approach to engagement with patients and others affected by the study**	21	Describe any approaches to engage patients or service recipients, the general public, communities, or stakeholders (such as clinicians or payers) in the design of the study.	Methods, Patient and Public Involvement
**Results**			
**Study parameters**	22	Report all analytic inputs (such as values, ranges, references) including uncertainty or distributional assumptions.	Results, first paragraph and Table

Topic	No.	Item	Location where item is reported
**Summary of main results**	23	Report the mean values for the main categories of costs and outcomes of interest and summarise them in the most appropriate overall measure.	Results, Costs and outcomes and Cost-effectiveness base case and sensitivity analyses, Table 2, Table 3
**Effect of uncertainty**	24	Describe how uncertainty about analytic judgments, inputs, or projections affect findings. Report the effect of choice of discount rate and time horizon, if applicable.	Results, Cost-effectiveness base case and sensitivity analyses, Figures 1-2 and Appendix
**Effect of engagement with patients and others affected by the study**	25	Report on any difference patient/service recipient, general public, community, or stakeholder involvement made to the approach or findings of the study	Not reported
**Discussion**			
**Study findings, limitations, generalisability, and current knowledge**	26	Report key findings, limitations, ethical or equity considerations not captured, and how these could affect patients, policy, or practice.	Discussion, Strengths and limitations
**Other relevant information**			
**Source of funding**	27	Describe how the study was funded and any role of the funder in the identification, design, conduct, and reporting of the analysis	End of manuscript
**Conflicts of interest**	28	Report authors conflicts of interest according to journal or International Committee of Medical Journal Editors requirements.	Submitted in the editorial system

From: Husereau D, Drummond M, Augustovski F, et al. Consolidated Health Economic Evaluation Reporting Standards 2022 (CHEERS 2022) Explanation and Elaboration: A Report of the ISPOR CHEERS II Good Practices Task Force. Value Health 2022;25. doi:10.1016/j.jval.2021.10.008

For peer review only - http://bmjopen.bmj.com/site/about/guidelines.xhtml
